# Variation in the microbiome of the urogenital tract of *Chlamydia*-free female koalas (*Phascolarctos cinereus*) with and without ‘wet bottom’

**DOI:** 10.1371/journal.pone.0194881

**Published:** 2018-03-26

**Authors:** Alistair R. Legione, Jemima Amery-Gale, Michael Lynch, Leesa Haynes, James R. Gilkerson, Fiona M. Sansom, Joanne M. Devlin

**Affiliations:** 1 Asia Pacific Centre for Animal Health, The University of Melbourne, Parkville, Victoria, Australia; 2 Veterinary Department, Melbourne Zoo, Parkville, Victoria, Australia; 3 Faculty of Veterinary and Agricultural Sciences, The University of Melbourne, Werribee, Victoria, Australia; 4 Centre for Equine Infectious Diseases, The University of Melbourne, Parkville, Victoria, Australia; Midwestern University, UNITED STATES

## Abstract

Koalas (*Phascolarctos cinereus*) are iconic Australian marsupials currently threatened by several processes, including infectious diseases and ecological disruption. Infection with *Chlamydia pecorum*, is considered a key driver of population decline. The clinical sign of ‘wet bottom’, a staining of the rump associated with urinary incontinence, is often caused by chlamydial urinary tract infections. However, wet bottom has been recorded in koalas free of *C*. *pecorum*, suggesting other causative agents in those individuals. We used 16S rRNA diversity profiling to investigate the microbiome of the urogenital tract of ten female koalas in order to identify potential causative agents of wet bottom, other than *C*. *pecorum*. Five urogenital samples were processed from koalas presenting with wet bottom and five were clinically normal. All koalas were negative for *C*. *pecorum* infection. We detected thirteen phyla across the ten samples, with *Firmicutes* occurring at the highest relative abundance (77.6%). The order *Lactobacillales*, within the *Firmicutes*, comprised 70.3% of the reads from all samples. After normalising reads using DESeq2 and testing for significant differences (*P* < 0.05), there were 25 operational taxonomic units (OTUs) more commonly found in one group over the other. The families *Aerococcaceae* and *Tissierellaceae* both had four significantly differentially abundant OTUs. These four *Tissierellaceae* OTUs were all significantly more abundant in koalas with wet bottom. This study provides the foundation for future investigations of causes of koala wet bottom, other than *C*. *pecorum* infection. This is of clinical relevance as wet bottom is often assumed to be caused by *C*. *pecorum* and treated accordingly. Our research highlights that other organisms may be causing wet bottom, and these potential aetiological agents need to be further investigated to fully address the problems this species faces.

## Introduction

The koala (*Phascolarctos cinereus*) is an iconic marsupial species endemic to Australia. Northern koala populations, in the states of Queensland and New South Wales, are currently declining due to impacts from disease and increased urbanisation. A significant pathogen of koalas, *Chlamydia pecorum*, has been a main focus of koala infectious disease investigations since its discovery. *C*. *pecorum* has been commonly described as the causative agent of the clinical sign known as ‘wet bottom’ [1–4]. This staining, or scalding, of the rump is associated with cystitis due to *C*. *pecorum* infection in some populations [5], but recently samples from a large number of koalas from Victorian populations with mild wet bottom were negative via qPCR for *C*. *pecorum* [6]. In particular, koalas on French Island, a population considered at the time to be free of *C*. *pecorum* [7], had a similar prevalence and severity of wet bottom to populations where *C*. *pecorum* occurred in more than 35% of koalas tested. Further research demonstrated that whilst wet bottom was significantly associated with the detection of *C*. *pecorum* infection in male Victorian koalas, this relationship was not significant in females, leading to the hypothesis that other, unidentified organisms may be causing these mild clinical signs of disease [8]. Recently a study of Queensland koalas infected with *C*. *pecorum* infection revealed that the urogenital microbial diversity was reduced in those animals, compared to those free from *C*. *pecorum* infection [9]. However, it is not known if this is also true for individuals suffering from clinical disease (wet bottom) not caused by *C*. *pecorum*, as analysis of *C*. *pecorum* negative animals with wet bottom was not reported in the study. Modern sequencing technology, specifically 16S rRNA biodiversity profiling, can be used to improve our understanding of the microbiome of the urogenital tract of koalas, and allow preliminary comparisons of the microbiome of the urogenital tract of female koalas with and without mild wet bottom. This study made use of 16S rRNA biodiversity profiling to identify potential causative agents of wet bottom in female koalas, other than *C*. *pecorum*.

## Methods

### Sample collection and initial screening

Samples used in this study were urogenital swabs, from female koalas, stored in Buffer RLT (Qiagen) containing β-mercaptoethanol, taken from an archive of koala samples collected in 2011 from French Island, Victoria, Australia (38°21’0” S, 145°22’12” E). Koala samples were collected under general anaesthetic by veterinarians and trained field assistants during routine population management exercises and clinical health of koalas was recorded at the time. Sample collection was approved by the University of Melbourne Faculty of Veterinary Science Animal Ethics Committee, application ID:1011687.1, and all sample collection was conducted following the Australian code for the care and use of animals for scientific purposes, 8th edition [10]. Wet bottom score was assessed using a scoring system that grades the clinical findings relating to wet bottom from 0 (absent) to 10 (most severe) as previously described [11]. After screening all samples for *Chlamydiaceae* using a previously described qPCR (16SG) targeting the 16S rRNA [12], which has previously been used to detect *C*. *pecorum* in koala swab samples [6, 8, 13], we selected ten samples from female koalas where no *Chlamydiaceae* was detected. Five samples were selected from koalas showing no clinical signs of urogenital disease and five samples were selected from koalas that showed clinical signs of wet bottom (Table 1). All samples used from koalas with wet bottom present had wet bottom scores of greater or equal to 2. Avoiding samples from animals with a wet bottom score of 1 minimises the chance of a false diagnosis, as a score of 2 indicates increased margins of discolouration of fur around the cloaca, more persistent urine leakage during examination and a discernible odour at the site. Scores of 3 and 4 involve an increase in the severity of these signs, as well as inflammation of the cloacal region and obvious build-up of calculus [11]. No scores above 5, which involve cases of blood in urine and/or scalding of the rump, were identified in this cohort, as is typical for southern koala populations [6]

**Table 1:** Koala wet bottom scores, sequencing metrics, and relative abundance.

Koala/Sample name	K1	K2	K3	K4	K5	K31	K49	K55	K59	K70
**Wet bottom score**^#^	0	0	0	0	0	2	3	3	4	3
**Merged reads**	253256	211620	186912	220410	185592	183126	199985	263685	216495	300448
**Reads after filtering**	156100	134940	118418	132125	112823	110292	116321	160328	136996	169169
**Reads clustered to OTUs**	225868	178678	169576	203062	166906	162343	177452	216270	192105	254327
**Absolute OTUs**	93	66	86	89	74	55	61	74	76	126
**Standardised OTUs**^^^ **± SD**	88.8 ± 1.7	64.1 ± 1.2	85.4 ± 0.7	88 ± 0.9	73.7 ± 0.6	54.9 ± 0.3	59.2 ± 1.4	69.2 ± 1.9	72.9 ± 1.5	123.4 ± 1.3
**Phyla**^&^										
***Acidobacteria***	-	-	-	-	< 0.01%	-	-	-	-	0.01%
***Actinobacteria***	5.47%	9.06%	2.92%	0.17%	0.03%	3.27%	0.66%	1.50%	0.30%	0.19%
***Armatimonadetes***	< 0.01%	< 0.01%	-	-	< 0.01%	-	-	-	-	-
***Bacteroidetes***	0.57%	0.05%	2.14%	1.72%	0.21%	0.33%	0.05%	9.05%	1.00%	50.53%
***Cyanobacteria***	< 0.01%	-	< 0.01%	-	-	-	-	-	-	0.02%
***Deferribacteres***	-	-	-	-	-	-	-	-	-	< 0.01%
***Firmicutes***	92.92%	89.57%	85.67%	79.17%	98.92%	80.35%	40.92%	84.88%	95.65%	39.09%
***Fusobacteria***	0.02%	< 0.01%	< 0.01%	0.07%	< 0.01%	< 0.01%	-	< 0.01%	0.02%	1.09%
***Planctomycetes***	-	-	< 0.01%	-	0.01%	-	-	-	< 0.01%	0.80%
***Proteobacteria***	0.24%	0.15%	1.66%	1.51%	0.45%	0.23%	56.90%	0.19%	2.37%	2.70%
***Synergistetes***	0.08%	0.02%	0.30%	0.31%	0.01%	-	-	< 0.01%	0.02%	4.35%
**TM7**	0.02%	0.50%	0.21%	-	< 0.01%	1.38%	0.05%	2.86%	< 0.01%	0.02%
***Verrucomicrobia***	< 0.01%	< 0.01%	< 0.01%	-	0.02%	< 0.01%	-	-	0.01%	0.69%
**Unassigned**	0.69%	0.65%	7.07%	17.04%	0.34%	14.44%	1.42%	1.52%	0.61%	0.52%

Koala wet bottom score, read metrics and relative abundance data from ten samples submitted for 16S rRNA amplicon sequencing. All koalas were female and sampled from French Island, Victoria, Australia in 2011.

^#^ Wet bottom score ranges from 0 (absent) to 10 (most severe) [11]

^^^ The average number of OTUs detected in 100 iterations of subsampling to a depth of 160,000 reads

^&^ Phyla assigned using QIIME [14] script **assign_taxonomy.py** utilising Greengenes [15] curated 16S rRNA library

### Amplification and sequencing

DNA extraction and amplification from the swab samples was performed commercially by The Australian Genome Research Facility (AGRF) (Australia). Variable regions three and four (V3-V4) of bacterial 16S rRNA were amplified using primers 341F (5’ CCTAYGGGRBGCASCAG 3’) and 806R (5’ GGACTACNNGGGTATCTAAT 3’) [16]. Amplification was performed by AGRF in accordance to the Illumina ‘16S Metagenomic Sequencing Library Preparation’ guidelines, which includes a 25 cycle PCR [17]. Sequencing was performed on the Illumina MiSeq platform, utilising Nextera XT v2 Indices and Paired End sequencing chemistry to produce paired end reads of 300 bp (2 × 300 bp). This method allows for an overlap of approximately 130 bp between the forward and reverse reads, which can be used to improve base calling confidence of the 3’ end of each sequence.

### Quality filtering and OTU assignment

Quality filtering and operational taxonomic unit (OTU) assignment was undertaken using a mixture of scripts and algorithms available in the programs USEARCH 8.1 [18] and QIIME 1.9.1 (Quantitative Insights Into Microbial Ecology) [14]. Script names are repeated here in bold for reproducibility and, unless otherwise stated, default settings were used for all scripts. Read processing to reduce errors was undertaken as described by Edgar and Flyvbjerg [19]. The forward and reverse 300 bp paired-end reads for each swab sample were merged using the USEARCH script **fastq_mergepairs**. In this process, the Phred score of overlapping bases are recalculated to improve error calling. Bases with the same nucleotide called in both the forward and reverse reads have an increased recalculated score, and those with disagreements are reduced [19, 20]. This increases confidence in the calculated error probability of the merged reads. Primers were then trimmed from the 5’ and 3’ ends of the merged reads using seqtk (https://github.com/lh3/seqtk). Trimmed reads were filtered for quality using the USEARCH script **fastq_filter**. This script filters reads using the maximum expected errors per merged read. The number of expected errors is obtained by the sum of the Phred derived error probability. If the expected number of errors is less than one, then the most probable number of errors is zero [19]. A maximum expected error threshold of 1 was utilised, resulting in reads with an error probability of 1 or greater being removed. In addition to using the number of expected errors for filtering, trimmed reads shorter than 400 bp were discarded. Unique reads within the entire sample set were clustered into OTUs using the USEARCH algorithms **derep_fulllength** and **cluster_otus** [20], with a minimum identity of 97% for clustering, or a cluster radius of 3.0. Chimeras are filtered from the sample set *de novo* within the **cluster_otus** command using the UPARSE-REF maximum parsimony algorithm [20]. Singletons were excluded from OTU clustering due to the high likelihood that they contain errors [19, 20]. To obtain read counts for OTUs, the merged/trimmed reads from each swab sample, including the previously excluded singletons and merged reads shorter than 400 bp, were matched to the clustered OTUs using USEARCH script **usearch_global**, with a threshold of 97% identity to group a read into a specific OTU. The taxonomy of each OTU was estimated by using the QIIME script **assign_taxonomy.py** in conjunction with the Greengenes taxonomy database (version 13_5, 97% clustered OTUs) [15]. This script utilises the UCLUST algorithm [18] to identify a consensus taxonomy of the reads within an OTU against the curated database, based on a similarity of 90% and a minimum consensus fraction of 0.51 of the three best hits. Chloroplast and mitochondrial OTUs were removed from the dataset using the QIIME script **filter_taxa_from_otu_table.py**.

### Read normalisation and analysis

Read data was assessed using three different methods. Relative abundance was utilised to compare basic phylum presence in each sample. Rarefaction of reads was undertaken, using **multiple_rarefactions.py** QIIME script, to assess alpha and beta diversity at a set read level. Negative-binomial normalisation of reads, using DESeq2 [21] as recommended by McMurdie and Holmes [22], was performed using the QIIME script **normalize_table.py**. For rarefactions, reads within each sample were subsampled (without replacement) every 5000 reads, from 5000 to 250,000 reads. This represented the maximum number of reads present in the sample with the most reads (rounded down to the nearest value divisible by 5,000). At each step, 100 permutations were undertaken. Alpha-diversity metrics were generated for each step for OTU richness (OTU abundance and Chao1 [23]) and OTU diversity (phylogenetic diversity [24] and Shannon’s diversity [25]). Comparisons of these values were undertaken using values obtained after subsampling to a depth of 160,000. This equalled the sample with the fewest reads (rounded down to the nearest value divisible by 5,000). Non-parametric comparisons of mean alpha-diversity metrics between the two sample groups (wet bottom present or absent) were undertaken with the **compare_alpha_diversity.py** QIIME script. This utilised a non-parametric two sample t-test with 10,000 Monte Carlo permutations to determine whether the mean alpha-diversity was significantly different between the two groups (wet bottom present/absent) at a depth of 160,000 reads. Beta-diversity was assessed at the same depth as above (160,000 reads) using the **beta_diversity_through_plots.py** QIIME script, in which both unweighted (OTU richness) and weighted (OTU diversity) UniFrac distances [26] were assessed. Bray-Curtis dissimilarity [27] between samples was also assessed as a measure for OTU richness between groups. The analysis of beta-diversity requires a phylogenetic tree. For this, an alignment of representative sequences of each OTU was created with PyNAST [28] and UCLUST using the **align_seqs.py** QIIME script. A tree was produced from this alignment using FastTree [29], and used as input for beta-diversity analysis. **beta_diversity_through_plots.py** produced distance matrices for each of the tests (UniFrac and Bray-Curtis), from which principal coordinates and eigen values could be calculated. PCoA plots using the 2 or 3 most influential principal coordinates were drawn from the resulting distance matrices either using either the **make_2d_plots.py** QIIME script, or within the **beta_diversity_through_plots.py** script using EMPeror 9.51 software [30], respectively. Distance and dissimilarity metrics were used to compare the microbial communities between the two groups by utilising the permutational ANOVA (PERMANOVA) [31] method within the **compare_categories.py** QIIME script, with 10,000 permutations. Statistical comparisons of the differential abundance of OTUs between koalas with and without wet bottom utilised DESeq2 within the QIIME script **differential_abundance.py**. These comparisons aimed to determine OTUs which were over-represented in either group. Statistically significant results, from the negative binomial Wald test within DESeq2, were based on *P*-values < 0.05, and were adjusted for false discovery within the script, using the method described by Benjamini and Hochberg [32].

Whilst OTUs are typically difficult to classify to a species level, for exploratory purposes the NCBI nucleotide database [33] was utilised to search for best hits to significantly differentially abundant OTUs. This was conducted using the representative sequence of the significant OTU and the MegaBLAST algorithm [34], excluding uncultured sample sequences.

Illumina reads for each sample used in this study are available to download from the NCBI Sequence Read Archive (Accession numbers: SRX2464137 –SRX246146).

## Results

### Clinical status of koalas

Of the five koalas with wet bottom used in this study, the median wet bottom clinical score was 3 (range: 2–4). The five clinically healthy animals all had wet bottom clinical scores of zero. All koalas were negative for *Chlamydiaceae* using a pan-*Chlamydiaceae* qPCR.

### Analysis and processing of sequencing data

**Fig 1:**
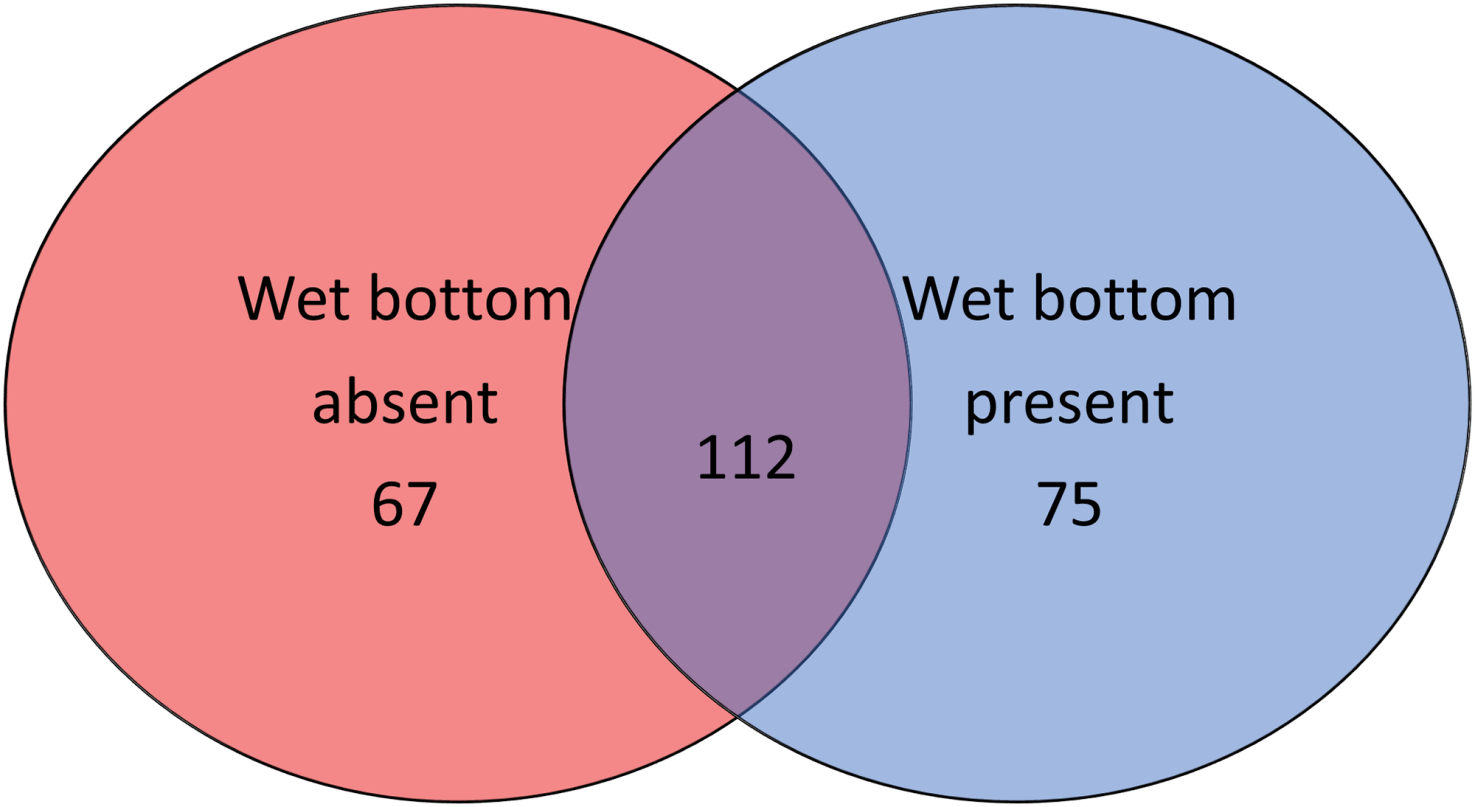
OTUs detected in koalas with or without wet bottom. Venn diagram of the total operational taxonomic units (OTUs) detected in koalas with or without wet bottom. Overlap does not scale with OTU number.

A total of 2,295,607 paired reads were obtained across the ten samples, ranging between 189,315 to 312,131 reads per sample. The GC content of the reads was 51.8%. Merging paired reads, trimming 5’ and 3’ ends, quality filtering to remove errors and discarding merged sequences shorter than 400 bp resulted in a total of 1,347,512 reads suitable for OTU clustering. Dereplication of those reads resulted in 275,642 unique reads for clustering into OTUs. Through the clustering process, it was determined that 3953 unique reads were chimeric, representing 24,376 filtered reads. The non-chimeric unique reads were clustered into 261 OTUs, 7 of which were either chloroplasts or mitochondria and were subsequently removed from the analysis, resulting in 254 OTUs used in analysis. In total 1,946,587 reads, from 2,221,529 merged reads (87.6%) were matched to the clustered OTUs. Within samples, this ranged from 162,343 (82% of available reads) to 254,327 (92.1% of available reads) (Table 1). Fewer than half of the OTUs detected across the two sample groups were shared between them (112/254) (Fig 1). For comparison, the same filtering and clustering methodology was run without the removal of singletons, which resulted in the clustering of reads into 592 OTUs, suggesting that 331 unique sequences of acceptable quality occurred only once.

### Phylum presence and relative abundance

In total, 13 phyla were detected in the ten samples (Table 1), with *Firmicutes* occurring at the highest relative abundance (77.61%). Just over a third of the OTUs were classified as *Firmicutes* (95/254), followed by *Proteobacteria* (59/254) and the *Bacteroidetes* (35/254). When samples were split into the two groups, koalas without wet bottom had 89.3% of reads classified as *Firmicutes*, followed by OTUs which could not be assigned using the 90% similarity threshold (5.2%) and *Actinobacteria* (3.5%). Koalas with wet bottom had 68.2% reads assigned to OTUs classified as *Firmicutes*. The next two most prevalent phyla were *Proteobacteria* (12.5%) and *Bacteroidetes* (12.2%), however these phyla were over-represented in two samples, biasing the total relative values. *Deferribacteres* were detected in only one sample (Koala 70, wet bottom present) and *Acidobacteria* were only detected in two (one clinically normal koala and one displaying wet bottom). *Armatimonadetes* was detected in three koalas without wet bottom, but in none of the five diseased koalas. These three phyla were detected at the lowest relative abundance across the ten samples. Data for relative read abundance for OTUs that could be taxonomically assigned to a genus level and occurred at a percentage of 0.01% or more in either group can be found in Table 2. This shows that the order *Lactobacillales*, and within that the genus *Aerococcus*, had the highest proportion of relative reads.

**Table 2:** Relative abundance of OTUs.

Phylum	Class	Order	Family	Genus	OTUs	WB absent	WB present	Combined
*Actinobacteria*	*Actinobacteria*	*Actinomycetales*	*Actinomycetaceae*	*Mobiluncus*	1	Nil^^^	0.05%	0.03%
			*Corynebacteriaceae*	*Corynebacterium*	6	0.68%	0.60%	0.64%
*Bacteroidetes*	*Bacteroidia*	*Bacteroidales*	*Bacteroidaceae*	*Bacteroides*	14	0.03%	0.54%	0.29%
			*Porphyromonadaceae*	*Dysgonomonas*	1	<0.01%^+^	0.18%	0.09%
				*Parabacteroides*	7	0.89%	9.55%	5.22%
				*Porphyromonas*	2	<0.01%	1.88%	0.94%
			*Prevotellaceae*	*Prevotella*	2	<0.01%	0.02%	0.01%
*Firmicutes*	*Bacilli*	*Bacillales*	*Staphylococcaceae*	*Staphylococcus*	1	0.02%	<0.01%	0.01%
		*Lactobacillales*	*Aerococcaceae*	*Aerococcus*	6	77.45%	54.74%	66.10%
			*Aerococcaceae*	*Facklamia*	1	6.55%	5.43%	5.99%
			*Carnobacteriaceae*	*Trichococcus*	1	0.02%	0.05%	0.04%
			*Streptococcaceae*	*Streptococcus*	2	0.03%	<0.01%	0.02%
	*Clostridia*	*Clostridiales*	*Tissierellaceae*	*Gallicola*	1	<0.01%	0.27%	0.14%
				*Peptoniphilus*	4	<0.01%	0.53%	0.27%
				*ph2*	3	Nil	0.10%	0.05%
			*Clostridiaceae*	*Clostridium*	8	4.48%	1.87%	3.18%
			*Peptococcaceae*	*Peptococcus*	1	Nil	0.23%	0.11%
			*Ruminococcaceae*	*Ruminococcus*	2	0.07%	0.10%	0.08%
			*Veillonellaceae*	*Dialister*	1	Nil	0.04%	0.02%
				*Phascolarctobacterium*	1	0.04%	1.03%	0.54%
*Fusobacteria*	*Fusobacteriia*	*Fusobacteriales*	*Fusobacteriaceae*	*Fusobacterium*	2	0.02%	0.22%	0.12%
*Proteobacteria*	*Alphaproteobacteria*	*Rhizobiales*	*Methylobacteriaceae*	*Methylobacterium*	2	0.31%	0.06%	0.19%
	*Betaproteobacteria*	*Burkholderiales*	*Alcaligenaceae*	*Sutterella*	1	<0.01%	0.05%	0.02%
	*Deltaproteobacteria*	*Desulfovibrionales*	*Desulfovibrionaceae*	*Desulfovibrio*	2	0.06%	0.12%	0.09%
	*Gammaproteobacteria*	*Pasteurellales*	*Pasteurellaceae*	*Lonepinella*	1	0.06%	0.25%	0.15%
		*Pseudomonadales*	*Moraxellaceae*	*Acinetobacter*	4	0.01%	0.02%	0.01%
			*Pseudomonadaceae*	*Pseudomonas*	2	0.01%	<0.01%	0.01%
*Synergistetes*	*Synergistia*	*Synergistales*	*Synergistaceae*	*vadinCA02*	1	Nil	0.04%	0.02%
*Verrucomicrobia*	*Verrucomicrobiae*	*Verrucomicrobiales*	*Verrucomicrobiaceae*	*Akkermansia*	1	<0.01%	0.14%	0.07%

Relative abundance of OTUs with taxonomic classification shown to a genus level, in female koalas with and without wet bottom (WB). Only OTUs with relative abundance greater than 0.01% in at least one group are shown.

^^^ No reads clustering with OTUs that were assigned this genus were present in any of the 5 koalas within this group

^+^ Less than 0.01% of reads were clustered to OTUs within this genus, but are included in this table due to the converse group having greater than 0.01% of reads clustered to OTUs within this genus.

### Richness and diversity

**Fig 2:**
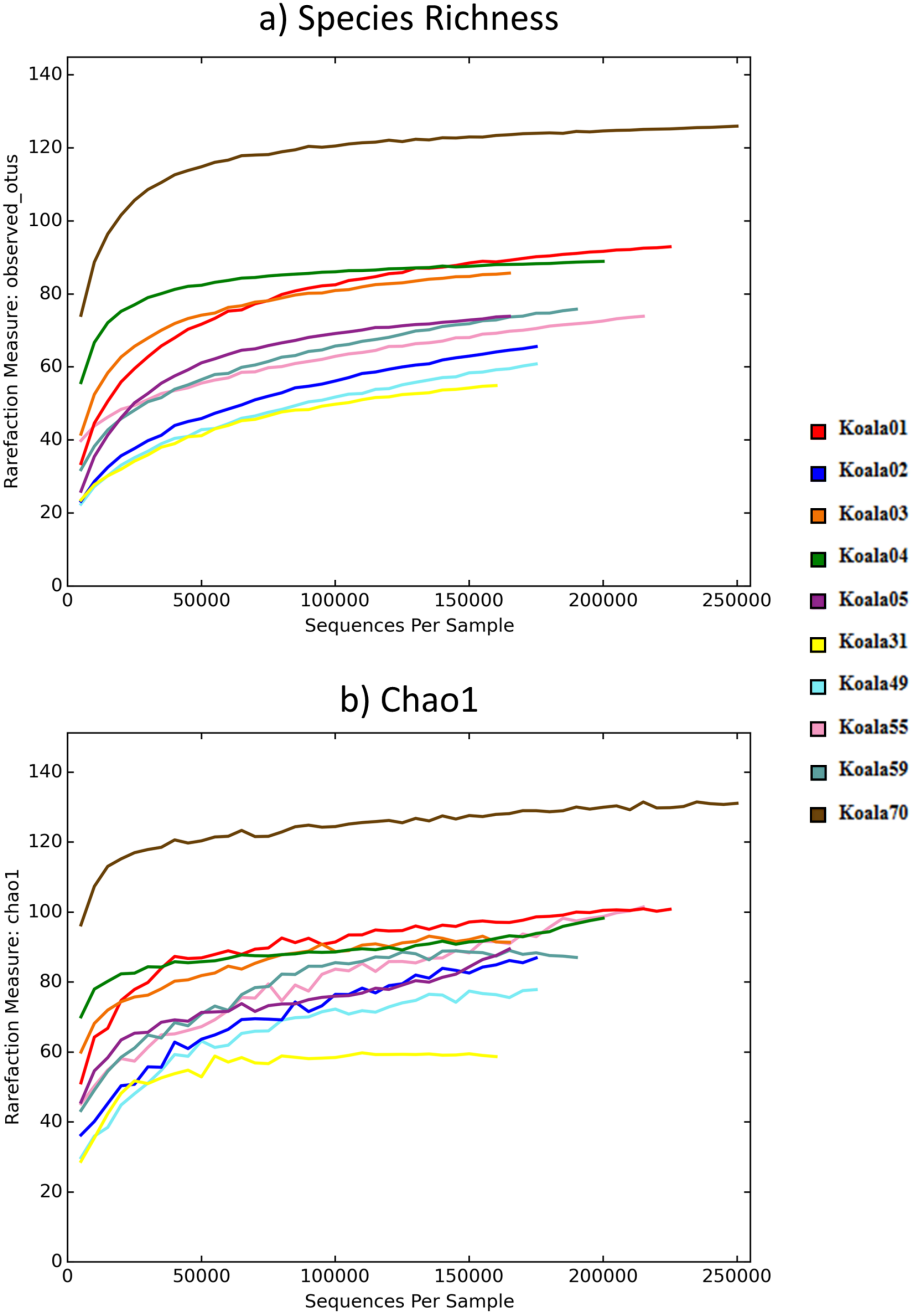
Rarefaction plots showing a) species richness (OTU abundance) and b) Chao1. OTUs were subsampled every 5000 reads, with 100 iterations, with the mean result of these iterations forming the plots. Koalas 1–5 were clinically normal (wet bottom absent), whilst koalas 31–70 had wet bottom.

Species richness within each sample is described in Tables 1 and 3. The mean species richness and Chao1 from 100 iterations of subsampling every 5000 reads is shown in Fig 2. After 100 iterations of rarefaction to a depth of 160,000 reads per sample, the mean number of OTUs in the two groups was 80.0 (standard deviation (SD) ± 9.6) and 75.9 (SD ± 24.6) for koalas with wet bottom and without wet bottom, respectively. The mean values for all alpha-diversity metrics compared between samples from koalas with or without wet bottom were not significantly different. This included richness metrics: observed OTUs (t = -0.31, *P* = 0.81) and Chao1 (with wet bottom group (WB) mean = 90.7, without wet bottom group (NWB) mean = 88.4, t = -0.20, *P* = 0.83); and diversity metrics: phylogenetic diversity (WB mean = 7.8, NWB mean = 8.1, t = -0.39, *P* = 0.71) and Shannon’s diversity (WB mean = 2.4, NWB mean = 2.5, t = -0.15, *P* = 0.86) (see Table 3 for individual sample alpha-diversity values and standard deviations). Results detailing abundance for all OTUs detected in koala urogenital samples, as well as assigned taxonomy are recorded in S1 Table.

**Table 3:** Alpha diversity metrics for microbial communities in the urogenital tract of female koalas with and without wet bottom.

	Richness (OTUs)	Chao1	Shannon’s diversity	Phylogenetic diversity
**Wet bottom absent**				
Koala 1	88.8 (± 1.7) ^#^	97.1 (± 5.9)	2.6 (± <0.01)	9.1 (± 0.2)
Koala 2	64.1 (± 1.2)	84.9 (± 7.4)	2.7 (± <0.01)	7.0 (± 0.1)
Koala 3	85.4 (± 0.7)	91.5 (± 2.7)	3.0 (±<0.01)	8.9 (± 0.1)
Koala 4	88 (± 0.9)	92.5 (± 3.7)	3.1 (± <0.01)	7.7 (± 0.1)
Koala 5	73.7 (± 0.6)	87.6 (± 4.9)	1.1 (± <0.01)	7.9 (± 0.1)
Mean	80.0 (± 9.6)	90.7 (± 4.2)	2.5 (± 0.7)	8.1 (± 0.8)
**Wet bottom present**				
Koala 31	54.9 (± 0.3)	58.7 (± 0.8)	2.4 (± <0.01)	6.5 (± 0.0)
Koala 49	59.2 (± 1.4)	76.4 (± 7.2)	1.4 (± <0.01)	6.5 (± 0.2)
Koala 55	69.2 (± 1.9)	91.5 (± 13.5)	2.3 (± <0.01)	7.8 (± 0.2)
Koala 59	72.9 (± 1.5)	87.4 (± 7.1)	1.8 (± <0.01)	7.8 (± 0.1)
Koala 70	123.4 (± 1.3)	127.9 (± 5.9)	4.1 (± <0.01)	10.4 (± 0.1)
Mean	75.9 (± 24.6)	88.4 (± 22.8)	2.4 (± 0.9)	7.8 (± 1.4)
***t* stat**	-0.31	-0.20	-0.15	-0.39
***P* value**	0.81	0.83	0.86	0.71

All metrics assessed based on OTU values after subsampling to a depth of 160,000 reads, with 100 permutations. *P* values are non-parametric t-tests using 10,000 Monte Carlo permutations.

^#^ All ± values are standard deviation from the mean

**Fig 3:**
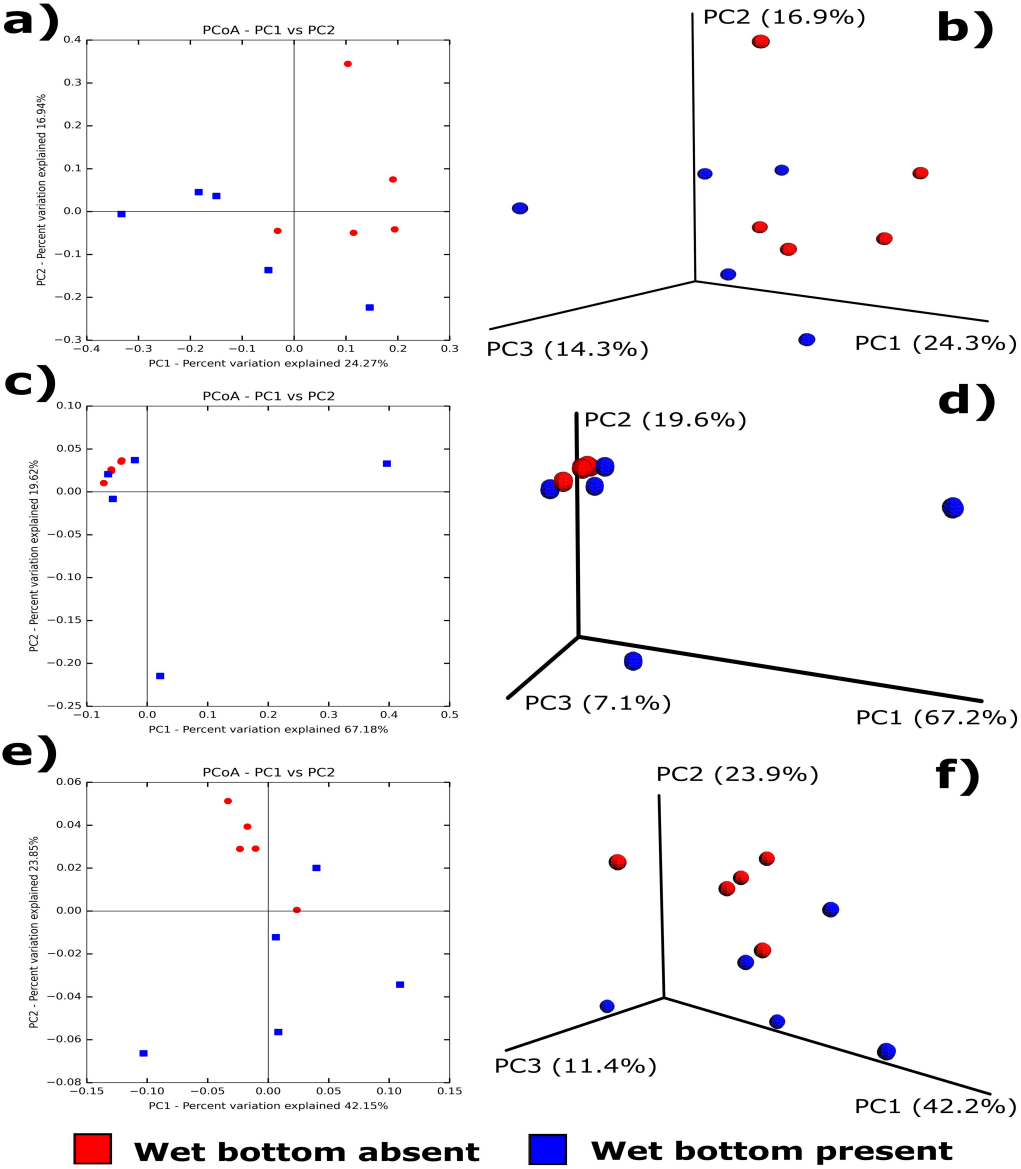
2D and 3D PCoA plots of koala samples, with and without wet bottom. **a/b)** unweighted UniFrac distances of OTUs at a depth of 160,000 reads, **c/d)** weighted UniFrac distances of OTUs at a depth of 160,000, **e/f)** weighted UniFrac distances of normalised reads.

At a read depth of 160,000 there was a significant difference between the diversity of microbial communities in koalas with wet bottom compared to those without, based on the results of a 10,000 permutation PERMANOVA using Bray-Curtis dissimilarity (Pseudo F = 4.92, *P* = 0.019) and unweighted (qualitative) UniFrac distances (Pseudo F = 1.62, *P* = 0.031). There was no significant quantitative, or richness associated, difference detected between the microbial communities of the two groups based on weighted UniFrac distances (Pseudo F = 1.51, *P* = 0.061). 2D and 3D principal coordinate analysis (PCoA) graphs comparing koalas with and without wet bottom are shown in Fig 3. These highlight two outliers in the wet bottom present group, koalas 49 and 70.

### Comparisons between samples using DESeq2 normalised reads

**Fig 4:**
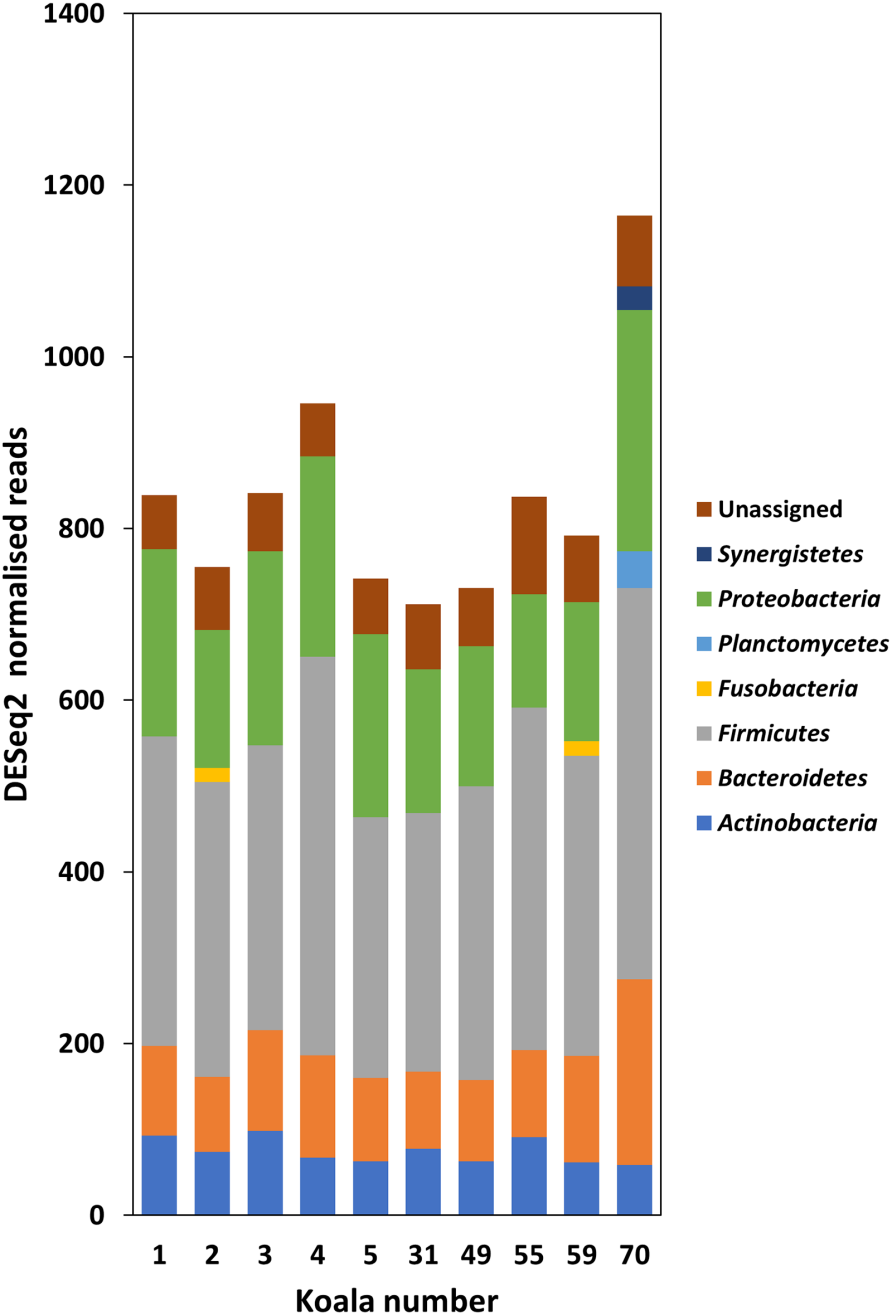
DESeq2 normalised read counts of phyla detected in koala urogenital swab samples. Phyla with fewer than 2% relative reads within each sample have been excluded for clarity. Reads were characterised into taxanomic groups using QIIME [14], utilising Greengenes [15] as a reference database. Koalas 1–5 were clinically normal (wet bottom absent), whilst koalas 31–70 had wet bottom.

Negative binomial normalisation of reads from each sample using DESeq2 still resulted in *Firmicutes* as the most dominant phylum across all samples. This was followed by *Proteobacteria* and *Bacteroidetes* (Fig 4). Overall there were 25 OTUs with significant (Benjamini and Hochberg [32] adjusted *P* < 0.05) over-representation or under-representation in wet bottom affected koalas, in comparison to clinically normal koalas, based on the log fold change of the mean normalised read counts (Table 4). Of those OTUs with significant differences, when assessed in conjunction with absolute read counts, six occurred only in koalas with wet bottom, whilst eight occurred only in koalas without wet bottom (Table 4). Normalised read values for all OTUs, along with assigned taxonomy can be found in S2 Table, and statistical comparisons of normalised reads for all OTUs in relation to wet bottom presence or absence are in S3 Table.

**Table 4:** Significant operational taxonomic units (OTU) assessed using DESeq2 [21], ordered from lowest to highest adjusted *P* value.

OTU ID	Adjusted *P* value *	Higher abundance group ^#^	OTU present in samples/n		Greengenes taxonomic classification^+^	NCBI MegaBLAST best hit^^^		
			WB absent	WB present		Organism	Nucleotide Identity (%)	Accession number
38	< 0.001	WB present	0/5	5/5	g: *Peptoniphilus*	*Peptoniphilus indolicus*	96.8	NR_117566
21	< 0.001	WB present	1/5	5/5	g: *Peptoniphilus*	*Peptoniphilus asaccharolyticus*	100	KP944181
47	< 0.001	WB present	0/5	3/5	g: ph2	*Levyella massiliensis*	100	NR_133039
51	< 0.001	WB present	0/5	3/5	g: *Peptoniphilus*	*Peptoniphilus lacrimalis*	100	KM624632
65	0.001	WB present	1/5	2/5	g: *Sutterella*	*Sutterellaceae bacterium*	99.5	LK054638
86	0.003	WB absent	3/5	0/5	g: *Bacteroides*	*Bacteroides thetaiotaomicron*	100	KU234409
75	0.004	WB absent	2/5	0/5	f: *Lachnospiraceae*	*Clostridium* sp.	96.5	AB622820
4	0.004	WB absent	5/5	5/5	g: *Aerococcus*	*Lactobacillales bacterium*	92.8	HQ115584
70	0.005	WB absent	2/5	0/5	o: *Clostridiales*	*Clostridium neopropionicum*	94.6	JQ897394
73	0.005	WB present	0/5	2/5	f: *Rikenellaceae*	*Alistipes onderdonkii*	93.6	NR_113151
69	0.005	WB absent	2/5	0/5	f: *Lachnospiraceae*	*Lachnospiraceae bacterium*	95.3	EU728729
2	0.006	WB absent	5/5	5/5	g: *Aerococcus*	*Trichococcus* sp.	94.2	KU533824
94	0.007	WB absent	2/5	1/5	f: *Methylocystaceae*	*Rhizobiales* sp.	100	KJ016001
95	0.013	WB absent	2/5	0/5	g: *Rhizobium*	*Rhizobium leguminosarum*	100	KX346599
103	0.019	WB absent	2/5	0/5	Unassigned	*Piscinibacter aquaticus*	88.6	NR_114061
106	0.019	WB absent	3/5	0/5	g: *Burkholderia*	*Burkholderia cenocepacia*	100	KU749979
109	0.019	WB present	0/5	2/5	g: *Peptostreptococcus*	*Peptostreptococcus anaerobius*	94.1	NR_042847
148	0.019	WB present	0/5	2/5	Unassigned	*Trichococcus* sp.	87.5	KU533824
159	0.019	WB present	2/5	4/5	Unassigned	*Abiotrophia defectiva*	87.9	JF803600
114	0.019	WB absent	2/5	1/5	f: *Oxalobacteraceae*	*Massilia* sp.	99.8	JF279920
113	0.019	WB absent	3/5	0/5	g: *Agrobacterium*	*Agrobacterium tumefaciens*	100	KU955329
1	0.030	WB present	5/5	5/5	g: *Aerococcus*	*Aerococcus viridans*	95.1	KC699123
105	0.035	WB present	4/5	5/5	g: *Aerococcus*	*Aerococcus sanguinicola*	93.0	LC145565
250	0.038	WB present	1/5	2/5	o: PeHg47	*Hippea* sp.	79.5	FR754504
90	0.038	WB present	1/5	2/5	f: *Coriobacteriaceae*	*Olsenella scatoligenes*	97.8	NR_134781

* *P* value are from negative binomial Wald test, adjusted using the false discovery rate calculation described by Benjamini and Hochberg [32]

^#^ OTU was detected with significantly higher normalised read counts in koalas with (WB present) or without (WB absent) wet bottom

^+^ Classification to order (o), family (f) or genus (g) level based on comparision to Greengenes database

^^^ Organism with the lowest e-value detected using a MegaBLAST [34] search of the NCBI nucleotide database, the nucleotide identity compared to the representative sequence, and the accession number of the hit

## Discussion

Previous assessment of the koala microbiome has focused on the digestive system of koalas comparing either two free ranging animals from northern populations [35] or two captive koalas in Europe [36], from which the ocular microbiome was also assessed. Recently, the urogenital microbiomes of Queensland koalas both positive and negative for *C*. *pecorum* was assessed using 16S rRNA sequencing [9]. Our study aimed to investigate the urogenital tract microbiome of *C*. *pecorum*-free female koalas that were either positive or negative for wet bottom, in order to identify other bacteria that may be associated with this clinical sign of disease.

The most common family within the classified OTUs, in terms of either relative or normalised read abundance, was *Aerococcaceae*. Within the *Aerococcaceae*, the genera *Aerococcus* and *Facklamia* were both represented in the top four most abundant OTUs. The *Aerococcus* were also the most common genus amongst those OTUs with significant differential abundance after normalisation using DESeq2. In total, four significantly differentially abundant *Aerococcus* spp. OTUs were detected (OTU IDs 1, 2, 4, and 105). For all these OTUs, the same OTU could be detected in at least 4/5 (80%) of the converse sample group in absolute reads. For example, OTU 4 occurred in all ten koala samples, but after normalisation, was present in significantly higher (*P* = 0.004) quantities in clinically normal koalas, compared to koalas with wet bottom. Future investigations into the clinical significance of specific *Aerococcus* spp. that are either over or under-represented are needed to understand their potential role in facilitating or preventing wet-bottom.

The other family of interest are the *Tissierellaceae*, within the order *Clostridiales*. The four *Tissierellaceae* OTUs with a significant differential abundance (OTU IDs 21, 38, 47 and 51) all occurred in higher normalised quantities in koalas with wet bottom present. Three of these OTUs were in the genus *Peptoniphilus*. Interestingly, only one of these four OTUs was detected in the group of koalas without wet bottom, and only from the reads of one koala within this group. The *Peptoniphilus*, previously part of the genus *Peptostreptococcus* [37] within the family *Peptostreptococcaceae* have been associated with inflammatory diseases in other species. This includes mastitis in cattle [38] and pelvic inflammatory disease in humans [39]. Organisms in this genus are obligate anaerobes [37] and therefore potentially overlooked in traditional culture based methods of investigating urogenital tract pathogens. OTUs classified within these two genera (*Aerococcus* and *Peptoniphilus*) have also been associated with reproductive tract disease in Queensland koalas [9], however in those cases they also correlated with a high burden of *C*. *pecorum* infection, which was absent in our study.

The median number of OTUs detected in the samples used in our study is similar to the only other publication investigating the urogenital microbiome of the female koala [9]. Vidgen et al [9] analysed 155 female urogenital samples from a population of koalas in Queensland, utilising the same sequencing approach as our study. However, whilst the median number of merged reads per sample (7,652, range: 1,819–27,373) was substantially lower than our study (214,057.5, range: 183,126–300,448), the richness and diversity in their samples was marginally greater, with a median 83 (range: 17–196) OTUs detected per sample (calculated from the published data), and a median Shannon’s index of diversity of 2.96 (range: 0.14–4.98), compared to the metrics in our study of 77.95 mean richness and 2.45 mean diversity across all samples. The majority of reads in our study were classified in the order *Lactobacillales* (72.1%). This dominance of *Firmicutes* mirrors what has been seen in the urogenital tract of koalas in Queensland [9] and also the human vaginal microbiome [40]

Comparisons of the beta-diversity between the wet bottom present and absent groups highlighted that the makeup of the communities was significantly different when assessing both Bray-Curtis dissimilarity and unweighted UniFrac distances. These metrics assess the presence/absence of OTUs between groups, with UniFrac also considering phylogenetic distance between OTUs present. Weighted UniFrac distances, which consider the abundance of individual OTUs, were not significantly different between groups. Therefore, koalas with and without wet bottom appear to have a significant difference in which OTUs are present in the samples, but not necessarily in the abundance of OTUs between samples. Two samples had widely different OTU profiles (koala 49 and 70), which most likely influenced this result. This finding may support the hypothesis that wet bottom in female koalas without *C*. *pecorum* can be caused by more than one aetiological agent [6, 41]. Further investigations to examine this hypothesis are indicated but require access to a large number of appropriately collected and stored samples. Such sample sets are currently not available for this species, particularly from regions with a sufficiently low prevalence of *C*. *pecorum* to minimise any potential confounding effects.

The sample size utilised in this study is substantially smaller than many studies in human medicine, which can include hundreds of samples [42, 43], but is still larger than most published marsupial microbiome studies [35, 36, 44]. Whilst a small sample size can limit the power of a study, small sample sizes typically result in false negatives (or a type 2 error). Therefore, although this study is likely to have underestimated the total number of statistically significant OTUs, there is a high degree of confidence that the OTUs that were identified represent true differences. The samples utilised were opportunistically collected during population management exercises, and chosen from the available sample archive due to the absence of *C*. *pecorum* from the French Island koala population at the time of testing [7]. Whilst *C*. *pecorum* was subsequently determined to be present in this population [13], no koalas used in this project were positive via a *Chlamydiaceae* PCR. Importantly, no koalas used in this study were found to have reads classified within the *Chlamydiae* phylum after taxonomic assignment of OTUs, which supports the use of the 16SG PCR as a sensitive screening technique to detect *Chlamydiaceae* in clinical samples.

## Concluding remarks

We have shown, even using a relatively small sample size in our study, that koalas with wet bottom have differentially abundant OTUs in their urogenital tract compared to clinically normal koalas. Future studies with both a greater sample size, and with samples collected at multiple time points from koalas without *C*. *pecorum* but with clinical disease, would assist in understanding the pathogenesis of wet bottom in *Chlamydia*-free koalas.

## Supporting information

S1 Table Absolute abundance of merged reads clustered to assigned operational taxonomic units (OTUs). (DOCX)

S2 Table DESeq2 normalised abundance of merged reads clustered to assigned operational taxonomic units (OTUs). (DOCX)

S3 Table Statistical values of differential abundance comparisons between DESeq2 normalised reads in koalas with (K31–K70) and without (K1–K5) wet bottom (WB). (DOCX)
