# Supplementary material for: Variation in the microbiome of the urogenital tract of Chlamydia-free female koalas (Phascolarctos cinereus) with and without ‘wet bottom’
Source: PLoS One. 2018 Mar 26;13(3):e0194881. doi: 10.1371/journal.pone.0194881 (PMC5868818; doi:10.1371/journal.pone.0194881)
Supplement: S1 Table — (DOCX) [file pone.0194881.s001.docx]

**S1 Table**

**. Absolute abundance of merged reads clustered to assigned operational taxonomic units (OTUs).**

| **OTU ID** | **K1** | **K2** | **K3** | **K4** | **K5** | **K31** | **K49** | **K55** | **K59** | **K70** | **Phylum** | **Class** | **Order** | **Family** | **Genus** | **Species** |
| --- | --- | --- | --- | --- | --- | --- | --- | --- | --- | --- | --- | --- | --- | --- | --- | --- |
| OTU 1 | 23420 | 18380 | 13250 | 44770 | 1821 | 76350 | 65180 | 131700 | 124500 | 4724 | *Firmicutes* | *Bacilli* | *Lactobacillales* | *Aerococcaceae* | *Aerococcus* | *-^^^*- |
| OTU 2 | 90660 | 65200 | 60810 | 43370 | 130100 | 3915 | 713 | 955 | 28070 | 19680 | *Firmicutes* | *Bacilli* | *Lactobacillales* | *Aerococcaceae* | *Aerococcus* | *-* |
| OTU 3 | 23 | 188 | 1382 | 6 | 249 | 79 | 100900 | 3 | 9 | 55 | *Proteobacteria* | *Gammaproteobacteria* | *Enterobacteriales* | *Enterobacteriaceae* | *-* | *-* |
| OTU 4 | 22860 | 23020 | 30080 | 13500 | 27720 | 122 | 69 | 10 | 1109 | 5671 | *Firmicutes* | *Bacilli* | *Lactobacillales* | *Aerococcaceae* | *Aerococcus* | *-* |
| OTU 5 | 756 | 67 | 3121 | 2115 | 230 | 0 | 32 | 4 | 884 | 62000 | *Bacteroidetes* | *Bacteroidia* | *Bacteroidales* | *Porphyromonadaceae* | *Parabacteroides* | *-* |
| OTU 6 | 1525 | 1115 | 11920 | 34530 | 538 | 23360 | 2491 | 2467 | 1104 | 1078 | Unassigned | *-* | *-* | *-* | *-* | *-* |
| OTU 7 | 10010 | 13720 | 9683 | 9116 | 73 | 6158 | 323 | 4228 | 16 | 338 | *Firmicutes* | *Clostridia* | *Clostridiales* | *Clostridiaceae* | *Clostridium* | *-* |
| OTU 8 | 176 | 0 | 284 | 162 | 60 | 3 | 3 | 1 | 97 | 28550 | *Bacteroidetes* | *Bacteroidia* | *Bacteroidales* | *Porphyromonadaceae* | *Parabacteroides* | *-* |
| OTU 9 | 11110 | 11350 | 2591 | 0 | 1 | 1506 | 109 | 2225 | 6 | 410 | *Actinobacteria* | *Coriobacteriia* | *Coriobacteriales* | *Coriobacteriaceae* | *-* | *-* |
| OTU 10 | 16210 | 10310 | 8742 | 29050 | 519 | 26610 | 3010 | 15940 | 1950 | 1673 | *Firmicutes* | *Bacilli* | *Lactobacillales* | *Aerococcaceae* | *Facklamia* | *-* |
| OTU 11 | 316 | 24 | 0 | 1040 | 37 | 1 | 11 | 0 | 639 | 26710 | *Bacteroidetes* | *Bacteroidia* | *Bacteroidales* | *Porphyromonadaceae* | *Parabacteroides* | *-* |
| OTU 12 | 48 | 26 | 959 | 1220 | 46 | 0 | 1 | 1 | 61 | 18010 | *Firmicutes* | *Clostridia* | *Clostridiales* | *Lachnospiraceae* | *-* | *-* |
| OTU 13 | 56 | 17 | 266 | 224 | 13 | 0 | 0 | 4 | 462 | 9511 | *Firmicutes* | *Clostridia* | *Clostridiales* | *Lachnospiraceae* | *-* | *-* |
| OTU 14 | 472 | 4330 | 1134 | 0 | 0 | 3320 | 1063 | 129 | 521 | 54 | *Actinobacteria* | *Actinobacteria* | *Actinomycetales* | *Corynebacteriaceae* | *Corynebacterium* | *-* |
| OTU 15 | 42 | 886 | 358 | 0 | 5 | 2243 | 89 | 6177 | 2 | 41 | *TM7* | *TM7-3* | *I025* | *Rs-045* | *-* | *-* |
| OTU 16 | 3 | 1 | 0 | 1 | 0 | 525 | 41 | 19030 | 185 | 44 | *Bacteroidetes* | *Bacteroidia* | *Bacteroidales* | *Porphyromonadaceae* | *Porphyromonas* | *-* |
| OTU 17 | 0 | 0 | 0 | 0 | 0 | 0 | 0 | 4 | 134 | 8132 | *Firmicutes* | *Clostridia* | *Clostridiales* | *Ruminococcaceae* | *-* | *-* |
| OTU 18 | 65 | 3 | 78 | 220 | 0 | 0 | 1 | 0 | 36 | 13100 | *Firmicutes* | *Clostridia* | *Clostridiales* | *Veillonellaceae* | *Phascolarctobacterium* | *-* |
| OTU 19 | 46100 | 29040 | 21090 | 15720 | 4699 | 16980 | 2219 | 18590 | 22570 | 3193 | *Firmicutes* | *Bacilli* | *Lactobacillales* | *Aerococcaceae* | *Aerococcus* | *-* |
| OTU 20 | 181 | 34 | 507 | 625 | 20 | 0 | 0 | 1 | 18 | 10660 | *Synergistetes* | *Synergistia* | *Synergistales* | *Synergistaceae* | *-* | *-* |
| OTU 21 | 1 | 0 | 0 | 0 | 0 | 1 | 268 | 2074 | 1185 | 8 | *Firmicutes* | *Clostridia* | *Clostridiales* | *[Tissierellaceae]* | *Peptoniphilus* | *-* |
| OTU 22 | 0 | 13 | 15 | 74 | 0 | 0 | 1 | 0 | 0 | 3770 | *Firmicutes* | *Clostridia* | *Clostridiales* | *Lachnospiraceae* | *-* | *-* |
| OTU 23 | 1 | 0 | 0 | 58 | 0 | 0 | 2 | 1 | 68 | 4108 | *Bacteroidetes* | *Bacteroidia* | *Bacteroidales* | *Bacteroidaceae* | *Bacteroides* | *-* |
| OTU 24 | 53 | 6 | 0 | 4 | 0 | 0 | 1 | 0 | 4454 | 45 | *Proteobacteria* | *Gammaproteobacteria* | *Enterobacteriales* | *Enterobacteriaceae* | *-* | *-* |
| OTU 25 | 0 | 0 | 0 | 0 | 0 | 1 | 0 | 2761 | 0 | 0 | *Firmicutes* | *Clostridia* | *Clostridiales* | *Clostridiaceae* | *Clostridium* | *-* |
| OTU 26 | 691 | 511 | 1141 | 215 | 1 | 445 | 4 | 142 | 39 | 15 | *Actinobacteria* | *Actinobacteria* | *Actinomycetales* | *Propionibacteriaceae* | *-* | *-* |
| OTU 27 | 0 | 0 | 21 | 0 | 0 | 4 | 0 | 1 | 0 | 2103 | *Firmicutes* | *Clostridia* | *Clostridiales* | *Lachnospiraceae* | *-* | *-* |
| OTU 28 | 0 | 7 | 0 | 55 | 13 | 0 | 5 | 0 | 25 | 2898 | *Firmicutes* | *Clostridia* | *Clostridiales* | *Clostridiaceae* | *Clostridium* | *-* |
| OTU 29 | 1 | 0 | 0 | 0 | 0 | 0 | 195 | 90 | 2284 | 29 | *Firmicutes* | *Clostridia* | *Clostridiales* | *[Tissierellaceae]* | *Gallicola* | *-* |
| OTU 30 | 1 | 0 | 0 | 0 | 0 | 0 | 0 | 1 | 0 | 2293 | *Bacteroidetes* | *Bacteroidia* | *Bacteroidales* | *Porphyromonadaceae* | *Dysgonomonas* | *-* |
| OTU 31 | 0 | 0 | 16 | 0 | 0 | 0 | 0 | 0 | 2 | 1696 | *Firmicutes* | *Clostridia* | *Clostridiales* | *Ruminococcaceae* | *-* | *-* |
| OTU 32 | 36 | 4 | 7 | 151 | 1 | 0 | 0 | 0 | 36 | 2767 | *Fusobacteria* | *Fusobacteriia* | *Fusobacteriales* | *Fusobacteriaceae* | *Fusobacterium* | *-* |
| OTU 33 | 85 | 3 | 276 | 131 | 21 | 0 | 11 | 0 | 62 | 1414 | *Proteobacteria* | *Deltaproteobacteria* | *Desulfovibrionales* | *Desulfovibrionaceae* | *Desulfovibrio* | *-* |
| OTU 34 | 3 | 1 | 13 | 0 | 30 | 1 | 0 | 0 | 25 | 1764 | *Verrucomicrobia* | *Verrucomicrobiae* | *Verrucomicrobiales* | *Verrucomicrobiaceae* | *Akkermansia* | *muciniphila* |
| OTU 35 | 0 | 0 | 0 | 10 | 0 | 0 | 0 | 1577 | 0 | 0 | *Firmicutes* | *Clostridia* | *Clostridiales* | *Clostridiaceae* | *Clostridium* | *-* |
| OTU 36 | 5 | 0 | 477 | 45 | 0 | 0 | 0 | 1 | 1 | 3139 | *Proteobacteria* | *Gammaproteobacteria* | *Pasteurellales* | *Pasteurellaceae* | *Lonepinella* | *koalarum* |
| OTU 37 | 73 | 25 | 203 | 710 | 121 | 53 | 13 | 0 | 8 | 175 | *Proteobacteria* | *Alphaproteobacteria* | *Rhizobiales* | *Methylobacteriaceae* | *Methylobacterium* | *-* |
| OTU 38 | 0 | 0 | 0 | 0 | 0 | 46 | 130 | 396 | 913 | 16 | *Firmicutes* | *Clostridia* | *Clostridiales* | *[Tissierellaceae]* | *Peptoniphilus* | *-* |
| OTU 39 | 71 | 3 | 27 | 574 | 3 | 0 | 0 | 0 | 40 | 1191 | *Firmicutes* | *Clostridia* | *Clostridiales* | *Ruminococcaceae* | *Ruminococcus* | *-* |
| OTU 40 | 4 | 1 | 0 | 0 | 0 | 0 | 0 | 0 | 0 | 2102 | *Bacteroidetes* | *Bacteroidia* | *Bacteroidales* | *Bacteroidaceae* | *Bacteroides* | *-* |
| OTU 41 | 46 | 0 | 60 | 315 | 1 | 0 | 2 | 0 | 1 | 1875 | *Firmicutes* | *Clostridia* | *Clostridiales* | *Ruminococcaceae* | *-* | *-* |
| OTU 42 | 0 | 0 | 0 | 0 | 0 | 0 | 1 | 2454 | 0 | 1 | *Firmicutes* | *Clostridia* | *Clostridiales* | *Peptococcaceae* | *Peptococcus* | *-* |
| OTU 43 | 0 | 0 | 0 | 0 | 17 | 0 | 0 | 0 | 0 | 1748 | *Planctomycetes* | *vadinHA49* | *PeHg47* | *-* | *-* | *-* |
| OTU 44 | 0 | 1 | 30 | 0 | 0 | 0 | 0 | 0 | 6 | 1134 | *Bacteroidetes* | *Bacteroidia* | *Bacteroidales* | *Porphyromonadaceae* | *Parabacteroides* | *-* |
| OTU 45 | 139 | 27 | 180 | 1414 | 162 | 59 | 29 | 2 | 10 | 344 | *Proteobacteria* | *Alphaproteobacteria* | *Rhizobiales* | *Methylobacteriaceae* | *Methylobacterium* | *-* |
| OTU 46 | 16 | 2 | 21 | 0 | 7 | 0 | 0 | 0 | 1 | 794 | *Proteobacteria* | *Deltaproteobacteria* | *Desulfovibrionales* | *Desulfovibrionaceae* | *-* | *-* |
| OTU 47 | 0 | 0 | 0 | 0 | 0 | 0 | 31 | 418 | 130 | 0 | *Firmicutes* | *Clostridia* | *Clostridiales* | *[Tissierellaceae]* | *ph2* | *-* |
| OTU 48 | 0 | 0 | 0 | 0 | 0 | 0 | 0 | 1 | 30 | 412 | *Synergistetes* | *Synergistia* | *Synergistales* | *Synergistaceae* | *vadinCA02* | *-* |
| OTU 49 | 0 | 0 | 0 | 0 | 0 | 0 | 0 | 1 | 0 | 630 | *Firmicutes* | *Clostridia* | *Clostridiales* | *-* | *-* | *-* |
| OTU 50 | 0 | 0 | 0 | 0 | 0 | 0 | 0 | 547 | 0 | 0 | *Actinobacteria* | *Actinobacteria* | *Actinomycetales* | *Actinomycetaceae* | *Mobiluncus* | *-* |
| OTU 51 | 0 | 0 | 0 | 0 | 0 | 0 | 14 | 169 | 149 | 0 | *Firmicutes* | *Clostridia* | *Clostridiales* | *[Tissierellaceae]* | *Peptoniphilus* | *-* |
| OTU 52 | 0 | 0 | 0 | 0 | 0 | 0 | 0 | 430 | 0 | 0 | *Firmicutes* | *Clostridia* | *Clostridiales* | *Veillonellaceae* | *Dialister* | *-* |
| OTU 53 | 0 | 0 | 0 | 0 | 0 | 0 | 0 | 386 | 0 | 0 | *Firmicutes* | *Clostridia* | *Clostridiales* | *[Tissierellaceae]* | *ph2* | *-* |
| OTU 54 | 0 | 0 | 0 | 0 | 0 | 0 | 0 | 0 | 1 | 308 | *Bacteroidetes* | *Bacteroidia* | *Bacteroidales* | *Bacteroidaceae* | *Bacteroides* | *-* |
| OTU 55 | 0 | 0 | 0 | 0 | 0 | 0 | 0 | 261 | 0 | 0 | *Bacteroidetes* | *Bacteroidia* | *Bacteroidales* | *Porphyromonadaceae* | *Porphyromonas* | *-* |
| OTU 56 | 12 | 0 | 0 | 187 | 0 | 0 | 1 | 0 | 0 | 69 | *Firmicutes* | *Clostridia* | *Clostridiales* | *-* | *-* | *-* |
| OTU 57 | 15 | 0 | 34 | 92 | 9 | 2 | 0 | 0 | 2 | 22 | *Proteobacteria* | *Alphaproteobacteria* | *Rhizobiales* | *Methylobacteriaceae* | *-* | *-* |
| OTU 58 | 0 | 0 | 0 | 0 | 0 | 0 | 0 | 218 | 0 | 0 | *Firmicutes* | *Clostridia* | *Clostridiales* | *Clostridiaceae* | *Clostridium* | *-* |
| OTU 59 | 31 | 0 | 0 | 19 | 0 | 0 | 3 | 0 | 0 | 230 | *Firmicutes* | *Clostridia* | *Clostridiales* | *Lachnospiraceae* | *-* | *-* |
| OTU 60 | 0 | 0 | 0 | 318 | 0 | 0 | 0 | 0 | 0 | 0 | *Firmicutes* | *Bacilli* | *Lactobacillales* | *Streptococcaceae* | *Streptococcus* | *-* |
| OTU 61 | 8 | 0 | 1 | 136 | 10 | 0 | 3 | 0 | 0 | 0 | *Firmicutes* | *Clostridia* | *Clostridiales* | *Ruminococcaceae* | *-* | *-* |
| OTU 62 | 25 | 4 | 30 | 35 | 0 | 86 | 0 | 0 | 0 | 29 | *Proteobacteria* | *Gammaproteobacteria* | *Pseudomonadales* | *Moraxellaceae* | *Acinetobacter* | *rhizosphaerae* |
| OTU 63 | 37 | 0 | 0 | 255 | 1 | 0 | 0 | 0 | 0 | 63 | *Firmicutes* | *Clostridia* | *Clostridiales* | *Ruminococcaceae* | *-* | *-* |
| OTU 64 | 0 | 0 | 0 | 0 | 0 | 0 | 2 | 0 | 0 | 229 | *Firmicutes* | *Clostridia* | *Clostridiales* | *Ruminococcaceae* | *-* | *-* |
| OTU 65 | 0 | 0 | 0 | 0 | 1 | 60 | 0 | 407 | 0 | 0 | *Proteobacteria* | *Betaproteobacteria* | *Burkholderiales* | *Alcaligenaceae* | *Sutterella* | *-* |
| OTU 66 | 0 | 0 | 0 | 0 | 0 | 0 | 0 | 129 | 0 | 0 | *Firmicutes* | *Clostridia* | *Clostridiales* | *[Tissierellaceae]* | *ph2* | *-* |
| OTU 67 | 0 | 0 | 0 | 0 | 0 | 0 | 0 | 266 | 0 | 0 | *Bacteroidetes* | *Bacteroidia* | *Bacteroidales* | *Prevotellaceae* | *Prevotella* | *-* |
| OTU 69 | 41 | 0 | 0 | 89 | 0 | 0 | 0 | 0 | 0 | 0 | *Firmicutes* | *Clostridia* | *Clostridiales* | *Lachnospiraceae* | *-* | *-* |
| OTU 70 | 0 | 0 | 0 | 0 | 0 | 0 | 0 | 0 | 0 | 116 | *Firmicutes* | *Clostridia* | *Clostridiales* | *Ruminococcaceae* | *Oscillospira* | *-* |
| OTU 71 | 25 | 0 | 0 | 127 | 0 | 0 | 0 | 0 | 0 | 0 | *Firmicutes* | *Clostridia* | *Clostridiales* | *-* | *-* | *-* |
| OTU 72 | 0 | 0 | 0 | 0 | 0 | 0 | 0 | 0 | 0 | 156 | *Firmicutes* | *Clostridia* | *Clostridiales* | *Ruminococcaceae* | *-* | *-* |
| OTU 73 | 0 | 0 | 0 | 0 | 0 | 0 | 0 | 0 | 16 | 143 | *Bacteroidetes* | *Bacteroidia* | *Bacteroidales* | *Rikenellaceae* | *-* | *-* |
| OTU 74 | 0 | 0 | 0 | 88 | 0 | 0 | 0 | 0 | 0 | 0 | *Firmicutes* | *Clostridia* | *Clostridiales* | *Ruminococcaceae* | *-* | *-* |
| OTU 75 | 31 | 0 | 0 | 166 | 0 | 0 | 0 | 0 | 0 | 0 | *Firmicutes* | *Clostridia* | *Clostridiales* | *Lachnospiraceae* | *-* | *-* |
| OTU 76 | 7 | 0 | 19 | 79 | 1 | 0 | 1 | 1 | 0 | 35 | *Proteobacteria* | *Gammaproteobacteria* | *Pseudomonadales* | *Pseudomonadaceae* | *Pseudomonas* | *-* |
| OTU 77 | 7 | 2 | 10 | 24 | 42 | 10 | 6 | 0 | 0 | 63 | *Proteobacteria* | *Alphaproteobacteria* | *Sphingomonadales* | *Sphingomonadaceae* | *Sphingomonas* | *yabuuchiae* |
| OTU 78 | 2 | 1 | 9 | 165 | 6 | 0 | 1 | 2 | 1 | 1 | *Firmicutes* | *Bacilli* | *Bacillales* | *Staphylococcaceae* | *Staphylococcus* | *-* |
| OTU 79 | 6 | 0 | 0 | 0 | 0 | 0 | 0 | 0 | 2 | 94 | *Proteobacteria* | *Deltaproteobacteria* | *Desulfovibrionales* | *Desulfovibrionaceae* | *-* | *-* |
| OTU 80 | 0 | 0 | 0 | 79 | 0 | 0 | 0 | 0 | 0 | 0 | *Proteobacteria* | *Alphaproteobacteria* | *Rhizobiales* | *Hyphomicrobiaceae* | *Pedomicrobium* | *-* |
| OTU 81 | 0 | 0 | 0 | 0 | 0 | 0 | 0 | 0 | 0 | 108 | *Proteobacteria* | *Betaproteobacteria* | *Burkholderiales* | *Oxalobacteraceae* | *-* | *-* |
| OTU 82 | 0 | 0 | 0 | 0 | 0 | 0 | 0 | 0 | 0 | 95 | *Proteobacteria* | *Deltaproteobacteria* | *Desulfovibrionales* | *Desulfovibrionaceae* | *-* | *-* |
| OTU 83 | 0 | 1 | 0 | 0 | 0 | 0 | 0 | 0 | 1 | 98 | *Bacteroidetes* | *Bacteroidia* | *Bacteroidales* | *Bacteroidaceae* | *Bacteroides* | *fragilis* |
| OTU 84 | 0 | 0 | 80 | 0 | 0 | 0 | 0 | 0 | 0 | 0 | *Firmicutes* | *Clostridia* | *Clostridiales* | *-* | *-* | *-* |
| OTU 86 | 29 | 0 | 73 | 43 | 0 | 0 | 0 | 0 | 0 | 0 | *Bacteroidetes* | *Bacteroidia* | *Bacteroidales* | *Bacteroidaceae* | *Bacteroides* | *-* |
| OTU 87 | 0 | 0 | 0 | 67 | 0 | 0 | 0 | 0 | 0 | 0 | *Firmicutes* | *Clostridia* | *Clostridiales* | *Lachnospiraceae* | *-* | *-* |
| OTU 88 | 0 | 70 | 0 | 0 | 0 | 0 | 0 | 0 | 0 | 0 | *Firmicutes* | *Clostridia* | *Clostridiales* | *Ruminococcaceae* | *-* | *-* |
| OTU 89 | 0 | 0 | 0 | 46 | 0 | 0 | 0 | 0 | 0 | 0 | *Firmicutes* | *Clostridia* | *Clostridiales* | *-* | *-* | *-* |
| OTU 90 | 10 | 0 | 0 | 0 | 0 | 32 | 0 | 118 | 0 | 0 | *Actinobacteria* | *Coriobacteriia* | *Coriobacteriales* | *Coriobacteriaceae* | *-* | *-* |
| OTU 91 | 0 | 0 | 0 | 0 | 0 | 0 | 0 | 0 | 0 | 153 | *Proteobacteria* | *Deltaproteobacteria* | *Desulfarculales* | *Desulfarculaceae* | *-* | *-* |
| OTU 92 | 5 | 0 | 11 | 50 | 0 | 0 | 0 | 0 | 2 | 17 | *Proteobacteria* | *Betaproteobacteria* | *Burkholderiales* | *Comamonadaceae* | *Comamonas* | *-* |
| OTU 93 | 0 | 0 | 0 | 0 | 0 | 0 | 0 | 0 | 1 | 144 | *Bacteroidetes* | *Bacteroidia* | *Bacteroidales* | *Bacteroidaceae* | *Bacteroides* | *-* |
| OTU 94 | 0 | 0 | 0 | 131 | 14 | 0 | 0 | 1 | 0 | 0 | *Proteobacteria* | *Alphaproteobacteria* | *Rhizobiales* | *Methylocystaceae* | *-* | *-* |
| OTU 95 | 0 | 0 | 18 | 39 | 0 | 0 | 0 | 0 | 0 | 0 | *Proteobacteria* | *Alphaproteobacteria* | *Rhizobiales* | *Rhizobiaceae* | *Rhizobium* | *leguminosarum* |
| OTU 96 | 0 | 1 | 0 | 239 | 0 | 0 | 0 | 0 | 1 | 0 | *Firmicutes* | *Clostridia* | *Clostridiales* | *-* | *-* | *-* |
| OTU 97 | 1 | 2 | 4 | 55 | 3 | 0 | 0 | 0 | 1 | 17 | *Proteobacteria* | *Betaproteobacteria* | *Burkholderiales* | *Comamonadaceae* | *-* | *-* |
| OTU 98 | 0 | 0 | 0 | 0 | 0 | 0 | 0 | 0 | 1 | 140 | *Planctomycetes* | *vadinHA49* | *PeHg47* | *-* | *-* | *-* |
| OTU 99 | 0 | 0 | 0 | 0 | 0 | 0 | 0 | 71 | 0 | 0 | *Actinobacteria* | *Coriobacteriia* | *Coriobacteriales* | *Coriobacteriaceae* | *-* | *-* |
| OTU 100 | 0 | 0 | 0 | 0 | 0 | 0 | 0 | 64 | 0 | 0 | *Firmicutes* | *Clostridia* | *Clostridiales* | *[Mogibacteriaceae]* | *Mogibacterium* | *-* |
| OTU 101 | 0 | 0 | 2 | 12 | 11 | 6 | 2 | 0 | 0 | 17 | *Bacteroidetes* | *[Saprospirae]* | *[Saprospirales]* | *Chitinophagaceae* | *Sediminibacterium* | *-* |
| OTU 102 | 0 | 0 | 1 | 52 | 2 | 2 | 1 | 0 | 0 | 0 | *Firmicutes* | *Clostridia* | *Clostridiales* | *Lachnospiraceae* | *Blautia* | *producta* |
| OTU 103 | 14 | 0 | 45 | 0 | 0 | 0 | 0 | 0 | 0 | 0 | Unassigned | *-* | *-* | *-* | *-* | *-* |
| OTU 105 | 27 | 46 | 18 | 30 | 0 | 122 | 342 | 403 | 99 | 11 | *Firmicutes* | *Bacilli* | *Lactobacillales* | *Aerococcaceae* | *Aerococcus* | *-* |
| OTU 106 | 2 | 0 | 11 | 0 | 41 | 0 | 0 | 0 | 0 | 0 | *Proteobacteria* | *Betaproteobacteria* | *Burkholderiales* | *Burkholderiaceae* | *Burkholderia* | *-* |
| OTU 107 | 0 | 0 | 0 | 63 | 0 | 0 | 0 | 0 | 0 | 0 | *Proteobacteria* | *Alphaproteobacteria* | *Sphingomonadales* | *Sphingomonadaceae* | *Sphingobium* | *-* |
| OTU 108 | 0 | 0 | 0 | 34 | 0 | 3 | 0 | 0 | 0 | 0 | *Firmicutes* | *Clostridia* | *Clostridiales* | *-* | *-* | *-* |
| OTU 109 | 0 | 0 | 0 | 0 | 0 | 0 | 36 | 15 | 0 | 0 | *Firmicutes* | *Clostridia* | *Clostridiales* | *Peptostreptococcaceae* | *Peptostreptococcus* | *-* |
| OTU 110 | 0 | 0 | 0 | 0 | 0 | 0 | 0 | 0 | 0 | 27 | *Acidobacteria* | *Acidobacteriia* | *Acidobacteriales* | *Acidobacteriaceae* | *-* | *-* |
| OTU 111 | 0 | 3 | 0 | 0 | 0 | 0 | 1 | 0 | 1 | 37 | *Firmicutes* | *Clostridia* | *Clostridiales* | *Ruminococcaceae* | *-* | *-* |
| OTU 112 | 2 | 0 | 0 | 30 | 0 | 0 | 0 | 0 | 0 | 0 | *Actinobacteria* | *Actinobacteria* | *Actinomycetales* | *Nocardiaceae* | *Rhodococcus* | *fascians* |
| OTU 113 | 0 | 0 | 5 | 14 | 9 | 0 | 0 | 0 | 0 | 0 | *Proteobacteria* | *Alphaproteobacteria* | *Rhizobiales* | *Rhizobiaceae* | *Agrobacterium* | *-* |
| OTU 114 | 0 | 0 | 0 | 40 | 10 | 0 | 0 | 0 | 1 | 0 | *Proteobacteria* | *Betaproteobacteria* | *Burkholderiales* | *Oxalobacteraceae* | *-* | *-* |
| OTU 115 | 3 | 0 | 7 | 0 | 0 | 0 | 0 | 0 | 0 | 43 | *Cyanobacteria* | *4C0d-2* | *YS2* | *-* | *-* | *-* |
| OTU 116 | 0 | 0 | 0 | 0 | 0 | 0 | 0 | 0 | 0 | 39 | *Firmicutes* | *Clostridia* | *Clostridiales* | *Ruminococcaceae* | *-* | *-* |
| OTU 117 | 0 | 0 | 0 | 0 | 0 | 0 | 0 | 0 | 0 | 32 | *Proteobacteria* | *Deltaproteobacteria* | *Desulfobacterales* | *Desulfobacteraceae* | *-* | *-* |
| OTU 118 | 0 | 0 | 0 | 0 | 0 | 0 | 0 | 0 | 0 | 36 | *Bacteroidetes* | *Bacteroidia* | *Bacteroidales* | *Porphyromonadaceae* | *Parabacteroides* | *-* |
| OTU 119 | 2 | 0 | 0 | 22 | 0 | 0 | 0 | 0 | 0 | 7 | *Proteobacteria* | *Betaproteobacteria* | *Burkholderiales* | *Oxalobacteraceae* | *Ralstonia* | *-* |
| OTU 120 | 0 | 0 | 46 | 0 | 0 | 0 | 0 | 0 | 0 | 0 | *Bacteroidetes* | *Bacteroidia* | *Bacteroidales* | *[Paraprevotellaceae]* | *Paraprevotella* | *-* |
| OTU 121 | 0 | 0 | 0 | 37 | 0 | 0 | 0 | 0 | 0 | 0 | *Firmicutes* | *Erysipelotrichi* | *Erysipelotrichales* | *Erysipelotrichaceae* | *Coprobacillus* | *-* |
| OTU 122 | 0 | 0 | 0 | 59 | 0 | 0 | 0 | 0 | 0 | 0 | *Actinobacteria* | *Actinobacteria* | *Actinomycetales* | *Corynebacteriaceae* | *Corynebacterium* | *-* |
| OTU 123 | 0 | 0 | 0 | 6 | 13 | 1 | 17 | 1 | 5 | 92 | Unassigned | *-* | *-* | *-* | *-* | *-* |
| OTU 124 | 33 | 0 | 0 | 0 | 0 | 0 | 0 | 0 | 0 | 0 | *Actinobacteria* | *Actinobacteria* | *Actinomycetales* | *Micrococcaceae* | *Rothia* | *mucilaginosa* |
| OTU 125 | 0 | 0 | 54 | 0 | 0 | 0 | 0 | 0 | 0 | 0 | *Proteobacteria* | *Deltaproteobacteria* | *Desulfarculales* | *Desulfarculaceae* | *-* | *-* |
| OTU 126 | 0 | 0 | 0 | 45 | 1 | 1 | 0 | 0 | 0 | 0 | *Firmicutes* | *Clostridia* | *Clostridiales* | *Lachnospiraceae* | *-* | *-* |
| OTU 127 | 0 | 0 | 0 | 0 | 0 | 2 | 0 | 0 | 0 | 25 | *Firmicutes* | *Clostridia* | *Clostridiales* | *Lachnospiraceae* | *-* | *-* |
| OTU 128 | 0 | 0 | 0 | 0 | 0 | 0 | 0 | 0 | 0 | 53 | *Planctomycetes* | *vadinHA49* | *PeHg47* | *-* | *-* | *-* |
| OTU 129 | 0 | 0 | 14 | 0 | 0 | 0 | 0 | 0 | 0 | 0 | *Actinobacteria* | *Actinobacteria* | *Actinomycetales* | *Micrococcaceae* | *-* | *-* |
| OTU 130 | 0 | 0 | 1 | 26 | 0 | 0 | 1 | 0 | 0 | 0 | *Bacteroidetes* | *Bacteroidia* | *Bacteroidales* | *Bacteroidaceae* | *Bacteroides* | *-* |
| OTU 131 | 0 | 0 | 15 | 0 | 17 | 2 | 1 | 0 | 0 | 0 | *Firmicutes* | *Clostridia* | *Clostridiales* | *-* | *-* | *-* |
| OTU 132 | 4 | 1 | 31 | 0 | 0 | 4 | 1 | 0 | 0 | 16 | *Proteobacteria* | *Betaproteobacteria* | *Burkholderiales* | *Comamonadaceae* | *-* | *-* |
| OTU 133 | 0 | 0 | 23 | 0 | 0 | 0 | 0 | 0 | 0 | 0 | *Bacteroidetes* | *Cytophagia* | *Cytophagales* | *Cytophagaceae* | *Hymenobacter* | *-* |
| OTU 134 | 0 | 0 | 0 | 25 | 0 | 0 | 0 | 0 | 0 | 0 | *Firmicutes* | *Clostridia* | *Clostridiales* | *Eubacteriaceae* | *Anaerofustis* | *-* |
| OTU 135 | 0 | 0 | 0 | 0 | 0 | 0 | 0 | 0 | 18 | 0 | *Bacteroidetes* | *Bacteroidia* | *Bacteroidales* | *Bacteroidaceae* | *Bacteroides* | *uniformis* |
| OTU 136 | 0 | 0 | 0 | 20 | 0 | 3 | 0 | 0 | 0 | 0 | *Bacteroidetes* | *Bacteroidia* | *Bacteroidales* | *Bacteroidaceae* | *Bacteroides* | *-* |
| OTU 137 | 13 | 0 | 19 | 0 | 7 | 0 | 0 | 0 | 0 | 17 | *Actinobacteria* | *Actinobacteria* | *Actinomycetales* | *Corynebacteriaceae* | *Corynebacterium* | *-* |
| OTU 138 | 0 | 17 | 0 | 0 | 0 | 0 | 0 | 0 | 0 | 0 | *Firmicutes* | *Clostridia* | *Clostridiales* | *Ruminococcaceae* | *Oscillospira* | *-* |
| OTU 139 | 0 | 0 | 0 | 18 | 0 | 0 | 0 | 0 | 0 | 0 | *Actinobacteria* | *Actinobacteria* | *Actinomycetales* | *Micrococcaceae* | *Rothia* | *dentocariosa* |
| OTU 140 | 11 | 0 | 0 | 0 | 0 | 0 | 0 | 0 | 2 | 14 | *Proteobacteria* | *Gammaproteobacteria* | *Pseudomonadales* | *Moraxellaceae* | *Acinetobacter* | *-* |
| OTU 141 | 0 | 0 | 0 | 21 | 3 | 2 | 0 | 0 | 0 | 0 | *Actinobacteria* | *Thermoleophilia* | *Solirubrobacterales* | *-* | *-* | *-* |
| OTU 142 | 0 | 0 | 0 | 0 | 0 | 0 | 28 | 0 | 0 | 0 | *Firmicutes* | *Clostridia* | *Clostridiales* | *-* | *-* | *-* |
| OTU 143 | 0 | 0 | 0 | 13 | 0 | 0 | 0 | 0 | 0 | 0 | *Bacteroidetes* | *Bacteroidia* | *Bacteroidales* | *Bacteroidaceae* | *Bacteroides* | *ovatus* |
| OTU 144 | 5 | 0 | 0 | 0 | 23 | 0 | 0 | 0 | 0 | 0 | *Actinobacteria* | *Actinobacteria* | *Actinomycetales* | *Corynebacteriaceae* | *Corynebacterium* | *-* |
| OTU 145 | 0 | 0 | 0 | 0 | 0 | 0 | 0 | 0 | 0 | 17 | *Firmicutes* | *Clostridia* | *Clostridiales* | *Clostridiaceae* | *Clostridium* | *-* |
| OTU 146 | 8 | 0 | 19 | 0 | 0 | 0 | 0 | 0 | 0 | 0 | *Bacteroidetes* | *Bacteroidia* | *Bacteroidales* | *Prevotellaceae* | *Prevotella* | *-* |
| OTU 147 | 0 | 0 | 0 | 0 | 0 | 0 | 0 | 0 | 0 | 23 | Unassigned | *-* | *-* | *-* | *-* | *-* |
| OTU 148 | 0 | 0 | 0 | 0 | 0 | 0 | 0 | 31 | 21 | 0 | Unassigned | *-* | *-* | *-* | *-* | *-* |
| OTU 149 | 0 | 0 | 8 | 0 | 0 | 0 | 0 | 0 | 0 | 8 | *Proteobacteria* | *Deltaproteobacteria* | *Desulfovibrionales* | *Desulfovibrionaceae* | *Desulfovibrio* | *-* |
| OTU 150 | 0 | 0 | 34 | 0 | 0 | 0 | 0 | 0 | 0 | 0 | *Bacteroidetes* | *Cytophagia* | *Cytophagales* | *Cytophagaceae* | *Hymenobacter* | *-* |
| OTU 151 | 0 | 0 | 23 | 0 | 3 | 0 | 0 | 0 | 0 | 0 | *Actinobacteria* | *Actinobacteria* | *Actinomycetales* | *Kineosporiaceae* | *-* | *-* |
| OTU 152 | 0 | 0 | 0 | 0 | 0 | 0 | 0 | 0 | 0 | 31 | *Proteobacteria* | *Deltaproteobacteria* | *Desulfovibrionales* | *Desulfovibrionaceae* | *-* | *-* |
| OTU 153 | 3 | 1 | 0 | 0 | 0 | 0 | 0 | 0 | 0 | 17 | *Proteobacteria* | *Deltaproteobacteria* | *Desulfovibrionales* | *Desulfovibrionaceae* | *Bilophila* | *-* |
| OTU 154 | 0 | 0 | 14 | 0 | 0 | 0 | 0 | 0 | 0 | 0 | *Proteobacteria* | *Deltaproteobacteria* | *Desulfovibrionales* | *Desulfovibrionaceae* | *-* | *-* |
| OTU 155 | 34 | 104 | 14 | 15 | 0 | 103 | 13 | 440 | 0 | 0 | *Firmicutes* | *Bacilli* | *Lactobacillales* | *Carnobacteriaceae* | *Trichococcus* | *-* |
| OTU 156 | 5 | 1 | 0 | 0 | 11 | 0 | 0 | 0 | 0 | 0 | *Armatimonadetes* | *[Fimbriimonadia]* | *[Fimbriimonadales]* | *[Fimbriimonadaceae]* | *Fimbriimonas* | *-* |
| OTU 157 | 4 | 0 | 0 | 0 | 0 | 7 | 3 | 0 | 2 | 1 | *Firmicutes* | *Clostridia* | *Clostridiales* | *Clostridiaceae* | *Clostridium* | *-* |
| OTU 158 | 0 | 0 | 0 | 0 | 11 | 0 | 0 | 0 | 0 | 0 | *Proteobacteria* | *Alphaproteobacteria* | *Rhizobiales* | *Methylocystaceae* | *-* | *-* |
| OTU 159 | 0 | 1 | 3 | 0 | 0 | 5 | 1 | 44 | 19 | 0 | Unassigned | *-* | *-* | *-* | *-* | *-* |
| OTU 160 | 0 | 1 | 0 | 0 | 0 | 0 | 0 | 0 | 0 | 13 | *Firmicutes* | *Clostridia* | *Clostridiales* | *-* | *-* | *-* |
| OTU 161 | 0 | 0 | 1 | 0 | 18 | 2 | 0 | 0 | 1 | 0 | *Firmicutes* | *Clostridia* | *Clostridiales* | *Clostridiaceae* | *Clostridium* | *perfringens* |
| OTU 162 | 0 | 1 | 0 | 12 | 0 | 0 | 0 | 0 | 0 | 0 | *Firmicutes* | *Clostridia* | *Clostridiales* | *Ruminococcaceae* | *-* | *-* |
| OTU 163 | 14 | 0 | 0 | 0 | 4 | 0 | 0 | 0 | 0 | 0 | *Proteobacteria* | *Gammaproteobacteria* | *Oceanospirillales* | *Halomonadaceae* | *-* | *-* |
| OTU 164 | 0 | 0 | 0 | 0 | 0 | 1 | 0 | 0 | 0 | 15 | *Proteobacteria* | *Gammaproteobacteria* | *Pseudomonadales* | *Moraxellaceae* | *Acinetobacter* | *-* |
| OTU 165 | 10 | 4 | 25 | 12 | 0 | 58 | 7 | 25 | 7 | 0 | Unassigned | *-* | *-* | *-* | *-* | *-* |
| OTU 166 | 0 | 0 | 0 | 0 | 0 | 0 | 0 | 22 | 0 | 0 | *Firmicutes* | *Clostridia* | *Clostridiales* | *Lachnospiraceae* | *-* | *-* |
| OTU 167 | 0 | 0 | 0 | 0 | 0 | 0 | 2 | 0 | 0 | 20 | *Firmicutes* | *Clostridia* | *Clostridiales* | *Lachnospiraceae* | *Dorea* | *-* |
| OTU 168 | 0 | 0 | 0 | 13 | 0 | 0 | 0 | 0 | 0 | 0 | *Firmicutes* | *Clostridia* | *Clostridiales* | *Ruminococcaceae* | *Oscillospira* | *-* |
| OTU 169 | 0 | 0 | 0 | 0 | 0 | 0 | 0 | 15 | 0 | 0 | *Actinobacteria* | *Actinobacteria* | *Actinomycetales* | *Intrasporangiaceae* | *Terracoccus* | *-* |
| OTU 170 | 0 | 2 | 1 | 0 | 18 | 0 | 0 | 0 | 0 | 0 | *Firmicutes* | *Bacilli* | *Lactobacillales* | *Enterococcaceae* | *-* | *-* |
| OTU 171 | 2 | 0 | 0 | 8 | 0 | 0 | 0 | 0 | 0 | 0 | *Firmicutes* | *Clostridia* | *Clostridiales* | *Lachnospiraceae* | *Blautia* | *-* |
| OTU 172 | 4 | 0 | 3 | 7 | 13 | 0 | 2 | 0 | 0 | 0 | *Proteobacteria* | *Betaproteobacteria* | *Burkholderiales* | *Oxalobacteraceae* | *Ralstonia* | *-* |
| OTU 173 | 0 | 0 | 0 | 0 | 0 | 0 | 0 | 0 | 0 | 11 | *Deferribacteres* | *Deferribacteres* | *Deferribacterales* | *Deferribacteraceae* | *Mucispirillum* | *-* |
| OTU 174 | 0 | 0 | 0 | 0 | 11 | 2 | 0 | 0 | 0 | 23 | Unassigned | *-* | *-* | *-* | *-* | *-* |
| OTU 175 | 5 | 0 | 0 | 19 | 0 | 0 | 0 | 0 | 0 | 0 | *Proteobacteria* | *Betaproteobacteria* | *Burkholderiales* | *Oxalobacteraceae* | *Oxalobacter* | *formigenes* |
| OTU 176 | 0 | 0 | 0 | 0 | 0 | 0 | 0 | 0 | 0 | 9 | *Firmicutes* | *Clostridia* | *Clostridiales* | *Lachnospiraceae* | *-* | *-* |
| OTU 177 | 0 | 0 | 0 | 0 | 0 | 0 | 0 | 0 | 0 | 27 | *Proteobacteria* | *Deltaproteobacteria* | *Desulfovibrionales* | *Desulfovibrionaceae* | *Bilophila* | *-* |
| OTU 178 | 0 | 1 | 0 | 0 | 0 | 0 | 0 | 0 | 0 | 15 | Unassigned | *-* | *-* | *-* | *-* | *-* |
| OTU 179 | 6 | 0 | 1 | 0 | 0 | 0 | 0 | 0 | 0 | 0 | *Actinobacteria* | *Actinobacteria* | *Actinomycetales* | *Corynebacteriaceae* | *Corynebacterium* | *-* |
| OTU 180 | 7 | 0 | 0 | 0 | 0 | 0 | 0 | 0 | 0 | 0 | *Proteobacteria* | *Alphaproteobacteria* | *Rhodospirillales* | *Rhodospirillaceae* | *-* | *-* |
| OTU 181 | 0 | 0 | 0 | 0 | 0 | 0 | 0 | 0 | 0 | 48 | *Bacteroidetes* | *Bacteroidia* | *Bacteroidales* | *Porphyromonadaceae* | *Parabacteroides* | *-* |
| OTU 182 | 0 | 0 | 7 | 0 | 0 | 0 | 0 | 0 | 0 | 0 | *Cyanobacteria* | *4C0d-2* | *MLE1-12* | *-* | *-* | *-* |
| OTU 183 | 1 | 5 | 0 | 1 | 0 | 0 | 0 | 1 | 3 | 63 | *Proteobacteria* | *Gammaproteobacteria* | *Pseudomonadales* | *Pseudomonadaceae* | *Pseudomonas* | *-* |
| OTU 184 | 6 | 0 | 0 | 0 | 9 | 0 | 0 | 0 | 0 | 5 | *Proteobacteria* | *Betaproteobacteria* | *Burkholderiales* | *Comamonadaceae* | *-* | *-* |
| OTU 185 | 0 | 0 | 6 | 0 | 0 | 0 | 0 | 0 | 0 | 0 | *Proteobacteria* | *Alphaproteobacteria* | *Rhodospirillales* | *Acetobacteraceae* | *-* | *-* |
| OTU 186 | 0 | 0 | 0 | 0 | 0 | 0 | 0 | 0 | 6 | 0 | *Firmicutes* | *Clostridia* | *Clostridiales* | *[Tissierellaceae]* | *Peptoniphilus* | *-* |
| OTU 187 | 4 | 0 | 0 | 0 | 5 | 0 | 0 | 0 | 0 | 8 | *Proteobacteria* | *Gammaproteobacteria* | *Pseudomonadales* | *Moraxellaceae* | *Enhydrobacter* | *-* |
| OTU 188 | 6 | 0 | 0 | 0 | 0 | 0 | 0 | 0 | 0 | 0 | *Proteobacteria* | *Betaproteobacteria* | *Neisseriales* | *Neisseriaceae* | *Neisseria* | *subflava* |
| OTU 189 | 0 | 0 | 0 | 0 | 5 | 0 | 0 | 0 | 0 | 0 | *Proteobacteria* | *Alphaproteobacteria* | *Rhodospirillales* | *Acetobacteraceae* | *-* | *-* |
| OTU 190 | 0 | 0 | 0 | 0 | 6 | 0 | 0 | 0 | 0 | 0 | *Actinobacteria* | *Actinobacteria* | *Actinomycetales* | *Nocardioidaceae* | *-* | *-* |
| OTU 191 | 0 | 0 | 0 | 0 | 0 | 0 | 0 | 0 | 0 | 39 | *Planctomycetes* | *vadinHA49* | *PeHg47* | *-* | *-* | *-* |
| OTU 192 | 0 | 0 | 0 | 0 | 0 | 0 | 0 | 0 | 0 | 11 | *Bacteroidetes* | *Bacteroidia* | *Bacteroidales* | *Bacteroidaceae* | *Bacteroides* | *ovatus* |
| OTU 193 | 0 | 0 | 9 | 13 | 0 | 0 | 0 | 0 | 0 | 0 | *Firmicutes* | *Clostridia* | *Clostridiales* | *Lachnospiraceae* | *-* | *-* |
| OTU 194 | 0 | 0 | 0 | 0 | 0 | 0 | 0 | 0 | 0 | 25 | *Bacteroidetes* | *Bacteroidia* | *Bacteroidales* | *Rikenellaceae* | *-* | *-* |
| OTU 195 | 18 | 0 | 0 | 0 | 0 | 0 | 0 | 0 | 0 | 0 | *Firmicutes* | *Clostridia* | *Clostridiales* | *Ruminococcaceae* | *-* | *-* |
| OTU 196 | 0 | 1 | 1 | 3 | 4 | 5 | 3 | 1 | 1 | 1 | *Proteobacteria* | *Gammaproteobacteria* | *Enterobacteriales* | *Enterobacteriaceae* | *Plesiomonas* | *shigelloides* |
| OTU 197 | 0 | 0 | 0 | 0 | 4 | 0 | 0 | 0 | 0 | 0 | *Acidobacteria* | *Acidobacteriia* | *Acidobacteriales* | *Acidobacteriaceae* | *Terriglobus* | *-* |
| OTU 199 | 0 | 0 | 9 | 0 | 0 | 0 | 0 | 0 | 0 | 0 | *Actinobacteria* | *Actinobacteria* | *Actinomycetales* | *Corynebacteriaceae* | *Corynebacterium* | *-* |
| OTU 200 | 2 | 18 | 0 | 0 | 0 | 7 | 0 | 66 | 0 | 0 | Unassigned | *-* | *-* | *-* | *-* | *-* |
| OTU 201 | 0 | 0 | 0 | 0 | 0 | 0 | 0 | 79 | 0 | 0 | *Firmicutes* | *Bacilli* | *Lactobacillales* | *Aerococcaceae* | *Aerococcus* | *-* |
| OTU 202 | 0 | 0 | 0 | 0 | 0 | 0 | 0 | 0 | 0 | 12 | *Firmicutes* | *Clostridia* | *Clostridiales* | *Lachnospiraceae* | *Coprococcus* | *-* |
| OTU 204 | 4 | 0 | 0 | 1 | 4 | 2 | 0 | 2 | 0 | 1 | *Fusobacteria* | *Fusobacteriia* | *Fusobacteriales* | *Leptotrichiaceae* | *-* | *-* |
| OTU 205 | 0 | 2 | 0 | 0 | 0 | 0 | 0 | 0 | 0 | 3 | *Firmicutes* | *Clostridia* | *Clostridiales* | *Lachnospiraceae* | *-* | *-* |
| OTU 206 | 0 | 0 | 0 | 0 | 0 | 0 | 0 | 0 | 0 | 16 | *Proteobacteria* | *Gammaproteobacteria* | *Pasteurellales* | *Pasteurellaceae* | *Aggregatibacter* | *-* |
| OTU 207 | 0 | 0 | 0 | 0 | 0 | 0 | 0 | 0 | 0 | 6 | Unassigned | *-* | *-* | *-* | *-* | *-* |
| OTU 209 | 0 | 1 | 2 | 1 | 4 | 2 | 1 | 1 | 1 | 0 | *Firmicutes* | *Clostridia* | *Clostridiales* | *Peptostreptococcaceae* | *-* | *-* |
| OTU 210 | 0 | 0 | 0 | 0 | 0 | 0 | 0 | 0 | 0 | 8 | *Bacteroidetes* | *Bacteroidia* | *Bacteroidales* | *Rikenellaceae* | *-* | *-* |
| OTU 212 | 0 | 0 | 0 | 9 | 0 | 0 | 0 | 0 | 0 | 0 | *Firmicutes* | *Clostridia* | *Clostridiales* | *Dehalobacteriaceae* | *-* | *-* |
| OTU 213 | 0 | 19 | 0 | 0 | 1 | 0 | 0 | 0 | 0 | 19 | *Firmicutes* | *Clostridia* | *Clostridiales* | *-* | *-* | *-* |
| OTU 214 | 0 | 0 | 0 | 0 | 0 | 0 | 0 | 0 | 0 | 6 | *Proteobacteria* | *Gammaproteobacteria* | *Pseudomonadales* | *Moraxellaceae* | *Acinetobacter* | *-* |
| OTU 215 | 7 | 0 | 0 | 5 | 0 | 0 | 0 | 0 | 0 | 0 | *Firmicutes* | *Clostridia* | *Clostridiales* | *Veillonellaceae* | *Veillonella* | *dispar* |
| OTU 216 | 0 | 0 | 0 | 0 | 0 | 0 | 0 | 20 | 0 | 0 | Unassigned | *-* | *-* | *-* | *-* | *-* |
| OTU 217 | 7 | 0 | 0 | 0 | 0 | 0 | 0 | 0 | 0 | 0 | *Proteobacteria* | *Alphaproteobacteria* | *Rhizobiales* | *Bradyrhizobiaceae* | *-* | *-* |
| OTU 218 | 0 | 0 | 0 | 0 | 6 | 0 | 2 | 0 | 2 | 0 | *Firmicutes* | *Bacilli* | *Bacillales* | *-* | *-* | *-* |
| OTU 219 | 0 | 0 | 0 | 0 | 0 | 5 | 0 | 0 | 0 | 0 | *Proteobacteria* | *Alphaproteobacteria* | *Rhodobacterales* | *Rhodobacteraceae* | *Amaricoccus* | *-* |
| OTU 220 | 0 | 8 | 0 | 1 | 0 | 0 | 0 | 0 | 0 | 45 | *Firmicutes* | *Clostridia* | *Clostridiales* | *Lachnospiraceae* | *-* | *-* |
| OTU 221 | 0 | 0 | 0 | 0 | 0 | 0 | 0 | 0 | 0 | 12 | Unassigned | *-* | *-* | *-* | *-* | *-* |
| OTU 222 | 1 | 10 | 1 | 0 | 0 | 0 | 0 | 0 | 0 | 0 | Unassigned | *-* | *-* | *-* | *-* | *-* |
| OTU 223 | 12 | 0 | 0 | 0 | 5 | 0 | 0 | 3 | 0 | 0 | *Firmicutes* | *Bacilli* | *Lactobacillales* | *Streptococcaceae* | *Streptococcus* | *-* |
| OTU 224 | 0 | 1 | 0 | 0 | 0 | 7 | 0 | 0 | 0 | 0 | *Proteobacteria* | *Alphaproteobacteria* | *Sphingomonadales* | *Sphingomonadaceae* | *-* | *-* |
| OTU 225 | 0 | 0 | 0 | 0 | 4 | 0 | 0 | 0 | 0 | 0 | *Proteobacteria* | *Betaproteobacteria* | *Gallionellales* | *Gallionellaceae* | *Gallionella* | *-* |
| OTU 226 | 0 | 0 | 0 | 0 | 0 | 0 | 0 | 10 | 0 | 0 | Unassigned | *-* | *-* | *-* | *-* | *-* |
| OTU 227 | 0 | 0 | 0 | 0 | 0 | 0 | 0 | 492 | 0 | 1 | Unassigned | *-* | *-* | *-* | *-* | *-* |
| OTU 228 | 5 | 0 | 0 | 0 | 0 | 0 | 0 | 0 | 0 | 0 | *Firmicutes* | *Clostridia* | *Clostridiales* | *Ruminococcaceae* | *Ruminococcus* | *-* |
| OTU 229 | 0 | 5 | 0 | 4 | 0 | 5 | 0 | 0 | 0 | 0 | *Firmicutes* | *Clostridia* | *Clostridiales* | *Clostridiaceae* | *-* | *-* |
| OTU 230 | 0 | 0 | 0 | 0 | 1 | 0 | 0 | 0 | 0 | 3 | Unassigned | *-* | *-* | *-* | *-* | *-* |
| OTU 231 | 0 | 0 | 9 | 0 | 5 | 0 | 0 | 0 | 0 | 0 | *Proteobacteria* | *Alphaproteobacteria* | *Rhizobiales* | *Methylocystaceae* | *-* | *-* |
| OTU 232 | 0 | 0 | 0 | 0 | 0 | 0 | 0 | 0 | 2 | 0 | *Proteobacteria* | *Alphaproteobacteria* | *Rhodospirillales* | *Acetobacteraceae* | *-* | *-* |
| OTU 233 | 0 | 0 | 0 | 0 | 2 | 0 | 0 | 0 | 0 | 2 | Unassigned | *-* | *-* | *-* | *-* | *-* |
| OTU 234 | 0 | 0 | 0 | 0 | 4 | 0 | 0 | 0 | 0 | 0 | *Bacteroidetes* | *Bacteroidia* | *Bacteroidales* | *Bacteroidaceae* | *Bacteroides* | *-* |
| OTU 235 | 0 | 0 | 0 | 0 | 6 | 0 | 0 | 0 | 0 | 0 | *Bacteroidetes* | *Bacteroidia* | *Bacteroidales* | *Bacteroidaceae* | *Bacteroides* | *-* |
| OTU 236 | 0 | 0 | 0 | 0 | 0 | 0 | 0 | 0 | 0 | 2 | *Bacteroidetes* | *Bacteroidia* | *Bacteroidales* | *Rikenellaceae* | *-* | *-* |
| OTU 237 | 0 | 0 | 0 | 0 | 0 | 4 | 0 | 0 | 0 | 0 | *Bacteroidetes* | *Bacteroidia* | *Bacteroidales* | *Bacteroidaceae* | *Bacteroides* | *-* |
| OTU 238 | 0 | 0 | 0 | 0 | 0 | 0 | 0 | 121 | 0 | 0 | Unassigned | *-* | *-* | *-* | *-* | *-* |
| OTU 239 | 0 | 3 | 2 | 0 | 0 | 0 | 0 | 0 | 2 | 0 | *Fusobacteria* | *Fusobacteriia* | *Fusobacteriales* | *Fusobacteriaceae* | *Fusobacterium* | *-* |
| OTU 240 | 0 | 0 | 0 | 0 | 0 | 0 | 0 | 0 | 4 | 750 | *Bacteroidetes* | *Bacteroidia* | *Bacteroidales* | *Porphyromonadaceae* | *Parabacteroides* | *-* |
| OTU 241 | 6 | 0 | 0 | 0 | 0 | 0 | 0 | 0 | 0 | 0 | *Firmicutes* | *Clostridia* | *Clostridiales* | *[Tissierellaceae]* | *Anaerococcus* | *-* |
| OTU 242 | 1 | 0 | 6 | 5 | 0 | 0 | 0 | 0 | 0 | 0 | *Proteobacteria* | *Betaproteobacteria* | *Burkholderiales* | *Comamonadaceae* | *-* | *-* |
| OTU 243 | 1 | 2 | 2 | 0 | 5 | 1 | 0 | 1 | 1 | 0 | *Fusobacteria* | *Fusobacteriia* | *Fusobacteriales* | *Fusobacteriaceae* | *Cetobacterium* | *somerae* |
| OTU 244 | 0 | 0 | 0 | 0 | 0 | 0 | 0 | 0 | 0 | 17 | *Firmicutes* | *Clostridia* | *Clostridiales* | *Ruminococcaceae* | *-* | *-* |
| OTU 245 | 0 | 1 | 2 | 0 | 0 | 0 | 0 | 0 | 0 | 4 | *Synergistetes* | *Synergistia* | *Synergistales* | *Synergistaceae* | *-* | *-* |
| OTU 246 | 0 | 0 | 8 | 0 | 0 | 0 | 0 | 0 | 0 | 0 | *Actinobacteria* | *Coriobacteriia* | *Coriobacteriales* | *Coriobacteriaceae* | *-* | *-* |
| OTU 247 | 0 | 0 | 0 | 0 | 0 | 0 | 0 | 0 | 0 | 7 | *Bacteroidetes* | *Bacteroidia* | *Bacteroidales* | *Rikenellaceae* | *-* | *-* |
| OTU 248 | 3 | 0 | 0 | 0 | 0 | 0 | 0 | 0 | 0 | 0 | *Firmicutes* | *Clostridia* | *Clostridiales* | *Lachnospiraceae* | *-* | *-* |
| OTU 249 | 0 | 6 | 0 | 0 | 0 | 1 | 1 | 5 | 7 | 0 | Unassigned | *-* | *-* | *-* | *-* | *-* |
| OTU 250 | 0 | 0 | 2 | 0 | 0 | 0 | 0 | 0 | 9 | 47 | *Planctomycetes* | *vadinHA49* | *PeHg47* | *-* | *-* | *-* |
| OTU 251 | 0 | 0 | 0 | 40 | 0 | 0 | 0 | 0 | 0 | 0 | *Firmicutes* | *Clostridia* | *Clostridiales* | *-* | *-* | *-* |
| OTU 252 | 0 | 0 | 0 | 0 | 0 | 0 | 0 | 0 | 0 | 19 | *Firmicutes* | *Clostridia* | *Clostridiales* | *Veillonellaceae* | *-* | *-* |
| OTU 253 | 0 | 0 | 0 | 0 | 0 | 0 | 0 | 2 | 0 | 0 | Unassigned | *-* | *-* | *-* | *-* | *-* |
| OTU 254 | 1 | 0 | 8 | 0 | 0 | 0 | 0 | 0 | 0 | 0 | *Actinobacteria* | *Actinobacteria* | *Actinomycetales* | *Actinomycetaceae* | *Actinomyces* | *-* |
| OTU 255 | 0 | 0 | 0 | 0 | 0 | 0 | 0 | 0 | 0 | 5 | *Proteobacteria* | *Deltaproteobacteria* | *Desulfovibrionales* | *Desulfovibrionaceae* | *-* | *-* |
| OTU 256 | 0 | 0 | 0 | 0 | 5 | 0 | 1 | 0 | 0 | 0 | *Proteobacteria* | *Alphaproteobacteria* | *Rhodospirillales* | *Rhodospirillaceae* | *-* | *-* |
| OTU 257 | 0 | 0 | 0 | 0 | 0 | 0 | 0 | 3 | 0 | 0 | Unassigned | *-* | *-* | *-* | *-* | *-* |
| OTU 258 | 1 | 0 | 1 | 0 | 0 | 0 | 7 | 0 | 0 | 0 | *Firmicutes* | *Clostridia* | *Clostridiales* | *Lachnospiraceae* | *Roseburia* | *-* |
| OTU 259 | 0 | 6 | 0 | 0 | 0 | 0 | 0 | 1 | 0 | 0 | *Firmicutes* | *Clostridia* | *Clostridiales* | *Ruminococcaceae* | *-* | *-* |
| OTU 260 | 0 | 0 | 4 | 0 | 0 | 0 | 0 | 0 | 3 | 0 | *Actinobacteria* | *Actinobacteria* | *Actinomycetales* | *Micrococcaceae* | *Kocuria* | *palustris* |
| OTU 261 | 0 | 0 | 0 | 0 | 0 | 0 | 0 | 7 | 16 | 0 | Unassigned | *-* | *-* | *-* | *-* | *-* |

OTUs were assigned a taxonomy (with 90% identity) from the Greengenes curated database [1]. Koalas K1 – K5 were clinically normal (wet bottom absent), whilst koalas K31 – K70 had wet bottom.

^^^ Classification could not be made to this level

1. DeSantis TZ, Hugenholtz P, Larsen N, Rojas M, Brodie EL, Keller K, et al. Greengenes, a chimera-checked 16S rRNA gene database and workbench compatible with ARB. Appl Environ Microbiol. 2006;72(7):5069-72. doi: 10.1128/aem.03006-05.
