# Supplementary material for: Variation in the microbiome of the urogenital tract of Chlamydia-free female koalas (Phascolarctos cinereus) with and without ‘wet bottom’
Source: PLoS One. 2018 Mar 26;13(3):e0194881. doi: 10.1371/journal.pone.0194881 (PMC5868818; doi:10.1371/journal.pone.0194881)
Supplement: S2 Table — (DOCX) [file pone.0194881.s002.docx]

**S2 Table. DESeq2 normalised abundance of merged reads clustered to assigned operational taxonomic units (OTUs).**

| **OTU ID** | **K1** | **K2** | **K3** | **K4** | **K5** | **K31** | **K49** | **K55** | **K59** | **K70** | **Phylum** | **Class** | **Order** | **Family** | **Genus** | **Species** |
| --- | --- | --- | --- | --- | --- | --- | --- | --- | --- | --- | --- | --- | --- | --- | --- | --- |
| OTU 1 | 14.6 | 14.4 | 13.7 | 15.8 | 11.4 | 17.1 | 16.8 | 18.4 | 18.3 | 12.2 | *Firmicutes* | *Bacilli* | *Lactobacillales* | *Aerococcaceae* | *Aerococcus* | *-^^^* |
| OTU 2 | 17.3 | 16.8 | 16.4 | 15.7 | 18.4 | 12.2 | 10.5 | 10.7 | 15.0 | 14.2 | *Firmicutes* | *Bacilli* | *Lactobacillales* | *Aerococcaceae* | *Aerococcus* | *-* |
| OTU 3 | 7.0 | 9.2 | 11.0 | 5.4 | 9.5 | 8.4 | 17.9 | 4.7 | 6.0 | 7.8 | *Proteobacteria* | *Gammaproteobacteria* | *Enterobacteriales* | *Enterobacteriaceae* | *-* | *-* |
| OTU 4 | 14.6 | 14.8 | 15.0 | 13.7 | 15.0 | 8.8 | 8.3 | 6.1 | 10.8 | 12.5 | *Firmicutes* | *Bacilli* | *Lactobacillales* | *Aerococcaceae* | *Aerococcus* | *-* |
| OTU 5 | 10.4 | 8.2 | 11.9 | 11.4 | 9.4 | 1.9 | 7.5 | 5.0 | 10.6 | 16.3 | *Bacteroidetes* | *Bacteroidia* | *Bacteroidales* | *Porphyromonadaceae* | *Parabacteroides* | *-* |
| OTU 6 | 11.1 | 10.9 | 13.6 | 15.3 | 10.2 | 14.7 | 11.7 | 11.7 | 10.8 | 10.7 | Unassigned | *-* | *-* | *-* | *-* | *-* |
| OTU 7 | 13.3 | 13.9 | 13.3 | 13.2 | 8.3 | 12.8 | 9.7 | 12.3 | 6.6 | 9.6 | *Firmicutes* | *Clostridia* | *Clostridiales* | *Clostridiaceae* | *Clostridium* | *-* |
| OTU 8 | 9.1 | 2.0 | 9.5 | 9.0 | 8.1 | 4.7 | 4.8 | 3.4 | 8.5 | 14.8 | *Bacteroidetes* | *Bacteroidia* | *Bacteroidales* | *Porphyromonadaceae* | *Parabacteroides* | *-* |
| OTU 9 | 13.5 | 13.6 | 11.7 | 1.7 | 3.4 | 11.2 | 8.7 | 11.5 | 5.5 | 9.8 | *Actinobacteria* | *Coriobacteriia* | *Coriobacteriales* | *Coriobacteriaceae* | *-* | *-* |
| OTU 10 | 14.0 | 13.5 | 13.1 | 14.9 | 10.1 | 15.0 | 12.0 | 14.1 | 11.4 | 11.1 | *Firmicutes* | *Bacilli* | *Lactobacillales* | *Aerococcaceae* | *Facklamia* | *-* |
| OTU 11 | 9.6 | 7.1 | 1.8 | 10.7 | 7.6 | 3.5 | 6.3 | 1.9 | 10.3 | 14.7 | *Bacteroidetes* | *Bacteroidia* | *Bacteroidales* | *Porphyromonadaceae* | *Parabacteroides* | *-* |
| OTU 12 | 7.8 | 7.2 | 10.6 | 10.9 | 7.8 | 1.9 | 3.5 | 3.4 | 8.1 | 14.0 | *Firmicutes* | *Clostridia* | *Clostridiales* | *Lachnospiraceae* | *-* | *-* |
| OTU 13 | 7.9 | 6.8 | 9.4 | 9.3 | 6.4 | 1.9 | 2.0 | 5.0 | 10.0 | 13.1 | *Firmicutes* | *Clostridia* | *Clostridiales* | *Lachnospiraceae* | *-* | *-* |
| OTU 14 | 10.0 | 12.4 | 10.8 | 1.7 | 1.9 | 12.0 | 10.9 | 8.8 | 10.1 | 7.8 | *Actinobacteria* | *Actinobacteria* | *Actinomycetales* | *Corynebacteriaceae* | *Corynebacterium* | *-* |
| OTU 15 | 7.6 | 10.7 | 9.7 | 1.7 | 5.3 | 11.6 | 8.5 | 12.7 | 4.2 | 7.5 | *TM7* | *TM7-3* | *I025* | *Rs-045* | *-* | *-* |
| OTU 16 | 4.6 | 3.5 | 1.8 | 3.2 | 1.9 | 10.1 | 7.7 | 14.4 | 9.2 | 7.6 | *Bacteroidetes* | *Bacteroidia* | *Bacteroidales* | *Porphyromonadaceae* | *Porphyromonas* | *-* |
| OTU 17 | 1.8 | 2.0 | 1.8 | 1.7 | 1.9 | 1.9 | 2.0 | 5.0 | 8.9 | 12.9 | *Firmicutes* | *Clostridia* | *Clostridiales* | *Ruminococcaceae* | *-* | *-* |
| OTU 18 | 8.1 | 4.8 | 8.3 | 9.3 | 1.9 | 1.9 | 3.5 | 1.9 | 7.5 | 13.6 | *Firmicutes* | *Clostridia* | *Clostridiales* | *Veillonellaceae* | *Phascolarctobacterium* | *-* |
| OTU 19 | 15.9 | 15.2 | 14.4 | 13.9 | 12.4 | 14.2 | 11.6 | 14.3 | 14.6 | 11.8 | *Firmicutes* | *Bacilli* | *Lactobacillales* | *Aerococcaceae* | *Aerococcus* | *-* |
| OTU 20 | 9.1 | 7.5 | 10.0 | 10.2 | 6.9 | 1.9 | 2.0 | 3.4 | 6.8 | 13.3 | *Synergistetes* | *Synergistia* | *Synergistales* | *Synergistaceae* | *-* | *-* |
| OTU 21 | 3.3 | 2.0 | 1.8 | 1.7 | 1.9 | 3.5 | 9.6 | 11.5 | 10.9 | 5.6 | *Firmicutes* | *Clostridia* | *Clostridiales* | *[Tissierellaceae]* | *Peptoniphilus* | *-* |
| OTU 22 | 1.8 | 6.5 | 6.5 | 8.2 | 1.9 | 1.9 | 3.5 | 1.9 | 1.9 | 12.0 | *Firmicutes* | *Clostridia* | *Clostridiales* | *Lachnospiraceae* | *-* | *-* |
| OTU 23 | 3.3 | 2.0 | 1.8 | 7.9 | 1.9 | 1.9 | 4.3 | 3.4 | 8.2 | 12.1 | *Bacteroidetes* | *Bacteroidia* | *Bacteroidales* | *Bacteroidaceae* | *Bacteroides* | *-* |
| OTU 24 | 7.9 | 5.6 | 1.8 | 4.9 | 1.9 | 1.9 | 3.5 | 1.9 | 12.3 | 7.6 | *Proteobacteria* | *Gammaproteobacteria* | *Enterobacteriales* | *Enterobacteriaceae* | *-* | *-* |
| OTU 25 | 1.8 | 2.0 | 1.8 | 1.7 | 1.9 | 3.5 | 2.0 | 11.8 | 1.9 | 1.6 | *Firmicutes* | *Clostridia* | *Clostridiales* | *Clostridiaceae* | *Clostridium* | *-* |
| OTU 26 | 10.3 | 10.1 | 10.8 | 9.2 | 3.4 | 10.0 | 5.1 | 8.9 | 7.6 | 6.4 | *Actinobacteria* | *Actinobacteria* | *Actinomycetales* | *Propionibacteriaceae* | *-* | *-* |
| OTU 27 | 1.8 | 2.0 | 6.9 | 1.7 | 1.9 | 5.1 | 2.0 | 3.4 | 1.9 | 11.3 | *Firmicutes* | *Clostridia* | *Clostridiales* | *Lachnospiraceae* | *-* | *-* |
| OTU 28 | 1.8 | 5.8 | 1.8 | 7.9 | 6.4 | 1.9 | 5.4 | 1.9 | 7.1 | 11.7 | *Firmicutes* | *Clostridia* | *Clostridiales* | *Clostridiaceae* | *Clostridium* | *-* |
| OTU 29 | 3.3 | 2.0 | 1.8 | 1.7 | 1.9 | 1.9 | 9.3 | 8.5 | 11.6 | 7.1 | *Firmicutes* | *Clostridia* | *Clostridiales* | *[Tissierellaceae]* | *Gallicola* | *-* |
| OTU 30 | 3.3 | 2.0 | 1.8 | 1.7 | 1.9 | 1.9 | 2.0 | 3.4 | 1.9 | 11.4 | *Bacteroidetes* | *Bacteroidia* | *Bacteroidales* | *Porphyromonadaceae* | *Dysgonomonas* | *-* |
| OTU 31 | 1.8 | 2.0 | 6.6 | 1.7 | 1.9 | 1.9 | 2.0 | 1.9 | 4.2 | 11.1 | *Firmicutes* | *Clostridia* | *Clostridiales* | *Ruminococcaceae* | *-* | *-* |
| OTU 32 | 7.5 | 5.1 | 5.6 | 8.9 | 3.4 | 1.9 | 2.0 | 1.9 | 7.5 | 11.6 | *Fusobacteria* | *Fusobacteriia* | *Fusobacteriales* | *Fusobacteriaceae* | *Fusobacterium* | *-* |
| OTU 33 | 8.3 | 4.8 | 9.5 | 8.8 | 7.0 | 1.9 | 6.3 | 1.9 | 8.1 | 10.9 | *Proteobacteria* | *Deltaproteobacteria* | *Desulfovibrionales* | *Desulfovibrionaceae* | *Desulfovibrio* | *-* |
| OTU 34 | 4.6 | 3.5 | 6.3 | 1.7 | 7.3 | 3.5 | 2.0 | 1.9 | 7.1 | 11.1 | *Verrucomicrobia* | *Verrucomicrobiae* | *Verrucomicrobiales* | *Verrucomicrobiaceae* | *Akkermansia* | *muciniphila* |
| OTU 35 | 1.8 | 2.0 | 1.8 | 6.0 | 1.9 | 1.9 | 2.0 | 11.2 | 1.9 | 1.6 | *Firmicutes* | *Clostridia* | *Clostridiales* | *Clostridiaceae* | *Clostridium* | *-* |
| OTU 36 | 5.2 | 2.0 | 10.0 | 7.7 | 1.9 | 1.9 | 2.0 | 3.4 | 3.4 | 11.8 | *Proteobacteria* | *Gammaproteobacteria* | *Pasteurellales* | *Pasteurellaceae* | *Lonepinella* | *koalarum* |
| OTU 37 | 8.2 | 7.2 | 9.2 | 10.3 | 8.8 | 7.9 | 6.5 | 1.9 | 5.8 | 9.0 | *Proteobacteria* | *Alphaproteobacteria* | *Rhizobiales* | *Methylobacteriaceae* | *Methylobacterium* | *-* |
| OTU 38 | 1.8 | 2.0 | 1.8 | 1.7 | 1.9 | 7.8 | 8.9 | 9.9 | 10.6 | 6.5 | *Firmicutes* | *Clostridia* | *Clostridiales* | *[Tissierellaceae]* | *Peptoniphilus* | *-* |
| OTU 39 | 8.2 | 4.8 | 7.1 | 10.1 | 4.7 | 1.9 | 2.0 | 1.9 | 7.6 | 10.8 | *Firmicutes* | *Clostridia* | *Clostridiales* | *Ruminococcaceae* | *Ruminococcus* | *-* |
| OTU 40 | 4.9 | 3.5 | 1.8 | 1.7 | 1.9 | 1.9 | 2.0 | 1.9 | 1.9 | 11.3 | *Bacteroidetes* | *Bacteroidia* | *Bacteroidales* | *Bacteroidaceae* | *Bacteroides* | *-* |
| OTU 41 | 7.7 | 2.0 | 8.0 | 9.6 | 3.4 | 1.9 | 4.3 | 1.9 | 3.4 | 11.2 | *Firmicutes* | *Clostridia* | *Clostridiales* | *Ruminococcaceae* | *-* | *-* |
| OTU 42 | 1.8 | 2.0 | 1.8 | 1.7 | 1.9 | 1.9 | 3.5 | 11.7 | 1.9 | 3.1 | *Firmicutes* | *Clostridia* | *Clostridiales* | *Peptococcaceae* | *Peptococcus* | *-* |
| OTU 43 | 1.8 | 2.0 | 1.8 | 1.7 | 6.7 | 1.9 | 2.0 | 1.9 | 1.9 | 11.1 | *Planctomycetes* | *vadinHA49* | *PeHg47* | *-* | *-* | *-* |
| OTU 44 | 1.8 | 3.5 | 7.3 | 1.7 | 1.9 | 1.9 | 2.0 | 1.9 | 5.5 | 10.7 | *Bacteroidetes* | *Bacteroidia* | *Bacteroidales* | *Porphyromonadaceae* | *Parabacteroides* | *-* |
| OTU 45 | 8.8 | 7.3 | 9.1 | 11.0 | 9.0 | 8.1 | 7.3 | 4.2 | 6.1 | 9.6 | *Proteobacteria* | *Alphaproteobacteria* | *Rhizobiales* | *Methylobacteriaceae* | *Methylobacterium* | *-* |
| OTU 46 | 6.6 | 4.3 | 6.9 | 1.7 | 5.7 | 1.9 | 2.0 | 1.9 | 3.4 | 10.4 | *Proteobacteria* | *Deltaproteobacteria* | *Desulfovibrionales* | *Desulfovibrionaceae* | *-* | *-* |
| OTU 47 | 1.8 | 2.0 | 1.8 | 1.7 | 1.9 | 1.9 | 7.4 | 9.9 | 8.8 | 1.6 | *Firmicutes* | *Clostridia* | *Clostridiales* | *[Tissierellaceae]* | *ph2* | *-* |
| OTU 48 | 1.8 | 2.0 | 1.8 | 1.7 | 1.9 | 1.9 | 2.0 | 3.4 | 7.3 | 9.8 | *Synergistetes* | *Synergistia* | *Synergistales* | *Synergistaceae* | *vadinCA02* | *-* |
| OTU 49 | 1.8 | 2.0 | 1.8 | 1.7 | 1.9 | 1.9 | 2.0 | 3.4 | 1.9 | 10.2 | *Firmicutes* | *Clostridia* | *Clostridiales* | *-* | *-* | *-* |
| OTU 50 | 1.8 | 2.0 | 1.8 | 1.7 | 1.9 | 1.9 | 2.0 | 10.2 | 1.9 | 1.6 | *Actinobacteria* | *Actinobacteria* | *Actinomycetales* | *Actinomycetaceae* | *Mobiluncus* | *-* |
| OTU 51 | 1.8 | 2.0 | 1.8 | 1.7 | 1.9 | 1.9 | 6.6 | 9.1 | 9.0 | 1.6 | *Firmicutes* | *Clostridia* | *Clostridiales* | *[Tissierellaceae]* | *Peptoniphilus* | *-* |
| OTU 52 | 1.8 | 2.0 | 1.8 | 1.7 | 1.9 | 1.9 | 2.0 | 9.9 | 1.9 | 1.6 | *Firmicutes* | *Clostridia* | *Clostridiales* | *Veillonellaceae* | *Dialister* | *-* |
| OTU 53 | 1.8 | 2.0 | 1.8 | 1.7 | 1.9 | 1.9 | 2.0 | 9.8 | 1.9 | 1.6 | *Firmicutes* | *Clostridia* | *Clostridiales* | *[Tissierellaceae]* | *ph2* | *-* |
| OTU 54 | 1.8 | 2.0 | 1.8 | 1.7 | 1.9 | 1.9 | 2.0 | 1.9 | 3.4 | 9.5 | *Bacteroidetes* | *Bacteroidia* | *Bacteroidales* | *Bacteroidaceae* | *Bacteroides* | *-* |
| OTU 55 | 1.8 | 2.0 | 1.8 | 1.7 | 1.9 | 1.9 | 2.0 | 9.5 | 1.9 | 1.6 | *Bacteroidetes* | *Bacteroidia* | *Bacteroidales* | *Porphyromonadaceae* | *Porphyromonas* | *-* |
| OTU 56 | 6.3 | 2.0 | 1.8 | 9.1 | 1.9 | 1.9 | 3.5 | 1.9 | 1.9 | 8.0 | *Firmicutes* | *Clostridia* | *Clostridiales* | *-* | *-* | *-* |
| OTU 57 | 6.5 | 2.0 | 7.4 | 8.4 | 6.0 | 4.2 | 2.0 | 1.9 | 4.2 | 6.8 | *Proteobacteria* | *Alphaproteobacteria* | *Rhizobiales* | *Methylobacteriaceae* | *-* | *-* |
| OTU 58 | 1.8 | 2.0 | 1.8 | 1.7 | 1.9 | 1.9 | 2.0 | 9.3 | 1.9 | 1.6 | *Firmicutes* | *Clostridia* | *Clostridiales* | *Clostridiaceae* | *Clostridium* | *-* |
| OTU 59 | 7.3 | 2.0 | 1.8 | 6.7 | 1.9 | 1.9 | 4.8 | 1.9 | 1.9 | 9.2 | *Firmicutes* | *Clostridia* | *Clostridiales* | *Lachnospiraceae* | *-* | *-* |
| OTU 60 | 1.8 | 2.0 | 1.8 | 9.6 | 1.9 | 1.9 | 2.0 | 1.9 | 1.9 | 1.6 | *Firmicutes* | *Bacilli* | *Lactobacillales* | *Streptococcaceae* | *Streptococcus* | *-* |
| OTU 61 | 5.8 | 2.0 | 3.3 | 8.8 | 6.1 | 1.9 | 4.8 | 1.9 | 1.9 | 1.6 | *Firmicutes* | *Clostridia* | *Clostridiales* | *Ruminococcaceae* | *-* | *-* |
| OTU 62 | 7.1 | 5.1 | 7.3 | 7.4 | 1.9 | 8.4 | 2.0 | 1.9 | 1.9 | 7.1 | *Proteobacteria* | *Gammaproteobacteria* | *Pseudomonadales* | *Moraxellaceae* | *Acinetobacter* | *rhizosphaerae* |
| OTU 63 | 7.5 | 2.0 | 1.8 | 9.4 | 3.4 | 1.9 | 2.0 | 1.9 | 1.9 | 7.9 | *Firmicutes* | *Clostridia* | *Clostridiales* | *Ruminococcaceae* | *-* | *-* |
| OTU 64 | 1.8 | 2.0 | 1.8 | 1.7 | 1.9 | 1.9 | 4.3 | 1.9 | 1.9 | 9.2 | *Firmicutes* | *Clostridia* | *Clostridiales* | *Ruminococcaceae* | *-* | *-* |
| OTU 65 | 1.8 | 2.0 | 1.8 | 1.7 | 3.4 | 8.1 | 2.0 | 9.9 | 1.9 | 1.6 | *Proteobacteria* | *Betaproteobacteria* | *Burkholderiales* | *Alcaligenaceae* | *Sutterella* | *-* |
| OTU 66 | 1.8 | 2.0 | 1.8 | 1.7 | 1.9 | 1.9 | 2.0 | 8.8 | 1.9 | 1.6 | *Firmicutes* | *Clostridia* | *Clostridiales* | *[Tissierellaceae]* | *ph2* | *-* |
| OTU 67 | 1.8 | 2.0 | 1.8 | 1.7 | 1.9 | 1.9 | 2.0 | 9.5 | 1.9 | 1.6 | *Bacteroidetes* | *Bacteroidia* | *Bacteroidales* | *Prevotellaceae* | *Prevotella* | *-* |
| OTU 69 | 7.6 | 2.0 | 1.8 | 8.4 | 1.9 | 1.9 | 2.0 | 1.9 | 1.9 | 1.6 | *Firmicutes* | *Clostridia* | *Clostridiales* | *Lachnospiraceae* | *-* | *-* |
| OTU 70 | 1.8 | 2.0 | 1.8 | 1.7 | 1.9 | 1.9 | 2.0 | 1.9 | 1.9 | 8.6 | *Firmicutes* | *Clostridia* | *Clostridiales* | *Ruminococcaceae* | *Oscillospira* | *-* |
| OTU 71 | 7.1 | 2.0 | 1.8 | 8.7 | 1.9 | 1.9 | 2.0 | 1.9 | 1.9 | 1.6 | *Firmicutes* | *Clostridia* | *Clostridiales* | *-* | *-* | *-* |
| OTU 72 | 1.8 | 2.0 | 1.8 | 1.7 | 1.9 | 1.9 | 2.0 | 1.9 | 1.9 | 8.9 | *Firmicutes* | *Clostridia* | *Clostridiales* | *Ruminococcaceae* | *-* | *-* |
| OTU 73 | 1.8 | 2.0 | 1.8 | 1.7 | 1.9 | 1.9 | 2.0 | 1.9 | 6.6 | 8.8 | *Bacteroidetes* | *Bacteroidia* | *Bacteroidales* | *Rikenellaceae* | *-* | *-* |
| OTU 74 | 1.8 | 2.0 | 1.8 | 8.4 | 1.9 | 1.9 | 2.0 | 1.9 | 1.9 | 1.6 | *Firmicutes* | *Clostridia* | *Clostridiales* | *Ruminococcaceae* | *-* | *-* |
| OTU 75 | 7.3 | 2.0 | 1.8 | 9.0 | 1.9 | 1.9 | 2.0 | 1.9 | 1.9 | 1.6 | *Firmicutes* | *Clostridia* | *Clostridiales* | *Lachnospiraceae* | *-* | *-* |
| OTU 76 | 5.6 | 2.0 | 6.8 | 8.2 | 3.4 | 1.9 | 3.5 | 3.4 | 1.9 | 7.3 | *Proteobacteria* | *Gammaproteobacteria* | *Pseudomonadales* | *Pseudomonadaceae* | *Pseudomonas* | *-* |
| OTU 77 | 5.6 | 4.3 | 6.0 | 7.0 | 7.7 | 6.1 | 5.6 | 1.9 | 1.9 | 7.9 | *Proteobacteria* | *Alphaproteobacteria* | *Sphingomonadales* | *Sphingomonadaceae* | *Sphingomonas* | *yabuuchiae* |
| OTU 78 | 4.1 | 3.5 | 5.9 | 9.0 | 5.5 | 1.9 | 3.5 | 4.2 | 3.4 | 3.1 | *Firmicutes* | *Bacilli* | *Bacillales* | *Staphylococcaceae* | *Staphylococcus* | *-* |
| OTU 79 | 5.4 | 2.0 | 1.8 | 1.7 | 1.9 | 1.9 | 2.0 | 1.9 | 4.2 | 8.3 | *Proteobacteria* | *Deltaproteobacteria* | *Desulfovibrionales* | *Desulfovibrionaceae* | *-* | *-* |
| OTU 80 | 1.8 | 2.0 | 1.8 | 8.2 | 1.9 | 1.9 | 2.0 | 1.9 | 1.9 | 1.6 | *Proteobacteria* | *Alphaproteobacteria* | *Rhizobiales* | *Hyphomicrobiaceae* | *Pedomicrobium* | *-* |
| OTU 81 | 1.8 | 2.0 | 1.8 | 1.7 | 1.9 | 1.9 | 2.0 | 1.9 | 1.9 | 8.5 | *Proteobacteria* | *Betaproteobacteria* | *Burkholderiales* | *Oxalobacteraceae* | *-* | *-* |
| OTU 82 | 1.8 | 2.0 | 1.8 | 1.7 | 1.9 | 1.9 | 2.0 | 1.9 | 1.9 | 8.4 | *Proteobacteria* | *Deltaproteobacteria* | *Desulfovibrionales* | *Desulfovibrionaceae* | *-* | *-* |
| OTU 83 | 1.8 | 3.5 | 1.8 | 1.7 | 1.9 | 1.9 | 2.0 | 1.9 | 3.4 | 8.4 | *Bacteroidetes* | *Bacteroidia* | *Bacteroidales* | *Bacteroidaceae* | *Bacteroides* | *fragilis* |
| OTU 84 | 1.8 | 2.0 | 8.3 | 1.7 | 1.9 | 1.9 | 2.0 | 1.9 | 1.9 | 1.6 | *Firmicutes* | *Clostridia* | *Clostridiales* | *-* | *-* | *-* |
| OTU 86 | 7.2 | 2.0 | 8.2 | 7.6 | 1.9 | 1.9 | 2.0 | 1.9 | 1.9 | 1.6 | *Bacteroidetes* | *Bacteroidia* | *Bacteroidales* | *Bacteroidaceae* | *Bacteroides* | *-* |
| OTU 87 | 1.8 | 2.0 | 1.8 | 8.1 | 1.9 | 1.9 | 2.0 | 1.9 | 1.9 | 1.6 | *Firmicutes* | *Clostridia* | *Clostridiales* | *Lachnospiraceae* | *-* | *-* |
| OTU 88 | 1.8 | 8.3 | 1.8 | 1.7 | 1.9 | 1.9 | 2.0 | 1.9 | 1.9 | 1.6 | *Firmicutes* | *Clostridia* | *Clostridiales* | *Ruminococcaceae* | *-* | *-* |
| OTU 89 | 1.8 | 2.0 | 1.8 | 7.7 | 1.9 | 1.9 | 2.0 | 1.9 | 1.9 | 1.6 | *Firmicutes* | *Clostridia* | *Clostridiales* | *-* | *-* | *-* |
| OTU 90 | 6.0 | 2.0 | 1.8 | 1.7 | 1.9 | 7.4 | 2.0 | 8.7 | 1.9 | 1.6 | *Actinobacteria* | *Coriobacteriia* | *Coriobacteriales* | *Coriobacteriaceae* | *-* | *-* |
| OTU 91 | 1.8 | 2.0 | 1.8 | 1.7 | 1.9 | 1.9 | 2.0 | 1.9 | 1.9 | 8.8 | *Proteobacteria* | *Deltaproteobacteria* | *Desulfarculales* | *Desulfarculaceae* | *-* | *-* |
| OTU 92 | 5.2 | 2.0 | 6.1 | 7.8 | 1.9 | 1.9 | 2.0 | 1.9 | 4.2 | 6.5 | *Proteobacteria* | *Betaproteobacteria* | *Burkholderiales* | *Comamonadaceae* | *Comamonas* | *-* |
| OTU 93 | 1.8 | 2.0 | 1.8 | 1.7 | 1.9 | 1.9 | 2.0 | 1.9 | 3.4 | 8.8 | *Bacteroidetes* | *Bacteroidia* | *Bacteroidales* | *Bacteroidaceae* | *Bacteroides* | *-* |
| OTU 94 | 1.8 | 2.0 | 1.8 | 8.8 | 6.5 | 1.9 | 2.0 | 3.4 | 1.9 | 1.6 | *Proteobacteria* | *Alphaproteobacteria* | *Rhizobiales* | *Methylocystaceae* | *-* | *-* |
| OTU 95 | 1.8 | 2.0 | 6.7 | 7.5 | 1.9 | 1.9 | 2.0 | 1.9 | 1.9 | 1.6 | *Proteobacteria* | *Alphaproteobacteria* | *Rhizobiales* | *Rhizobiaceae* | *Rhizobium* | *leguminosarum* |
| OTU 96 | 1.8 | 3.5 | 1.8 | 9.3 | 1.9 | 1.9 | 2.0 | 1.9 | 3.4 | 1.6 | *Firmicutes* | *Clostridia* | *Clostridiales* | *-* | *-* | *-* |
| OTU 97 | 3.3 | 4.3 | 4.9 | 7.9 | 4.7 | 1.9 | 2.0 | 1.9 | 3.4 | 6.5 | *Proteobacteria* | *Betaproteobacteria* | *Burkholderiales* | *Comamonadaceae* | *-* | *-* |
| OTU 98 | 1.8 | 2.0 | 1.8 | 1.7 | 1.9 | 1.9 | 2.0 | 1.9 | 3.4 | 8.7 | *Planctomycetes* | *vadinHA49* | *PeHg47* | *-* | *-* | *-* |
| OTU 99 | 1.8 | 2.0 | 1.8 | 1.7 | 1.9 | 1.9 | 2.0 | 8.2 | 1.9 | 1.6 | *Actinobacteria* | *Coriobacteriia* | *Coriobacteriales* | *Coriobacteriaceae* | *-* | *-* |
| OTU 100 | 1.8 | 2.0 | 1.8 | 1.7 | 1.9 | 1.9 | 2.0 | 8.1 | 1.9 | 1.6 | *Firmicutes* | *Clostridia* | *Clostridiales* | *[Mogibacteriaceae]* | *Mogibacterium* | *-* |
| OTU 101 | 1.8 | 2.0 | 4.1 | 6.2 | 6.2 | 5.5 | 4.3 | 1.9 | 1.9 | 6.5 | *Bacteroidetes* | *[Saprospirae]* | *[Saprospirales]* | *Chitinophagaceae* | *Sediminibacterium* | *-* |
| OTU 102 | 1.8 | 2.0 | 3.3 | 7.8 | 4.2 | 4.2 | 3.5 | 1.9 | 1.9 | 1.6 | *Firmicutes* | *Clostridia* | *Clostridiales* | *Lachnospiraceae* | *Blautia* | *producta* |
| OTU 103 | 6.4 | 2.0 | 7.7 | 1.7 | 1.9 | 1.9 | 2.0 | 1.9 | 1.9 | 1.6 | Unassigned | *-* | *-* | *-* | *-* | *-* |
| OTU 105 | 7.1 | 7.8 | 6.7 | 7.2 | 1.9 | 8.8 | 9.8 | 9.9 | 8.6 | 6.0 | *Firmicutes* | *Bacilli* | *Lactobacillales* | *Aerococcaceae* | *Aerococcus* | *-* |
| OTU 106 | 4.1 | 2.0 | 6.1 | 1.7 | 7.7 | 1.9 | 2.0 | 1.9 | 1.9 | 1.6 | *Proteobacteria* | *Betaproteobacteria* | *Burkholderiales* | *Burkholderiaceae* | *Burkholderia* | *-* |
| OTU 107 | 1.8 | 2.0 | 1.8 | 8.0 | 1.9 | 1.9 | 2.0 | 1.9 | 1.9 | 1.6 | *Proteobacteria* | *Alphaproteobacteria* | *Sphingomonadales* | *Sphingomonadaceae* | *Sphingobium* | *-* |
| OTU 108 | 1.8 | 2.0 | 1.8 | 7.4 | 1.9 | 4.7 | 2.0 | 1.9 | 1.9 | 1.6 | *Firmicutes* | *Clostridia* | *Clostridiales* | *-* | *-* | *-* |
| OTU 109 | 1.8 | 2.0 | 1.8 | 1.7 | 1.9 | 1.9 | 7.6 | 6.6 | 1.9 | 1.6 | *Firmicutes* | *Clostridia* | *Clostridiales* | *Peptostreptococcaceae* | *Peptostreptococcus* | *-* |
| OTU 110 | 1.8 | 2.0 | 1.8 | 1.7 | 1.9 | 1.9 | 2.0 | 1.9 | 1.9 | 7.0 | *Acidobacteria* | *Acidobacteriia* | *Acidobacteriales* | *Acidobacteriaceae* | *-* | *-* |
| OTU 111 | 1.8 | 4.8 | 1.8 | 1.7 | 1.9 | 1.9 | 3.5 | 1.9 | 3.4 | 7.4 | *Firmicutes* | *Clostridia* | *Clostridiales* | *Ruminococcaceae* | *-* | *-* |
| OTU 112 | 4.1 | 2.0 | 1.8 | 7.2 | 1.9 | 1.9 | 2.0 | 1.9 | 1.9 | 1.6 | *Actinobacteria* | *Actinobacteria* | *Actinomycetales* | *Nocardiaceae* | *Rhodococcus* | *fascians* |
| OTU 113 | 1.8 | 2.0 | 5.2 | 6.4 | 6.0 | 1.9 | 2.0 | 1.9 | 1.9 | 1.6 | *Proteobacteria* | *Alphaproteobacteria* | *Rhizobiales* | *Rhizobiaceae* | *Agrobacterium* | *-* |
| OTU 114 | 1.8 | 2.0 | 1.8 | 7.5 | 6.1 | 1.9 | 2.0 | 1.9 | 3.4 | 1.6 | *Proteobacteria* | *Betaproteobacteria* | *Burkholderiales* | *Oxalobacteraceae* | *-* | *-* |
| OTU 115 | 4.6 | 2.0 | 5.6 | 1.7 | 1.9 | 1.9 | 2.0 | 1.9 | 1.9 | 7.5 | *Cyanobacteria* | *4C0d-2* | *YS2* | *-* | *-* | *-* |
| OTU 116 | 1.8 | 2.0 | 1.8 | 1.7 | 1.9 | 1.9 | 2.0 | 1.9 | 1.9 | 7.4 | *Firmicutes* | *Clostridia* | *Clostridiales* | *Ruminococcaceae* | *-* | *-* |
| OTU 117 | 1.8 | 2.0 | 1.8 | 1.7 | 1.9 | 1.9 | 2.0 | 1.9 | 1.9 | 7.2 | *Proteobacteria* | *Deltaproteobacteria* | *Desulfobacterales* | *Desulfobacteraceae* | *-* | *-* |
| OTU 118 | 1.8 | 2.0 | 1.8 | 1.7 | 1.9 | 1.9 | 2.0 | 1.9 | 1.9 | 7.3 | *Bacteroidetes* | *Bacteroidia* | *Bacteroidales* | *Porphyromonadaceae* | *Parabacteroides* | *-* |
| OTU 119 | 4.1 | 2.0 | 1.8 | 6.9 | 1.9 | 1.9 | 2.0 | 1.9 | 1.9 | 5.5 | *Proteobacteria* | *Betaproteobacteria* | *Burkholderiales* | *Oxalobacteraceae* | *Ralstonia* | *-* |
| OTU 120 | 1.8 | 2.0 | 7.7 | 1.7 | 1.9 | 1.9 | 2.0 | 1.9 | 1.9 | 1.6 | *Bacteroidetes* | *Bacteroidia* | *Bacteroidales* | *[Paraprevotellaceae]* | *Paraprevotella* | *-* |
| OTU 121 | 1.8 | 2.0 | 1.8 | 7.5 | 1.9 | 1.9 | 2.0 | 1.9 | 1.9 | 1.6 | *Firmicutes* | *Erysipelotrichi* | *Erysipelotrichales* | *Erysipelotrichaceae* | *Coprobacillus* | *-* |
| OTU 122 | 1.8 | 2.0 | 1.8 | 7.9 | 1.9 | 1.9 | 2.0 | 1.9 | 1.9 | 1.6 | *Actinobacteria* | *Actinobacteria* | *Actinomycetales* | *Corynebacteriaceae* | *Corynebacterium* | *-* |
| OTU 123 | 1.8 | 2.0 | 1.8 | 5.4 | 6.4 | 3.5 | 6.8 | 3.4 | 5.3 | 8.3 | Unassigned | *-* | *-* | *-* | *-* | *-* |
| OTU 124 | 7.4 | 2.0 | 1.8 | 1.7 | 1.9 | 1.9 | 2.0 | 1.9 | 1.9 | 1.6 | *Actinobacteria* | *Actinobacteria* | *Actinomycetales* | *Micrococcaceae* | *Rothia* | *mucilaginosa* |
| OTU 125 | 1.8 | 2.0 | 7.9 | 1.7 | 1.9 | 1.9 | 2.0 | 1.9 | 1.9 | 1.6 | *Proteobacteria* | *Deltaproteobacteria* | *Desulfarculales* | *Desulfarculaceae* | *-* | *-* |
| OTU 126 | 1.8 | 2.0 | 1.8 | 7.7 | 3.4 | 3.5 | 2.0 | 1.9 | 1.9 | 1.6 | *Firmicutes* | *Clostridia* | *Clostridiales* | *Lachnospiraceae* | *-* | *-* |
| OTU 127 | 1.8 | 2.0 | 1.8 | 1.7 | 1.9 | 4.2 | 2.0 | 1.9 | 1.9 | 7.0 | *Firmicutes* | *Clostridia* | *Clostridiales* | *Lachnospiraceae* | *-* | *-* |
| OTU 128 | 1.8 | 2.0 | 1.8 | 1.7 | 1.9 | 1.9 | 2.0 | 1.9 | 1.9 | 7.8 | *Planctomycetes* | *vadinHA49* | *PeHg47* | *-* | *-* | *-* |
| OTU 129 | 1.8 | 2.0 | 6.4 | 1.7 | 1.9 | 1.9 | 2.0 | 1.9 | 1.9 | 1.6 | *Actinobacteria* | *Actinobacteria* | *Actinomycetales* | *Micrococcaceae* | *-* | *-* |
| OTU 130 | 1.8 | 2.0 | 3.3 | 7.1 | 1.9 | 1.9 | 3.5 | 1.9 | 1.9 | 1.6 | *Bacteroidetes* | *Bacteroidia* | *Bacteroidales* | *Bacteroidaceae* | *Bacteroides* | *-* |
| OTU 131 | 1.8 | 2.0 | 6.5 | 1.7 | 6.7 | 4.2 | 3.5 | 1.9 | 1.9 | 1.6 | *Firmicutes* | *Clostridia* | *Clostridiales* | *-* | *-* | *-* |
| OTU 132 | 4.9 | 3.5 | 7.3 | 1.7 | 1.9 | 5.1 | 3.5 | 1.9 | 1.9 | 6.5 | *Proteobacteria* | *Betaproteobacteria* | *Burkholderiales* | *Comamonadaceae* | *-* | *-* |
| OTU 133 | 1.8 | 2.0 | 7.0 | 1.7 | 1.9 | 1.9 | 2.0 | 1.9 | 1.9 | 1.6 | *Bacteroidetes* | *Cytophagia* | *Cytophagales* | *Cytophagaceae* | *Hymenobacter* | *-* |
| OTU 134 | 1.8 | 2.0 | 1.8 | 7.0 | 1.9 | 1.9 | 2.0 | 1.9 | 1.9 | 1.6 | *Firmicutes* | *Clostridia* | *Clostridiales* | *Eubacteriaceae* | *Anaerofustis* | *-* |
| OTU 135 | 1.8 | 2.0 | 1.8 | 1.7 | 1.9 | 1.9 | 2.0 | 1.9 | 6.8 | 1.6 | *Bacteroidetes* | *Bacteroidia* | *Bacteroidales* | *Bacteroidaceae* | *Bacteroides* | *uniformis* |
| OTU 136 | 1.8 | 2.0 | 1.8 | 6.8 | 1.9 | 4.7 | 2.0 | 1.9 | 1.9 | 1.6 | *Bacteroidetes* | *Bacteroidia* | *Bacteroidales* | *Bacteroidaceae* | *Bacteroides* | *-* |
| OTU 137 | 6.3 | 2.0 | 6.8 | 1.7 | 5.7 | 1.9 | 2.0 | 1.9 | 1.9 | 6.5 | *Actinobacteria* | *Actinobacteria* | *Actinomycetales* | *Corynebacteriaceae* | *Corynebacterium* | *-* |
| OTU 138 | 1.8 | 6.8 | 1.8 | 1.7 | 1.9 | 1.9 | 2.0 | 1.9 | 1.9 | 1.6 | *Firmicutes* | *Clostridia* | *Clostridiales* | *Ruminococcaceae* | *Oscillospira* | *-* |
| OTU 139 | 1.8 | 2.0 | 1.8 | 6.7 | 1.9 | 1.9 | 2.0 | 1.9 | 1.9 | 1.6 | *Actinobacteria* | *Actinobacteria* | *Actinomycetales* | *Micrococcaceae* | *Rothia* | *dentocariosa* |
| OTU 140 | 6.2 | 2.0 | 1.8 | 1.7 | 1.9 | 1.9 | 2.0 | 1.9 | 4.2 | 6.3 | *Proteobacteria* | *Gammaproteobacteria* | *Pseudomonadales* | *Moraxellaceae* | *Acinetobacter* | *-* |
| OTU 141 | 1.8 | 2.0 | 1.8 | 6.9 | 4.7 | 4.2 | 2.0 | 1.9 | 1.9 | 1.6 | *Actinobacteria* | *Thermoleophilia* | *Solirubrobacterales* | *-* | *-* | *-* |
| OTU 142 | 1.8 | 2.0 | 1.8 | 1.7 | 1.9 | 1.9 | 7.3 | 1.9 | 1.9 | 1.6 | *Firmicutes* | *Clostridia* | *Clostridiales* | *-* | *-* | *-* |
| OTU 143 | 1.8 | 2.0 | 1.8 | 6.3 | 1.9 | 1.9 | 2.0 | 1.9 | 1.9 | 1.6 | *Bacteroidetes* | *Bacteroidia* | *Bacteroidales* | *Bacteroidaceae* | *Bacteroides* | *ovatus* |
| OTU 144 | 5.2 | 2.0 | 1.8 | 1.7 | 7.0 | 1.9 | 2.0 | 1.9 | 1.9 | 1.6 | *Actinobacteria* | *Actinobacteria* | *Actinomycetales* | *Corynebacteriaceae* | *Corynebacterium* | *-* |
| OTU 145 | 1.8 | 2.0 | 1.8 | 1.7 | 1.9 | 1.9 | 2.0 | 1.9 | 1.9 | 6.5 | *Firmicutes* | *Clostridia* | *Clostridiales* | *Clostridiaceae* | *Clostridium* | *-* |
| OTU 146 | 5.8 | 2.0 | 6.8 | 1.7 | 1.9 | 1.9 | 2.0 | 1.9 | 1.9 | 1.6 | *Bacteroidetes* | *Bacteroidia* | *Bacteroidales* | *Prevotellaceae* | *Prevotella* | *-* |
| OTU 147 | 1.8 | 2.0 | 1.8 | 1.7 | 1.9 | 1.9 | 2.0 | 1.9 | 1.9 | 6.9 | Unassigned | *-* | *-* | *-* | *-* | *-* |
| OTU 148 | 1.8 | 2.0 | 1.8 | 1.7 | 1.9 | 1.9 | 2.0 | 7.4 | 6.9 | 1.6 | Unassigned | *-* | *-* | *-* | *-* | *-* |
| OTU 149 | 1.8 | 2.0 | 5.8 | 1.7 | 1.9 | 1.9 | 2.0 | 1.9 | 1.9 | 5.6 | *Proteobacteria* | *Deltaproteobacteria* | *Desulfovibrionales* | *Desulfovibrionaceae* | *Desulfovibrio* | *-* |
| OTU 150 | 1.8 | 2.0 | 7.4 | 1.7 | 1.9 | 1.9 | 2.0 | 1.9 | 1.9 | 1.6 | *Bacteroidetes* | *Cytophagia* | *Cytophagales* | *Cytophagaceae* | *Hymenobacter* | *-* |
| OTU 151 | 1.8 | 2.0 | 7.0 | 1.7 | 4.7 | 1.9 | 2.0 | 1.9 | 1.9 | 1.6 | *Actinobacteria* | *Actinobacteria* | *Actinomycetales* | *Kineosporiaceae* | *-* | *-* |
| OTU 152 | 1.8 | 2.0 | 1.8 | 1.7 | 1.9 | 1.9 | 2.0 | 1.9 | 1.9 | 7.2 | *Proteobacteria* | *Deltaproteobacteria* | *Desulfovibrionales* | *Desulfovibrionaceae* | *-* | *-* |
| OTU 153 | 4.6 | 3.5 | 1.8 | 1.7 | 1.9 | 1.9 | 2.0 | 1.9 | 1.9 | 6.5 | *Proteobacteria* | *Deltaproteobacteria* | *Desulfovibrionales* | *Desulfovibrionaceae* | *Bilophila* | *-* |
| OTU 154 | 1.8 | 2.0 | 6.4 | 1.7 | 1.9 | 1.9 | 2.0 | 1.9 | 1.9 | 1.6 | *Proteobacteria* | *Deltaproteobacteria* | *Desulfovibrionales* | *Desulfovibrionaceae* | *-* | *-* |
| OTU 155 | 7.4 | 8.7 | 6.4 | 6.5 | 1.9 | 8.6 | 6.5 | 10.0 | 1.9 | 1.6 | *Firmicutes* | *Bacilli* | *Lactobacillales* | *Carnobacteriaceae* | *Trichococcus* | *-* |
| OTU 156 | 5.2 | 3.5 | 1.8 | 1.7 | 6.2 | 1.9 | 2.0 | 1.9 | 1.9 | 1.6 | *Armatimonadetes* | *[Fimbriimonadia]* | *[Fimbriimonadales]* | *[Fimbriimonadaceae]* | *Fimbriimonas* | *-* |
| OTU 157 | 4.9 | 2.0 | 1.8 | 1.7 | 1.9 | 5.7 | 4.8 | 1.9 | 4.2 | 3.1 | *Firmicutes* | *Clostridia* | *Clostridiales* | *Clostridiaceae* | *Clostridium* | *-* |
| OTU 158 | 1.8 | 2.0 | 1.8 | 1.7 | 6.2 | 1.9 | 2.0 | 1.9 | 1.9 | 1.6 | *Proteobacteria* | *Alphaproteobacteria* | *Rhizobiales* | *Methylocystaceae* | *-* | *-* |
| OTU 159 | 1.8 | 3.5 | 4.6 | 1.7 | 1.9 | 5.3 | 3.5 | 7.7 | 6.8 | 1.6 | Unassigned | *-* | *-* | *-* | *-* | *-* |
| OTU 160 | 1.8 | 3.5 | 1.8 | 1.7 | 1.9 | 1.9 | 2.0 | 1.9 | 1.9 | 6.2 | *Firmicutes* | *Clostridia* | *Clostridiales* | *-* | *-* | *-* |
| OTU 161 | 1.8 | 2.0 | 3.3 | 1.7 | 6.8 | 4.2 | 2.0 | 1.9 | 3.4 | 1.6 | *Firmicutes* | *Clostridia* | *Clostridiales* | *Clostridiaceae* | *Clostridium* | *perfringens* |
| OTU 162 | 1.8 | 3.5 | 1.8 | 6.2 | 1.9 | 1.9 | 2.0 | 1.9 | 1.9 | 1.6 | *Firmicutes* | *Clostridia* | *Clostridiales* | *Ruminococcaceae* | *-* | *-* |
| OTU 163 | 6.4 | 2.0 | 1.8 | 1.7 | 5.0 | 1.9 | 2.0 | 1.9 | 1.9 | 1.6 | *Proteobacteria* | *Gammaproteobacteria* | *Oceanospirillales* | *Halomonadaceae* | *-* | *-* |
| OTU 164 | 1.8 | 2.0 | 1.8 | 1.7 | 1.9 | 3.5 | 2.0 | 1.9 | 1.9 | 6.4 | *Proteobacteria* | *Gammaproteobacteria* | *Pseudomonadales* | *Moraxellaceae* | *Acinetobacter* | *-* |
| OTU 165 | 6.0 | 5.1 | 7.1 | 6.2 | 1.9 | 8.0 | 5.8 | 7.1 | 5.7 | 1.6 | Unassigned | *-* | *-* | *-* | *-* | *-* |
| OTU 166 | 1.8 | 2.0 | 1.8 | 1.7 | 1.9 | 1.9 | 2.0 | 7.0 | 1.9 | 1.6 | *Firmicutes* | *Clostridia* | *Clostridiales* | *Lachnospiraceae* | *-* | *-* |
| OTU 167 | 1.8 | 2.0 | 1.8 | 1.7 | 1.9 | 1.9 | 4.3 | 1.9 | 1.9 | 6.7 | *Firmicutes* | *Clostridia* | *Clostridiales* | *Lachnospiraceae* | *Dorea* | *-* |
| OTU 168 | 1.8 | 2.0 | 1.8 | 6.3 | 1.9 | 1.9 | 2.0 | 1.9 | 1.9 | 1.6 | *Firmicutes* | *Clostridia* | *Clostridiales* | *Ruminococcaceae* | *Oscillospira* | *-* |
| OTU 169 | 1.8 | 2.0 | 1.8 | 1.7 | 1.9 | 1.9 | 2.0 | 6.6 | 1.9 | 1.6 | *Actinobacteria* | *Actinobacteria* | *Actinomycetales* | *Intrasporangiaceae* | *Terracoccus* | *-* |
| OTU 170 | 1.8 | 4.3 | 3.3 | 1.7 | 6.8 | 1.9 | 2.0 | 1.9 | 1.9 | 1.6 | *Firmicutes* | *Bacilli* | *Lactobacillales* | *Enterococcaceae* | *-* | *-* |
| OTU 171 | 4.1 | 2.0 | 1.8 | 5.7 | 1.9 | 1.9 | 2.0 | 1.9 | 1.9 | 1.6 | *Firmicutes* | *Clostridia* | *Clostridiales* | *Lachnospiraceae* | *Blautia* | *-* |
| OTU 172 | 4.9 | 2.0 | 4.6 | 5.6 | 6.4 | 1.9 | 4.3 | 1.9 | 1.9 | 1.6 | *Proteobacteria* | *Betaproteobacteria* | *Burkholderiales* | *Oxalobacteraceae* | *Ralstonia* | *-* |
| OTU 173 | 1.8 | 2.0 | 1.8 | 1.7 | 1.9 | 1.9 | 2.0 | 1.9 | 1.9 | 6.0 | *Deferribacteres* | *Deferribacteres* | *Deferribacterales* | *Deferribacteraceae* | *Mucispirillum* | *-* |
| OTU 174 | 1.8 | 2.0 | 1.8 | 1.7 | 6.2 | 4.2 | 2.0 | 1.9 | 1.9 | 6.9 | Unassigned | *-* | *-* | *-* | *-* | *-* |
| OTU 175 | 5.2 | 2.0 | 1.8 | 6.7 | 1.9 | 1.9 | 2.0 | 1.9 | 1.9 | 1.6 | *Proteobacteria* | *Betaproteobacteria* | *Burkholderiales* | *Oxalobacteraceae* | *Oxalobacter* | *formigenes* |
| OTU 176 | 1.8 | 2.0 | 1.8 | 1.7 | 1.9 | 1.9 | 2.0 | 1.9 | 1.9 | 5.8 | *Firmicutes* | *Clostridia* | *Clostridiales* | *Lachnospiraceae* | *-* | *-* |
| OTU 177 | 1.8 | 2.0 | 1.8 | 1.7 | 1.9 | 1.9 | 2.0 | 1.9 | 1.9 | 7.0 | *Proteobacteria* | *Deltaproteobacteria* | *Desulfovibrionales* | *Desulfovibrionaceae* | *Bilophila* | *-* |
| OTU 178 | 1.8 | 3.5 | 1.8 | 1.7 | 1.9 | 1.9 | 2.0 | 1.9 | 1.9 | 6.4 | Unassigned | *-* | *-* | *-* | *-* | *-* |
| OTU 179 | 5.4 | 2.0 | 3.3 | 1.7 | 1.9 | 1.9 | 2.0 | 1.9 | 1.9 | 1.6 | *Actinobacteria* | *Actinobacteria* | *Actinomycetales* | *Corynebacteriaceae* | *Corynebacterium* | *-* |
| OTU 180 | 5.6 | 2.0 | 1.8 | 1.7 | 1.9 | 1.9 | 2.0 | 1.9 | 1.9 | 1.6 | *Proteobacteria* | *Alphaproteobacteria* | *Rhodospirillales* | *Rhodospirillaceae* | *-* | *-* |
| OTU 181 | 1.8 | 2.0 | 1.8 | 1.7 | 1.9 | 1.9 | 2.0 | 1.9 | 1.9 | 7.6 | *Bacteroidetes* | *Bacteroidia* | *Bacteroidales* | *Porphyromonadaceae* | *Parabacteroides* | *-* |
| OTU 182 | 1.8 | 2.0 | 5.6 | 1.7 | 1.9 | 1.9 | 2.0 | 1.9 | 1.9 | 1.6 | *Cyanobacteria* | *4C0d-2* | *MLE1-12* | *-* | *-* | *-* |
| OTU 183 | 3.3 | 5.4 | 1.8 | 3.2 | 1.9 | 1.9 | 2.0 | 3.4 | 4.7 | 7.9 | *Proteobacteria* | *Gammaproteobacteria* | *Pseudomonadales* | *Pseudomonadaceae* | *Pseudomonas* | *-* |
| OTU 184 | 5.4 | 2.0 | 1.8 | 1.7 | 6.0 | 1.9 | 2.0 | 1.9 | 1.9 | 5.1 | *Proteobacteria* | *Betaproteobacteria* | *Burkholderiales* | *Comamonadaceae* | *-* | *-* |
| OTU 185 | 1.8 | 2.0 | 5.4 | 1.7 | 1.9 | 1.9 | 2.0 | 1.9 | 1.9 | 1.6 | *Proteobacteria* | *Alphaproteobacteria* | *Rhodospirillales* | *Acetobacteraceae* | *-* | *-* |
| OTU 186 | 1.8 | 2.0 | 1.8 | 1.7 | 1.9 | 1.9 | 2.0 | 1.9 | 5.5 | 1.6 | *Firmicutes* | *Clostridia* | *Clostridiales* | *[Tissierellaceae]* | *Peptoniphilus* | *-* |
| OTU 187 | 4.9 | 2.0 | 1.8 | 1.7 | 5.3 | 1.9 | 2.0 | 1.9 | 1.9 | 5.6 | *Proteobacteria* | *Gammaproteobacteria* | *Pseudomonadales* | *Moraxellaceae* | *Enhydrobacter* | *-* |
| OTU 188 | 5.4 | 2.0 | 1.8 | 1.7 | 1.9 | 1.9 | 2.0 | 1.9 | 1.9 | 1.6 | *Proteobacteria* | *Betaproteobacteria* | *Neisseriales* | *Neisseriaceae* | *Neisseria* | *subflava* |
| OTU 189 | 1.8 | 2.0 | 1.8 | 1.7 | 5.3 | 1.9 | 2.0 | 1.9 | 1.9 | 1.6 | *Proteobacteria* | *Alphaproteobacteria* | *Rhodospirillales* | *Acetobacteraceae* | *-* | *-* |
| OTU 190 | 1.8 | 2.0 | 1.8 | 1.7 | 5.5 | 1.9 | 2.0 | 1.9 | 1.9 | 1.6 | *Actinobacteria* | *Actinobacteria* | *Actinomycetales* | *Nocardioidaceae* | *-* | *-* |
| OTU 191 | 1.8 | 2.0 | 1.8 | 1.7 | 1.9 | 1.9 | 2.0 | 1.9 | 1.9 | 7.4 | *Planctomycetes* | *vadinHA49* | *PeHg47* | *-* | *-* | *-* |
| OTU 192 | 1.8 | 2.0 | 1.8 | 1.7 | 1.9 | 1.9 | 2.0 | 1.9 | 1.9 | 6.0 | *Bacteroidetes* | *Bacteroidia* | *Bacteroidales* | *Bacteroidaceae* | *Bacteroides* | *ovatus* |
| OTU 193 | 1.8 | 2.0 | 5.9 | 6.3 | 1.9 | 1.9 | 2.0 | 1.9 | 1.9 | 1.6 | *Firmicutes* | *Clostridia* | *Clostridiales* | *Lachnospiraceae* | *-* | *-* |
| OTU 194 | 1.8 | 2.0 | 1.8 | 1.7 | 1.9 | 1.9 | 2.0 | 1.9 | 1.9 | 7.0 | *Bacteroidetes* | *Bacteroidia* | *Bacteroidales* | *Rikenellaceae* | *-* | *-* |
| OTU 195 | 6.7 | 2.0 | 1.8 | 1.7 | 1.9 | 1.9 | 2.0 | 1.9 | 1.9 | 1.6 | *Firmicutes* | *Clostridia* | *Clostridiales* | *Ruminococcaceae* | *-* | *-* |
| OTU 196 | 1.8 | 3.5 | 3.3 | 4.6 | 5.0 | 5.3 | 4.8 | 3.4 | 3.4 | 3.1 | *Proteobacteria* | *Gammaproteobacteria* | *Enterobacteriales* | *Enterobacteriaceae* | *Plesiomonas* | *shigelloides* |
| OTU 197 | 1.8 | 2.0 | 1.8 | 1.7 | 5.0 | 1.9 | 2.0 | 1.9 | 1.9 | 1.6 | *Acidobacteria* | *Acidobacteriia* | *Acidobacteriales* | *Acidobacteriaceae* | *Terriglobus* | *-* |
| OTU 199 | 1.8 | 2.0 | 5.9 | 1.7 | 1.9 | 1.9 | 2.0 | 1.9 | 1.9 | 1.6 | *Actinobacteria* | *Actinobacteria* | *Actinomycetales* | *Corynebacteriaceae* | *Corynebacterium* | *-* |
| OTU 200 | 4.1 | 6.8 | 1.8 | 1.7 | 1.9 | 5.7 | 2.0 | 8.1 | 1.9 | 1.6 | Unassigned | *-* | *-* | *-* | *-* | *-* |
| OTU 201 | 1.8 | 2.0 | 1.8 | 1.7 | 1.9 | 1.9 | 2.0 | 8.3 | 1.9 | 1.6 | *Firmicutes* | *Bacilli* | *Lactobacillales* | *Aerococcaceae* | *Aerococcus* | *-* |
| OTU 202 | 1.8 | 2.0 | 1.8 | 1.7 | 1.9 | 1.9 | 2.0 | 1.9 | 1.9 | 6.1 | *Firmicutes* | *Clostridia* | *Clostridiales* | *Lachnospiraceae* | *Coprococcus* | *-* |
| OTU 204 | 4.9 | 2.0 | 1.8 | 3.2 | 5.0 | 4.2 | 2.0 | 4.2 | 1.9 | 3.1 | *Fusobacteria* | *Fusobacteriia* | *Fusobacteriales* | *Leptotrichiaceae* | *-* | *-* |
| OTU 205 | 1.8 | 4.3 | 1.8 | 1.7 | 1.9 | 1.9 | 2.0 | 1.9 | 1.9 | 4.4 | *Firmicutes* | *Clostridia* | *Clostridiales* | *Lachnospiraceae* | *-* | *-* |
| OTU 206 | 1.8 | 2.0 | 1.8 | 1.7 | 1.9 | 1.9 | 2.0 | 1.9 | 1.9 | 6.5 | *Proteobacteria* | *Gammaproteobacteria* | *Pasteurellales* | *Pasteurellaceae* | *Aggregatibacter* | *-* |
| OTU 207 | 1.8 | 2.0 | 1.8 | 1.7 | 1.9 | 1.9 | 2.0 | 1.9 | 1.9 | 5.3 | Unassigned | *-* | *-* | *-* | *-* | *-* |
| OTU 209 | 1.8 | 3.5 | 4.1 | 3.2 | 5.0 | 4.2 | 3.5 | 3.4 | 3.4 | 1.6 | *Firmicutes* | *Clostridia* | *Clostridiales* | *Peptostreptococcaceae* | *-* | *-* |
| OTU 210 | 1.8 | 2.0 | 1.8 | 1.7 | 1.9 | 1.9 | 2.0 | 1.9 | 1.9 | 5.6 | *Bacteroidetes* | *Bacteroidia* | *Bacteroidales* | *Rikenellaceae* | *-* | *-* |
| OTU 212 | 1.8 | 2.0 | 1.8 | 5.9 | 1.9 | 1.9 | 2.0 | 1.9 | 1.9 | 1.6 | *Firmicutes* | *Clostridia* | *Clostridiales* | *Dehalobacteriaceae* | *-* | *-* |
| OTU 213 | 1.8 | 6.9 | 1.8 | 1.7 | 3.4 | 1.9 | 2.0 | 1.9 | 1.9 | 6.7 | *Firmicutes* | *Clostridia* | *Clostridiales* | *-* | *-* | *-* |
| OTU 214 | 1.8 | 2.0 | 1.8 | 1.7 | 1.9 | 1.9 | 2.0 | 1.9 | 1.9 | 5.3 | *Proteobacteria* | *Gammaproteobacteria* | *Pseudomonadales* | *Moraxellaceae* | *Acinetobacter* | *-* |
| OTU 215 | 5.6 | 2.0 | 1.8 | 5.2 | 1.9 | 1.9 | 2.0 | 1.9 | 1.9 | 1.6 | *Firmicutes* | *Clostridia* | *Clostridiales* | *Veillonellaceae* | *Veillonella* | *dispar* |
| OTU 216 | 1.8 | 2.0 | 1.8 | 1.7 | 1.9 | 1.9 | 2.0 | 6.9 | 1.9 | 1.6 | Unassigned | *-* | *-* | *-* | *-* | *-* |
| OTU 217 | 5.6 | 2.0 | 1.8 | 1.7 | 1.9 | 1.9 | 2.0 | 1.9 | 1.9 | 1.6 | *Proteobacteria* | *Alphaproteobacteria* | *Rhizobiales* | *Bradyrhizobiaceae* | *-* | *-* |
| OTU 218 | 1.8 | 2.0 | 1.8 | 1.7 | 5.5 | 1.9 | 4.3 | 1.9 | 4.2 | 1.6 | *Firmicutes* | *Bacilli* | *Bacillales* | *-* | *-* | *-* |
| OTU 219 | 1.8 | 2.0 | 1.8 | 1.7 | 1.9 | 5.3 | 2.0 | 1.9 | 1.9 | 1.6 | *Proteobacteria* | *Alphaproteobacteria* | *Rhodobacterales* | *Rhodobacteraceae* | *Amaricoccus* | *-* |
| OTU 220 | 1.8 | 5.9 | 1.8 | 3.2 | 1.9 | 1.9 | 2.0 | 1.9 | 1.9 | 7.6 | *Firmicutes* | *Clostridia* | *Clostridiales* | *Lachnospiraceae* | *-* | *-* |
| OTU 221 | 1.8 | 2.0 | 1.8 | 1.7 | 1.9 | 1.9 | 2.0 | 1.9 | 1.9 | 6.1 | Unassigned | *-* | *-* | *-* | *-* | *-* |
| OTU 222 | 3.3 | 6.2 | 3.3 | 1.7 | 1.9 | 1.9 | 2.0 | 1.9 | 1.9 | 1.6 | Unassigned | *-* | *-* | *-* | *-* | *-* |
| OTU 223 | 6.3 | 2.0 | 1.8 | 1.7 | 5.3 | 1.9 | 2.0 | 4.7 | 1.9 | 1.6 | *Firmicutes* | *Bacilli* | *Lactobacillales* | *Streptococcaceae* | *Streptococcus* | *-* |
| OTU 224 | 1.8 | 3.5 | 1.8 | 1.7 | 1.9 | 5.7 | 2.0 | 1.9 | 1.9 | 1.6 | *Proteobacteria* | *Alphaproteobacteria* | *Sphingomonadales* | *Sphingomonadaceae* | *-* | *-* |
| OTU 225 | 1.8 | 2.0 | 1.8 | 1.7 | 5.0 | 1.9 | 2.0 | 1.9 | 1.9 | 1.6 | *Proteobacteria* | *Betaproteobacteria* | *Gallionellales* | *Gallionellaceae* | *Gallionella* | *-* |
| OTU 226 | 1.8 | 2.0 | 1.8 | 1.7 | 1.9 | 1.9 | 2.0 | 6.1 | 1.9 | 1.6 | Unassigned | *-* | *-* | *-* | *-* | *-* |
| OTU 227 | 1.8 | 2.0 | 1.8 | 1.7 | 1.9 | 1.9 | 2.0 | 10.1 | 1.9 | 3.1 | Unassigned | *-* | *-* | *-* | *-* | *-* |
| OTU 228 | 5.2 | 2.0 | 1.8 | 1.7 | 1.9 | 1.9 | 2.0 | 1.9 | 1.9 | 1.6 | *Firmicutes* | *Clostridia* | *Clostridiales* | *Ruminococcaceae* | *Ruminococcus* | *-* |
| OTU 229 | 1.8 | 5.4 | 1.8 | 4.9 | 1.9 | 5.3 | 2.0 | 1.9 | 1.9 | 1.6 | *Firmicutes* | *Clostridia* | *Clostridiales* | *Clostridiaceae* | *-* | *-* |
| OTU 230 | 1.8 | 2.0 | 1.8 | 1.7 | 3.4 | 1.9 | 2.0 | 1.9 | 1.9 | 4.4 | Unassigned | *-* | *-* | *-* | *-* | *-* |
| OTU 231 | 1.8 | 2.0 | 5.9 | 1.7 | 5.3 | 1.9 | 2.0 | 1.9 | 1.9 | 1.6 | *Proteobacteria* | *Alphaproteobacteria* | *Rhizobiales* | *Methylocystaceae* | *-* | *-* |
| OTU 232 | 1.8 | 2.0 | 1.8 | 1.7 | 1.9 | 1.9 | 2.0 | 1.9 | 4.2 | 1.6 | *Proteobacteria* | *Alphaproteobacteria* | *Rhodospirillales* | *Acetobacteraceae* | *-* | *-* |
| OTU 233 | 1.8 | 2.0 | 1.8 | 1.7 | 4.2 | 1.9 | 2.0 | 1.9 | 1.9 | 3.9 | Unassigned | *-* | *-* | *-* | *-* | *-* |
| OTU 234 | 1.8 | 2.0 | 1.8 | 1.7 | 5.0 | 1.9 | 2.0 | 1.9 | 1.9 | 1.6 | *Bacteroidetes* | *Bacteroidia* | *Bacteroidales* | *Bacteroidaceae* | *Bacteroides* | *-* |
| OTU 235 | 1.8 | 2.0 | 1.8 | 1.7 | 5.5 | 1.9 | 2.0 | 1.9 | 1.9 | 1.6 | *Bacteroidetes* | *Bacteroidia* | *Bacteroidales* | *Bacteroidaceae* | *Bacteroides* | *-* |
| OTU 236 | 1.8 | 2.0 | 1.8 | 1.7 | 1.9 | 1.9 | 2.0 | 1.9 | 1.9 | 3.9 | *Bacteroidetes* | *Bacteroidia* | *Bacteroidales* | *Rikenellaceae* | *-* | *-* |
| OTU 237 | 1.8 | 2.0 | 1.8 | 1.7 | 1.9 | 5.1 | 2.0 | 1.9 | 1.9 | 1.6 | *Bacteroidetes* | *Bacteroidia* | *Bacteroidales* | *Bacteroidaceae* | *Bacteroides* | *-* |
| OTU 238 | 1.8 | 2.0 | 1.8 | 1.7 | 1.9 | 1.9 | 2.0 | 8.8 | 1.9 | 1.6 | Unassigned | *-* | *-* | *-* | *-* | *-* |
| OTU 239 | 1.8 | 4.8 | 4.1 | 1.7 | 1.9 | 1.9 | 2.0 | 1.9 | 4.2 | 1.6 | *Fusobacteria* | *Fusobacteriia* | *Fusobacteriales* | *Fusobacteriaceae* | *Fusobacterium* | *-* |
| OTU 240 | 1.8 | 2.0 | 1.8 | 1.7 | 1.9 | 1.9 | 2.0 | 1.9 | 5.0 | 10.3 | *Bacteroidetes* | *Bacteroidia* | *Bacteroidales* | *Porphyromonadaceae* | *Parabacteroides* | *-* |
| OTU 241 | 5.4 | 2.0 | 1.8 | 1.7 | 1.9 | 1.9 | 2.0 | 1.9 | 1.9 | 1.6 | *Firmicutes* | *Clostridia* | *Clostridiales* | *[Tissierellaceae]* | *Anaerococcus* | *-* |
| OTU 242 | 3.3 | 2.0 | 5.4 | 5.2 | 1.9 | 1.9 | 2.0 | 1.9 | 1.9 | 1.6 | *Proteobacteria* | *Betaproteobacteria* | *Burkholderiales* | *Comamonadaceae* | *-* | *-* |
| OTU 243 | 3.3 | 4.3 | 4.1 | 1.7 | 5.3 | 3.5 | 2.0 | 3.4 | 3.4 | 1.6 | *Fusobacteria* | *Fusobacteriia* | *Fusobacteriales* | *Fusobacteriaceae* | *Cetobacterium* | *somerae* |
| OTU 244 | 1.8 | 2.0 | 1.8 | 1.7 | 1.9 | 1.9 | 2.0 | 1.9 | 1.9 | 6.5 | *Firmicutes* | *Clostridia* | *Clostridiales* | *Ruminococcaceae* | *-* | *-* |
| OTU 245 | 1.8 | 3.5 | 4.1 | 1.7 | 1.9 | 1.9 | 2.0 | 1.9 | 1.9 | 4.8 | *Synergistetes* | *Synergistia* | *Synergistales* | *Synergistaceae* | *-* | *-* |
| OTU 246 | 1.8 | 2.0 | 5.8 | 1.7 | 1.9 | 1.9 | 2.0 | 1.9 | 1.9 | 1.6 | *Actinobacteria* | *Coriobacteriia* | *Coriobacteriales* | *Coriobacteriaceae* | *-* | *-* |
| OTU 247 | 1.8 | 2.0 | 1.8 | 1.7 | 1.9 | 1.9 | 2.0 | 1.9 | 1.9 | 5.5 | *Bacteroidetes* | *Bacteroidia* | *Bacteroidales* | *Rikenellaceae* | *-* | *-* |
| OTU 248 | 4.6 | 2.0 | 1.8 | 1.7 | 1.9 | 1.9 | 2.0 | 1.9 | 1.9 | 1.6 | *Firmicutes* | *Clostridia* | *Clostridiales* | *Lachnospiraceae* | *-* | *-* |
| OTU 249 | 1.8 | 5.6 | 1.8 | 1.7 | 1.9 | 3.5 | 3.5 | 5.3 | 5.7 | 1.6 | Unassigned | *-* | *-* | *-* | *-* | *-* |
| OTU 250 | 1.8 | 2.0 | 4.1 | 1.7 | 1.9 | 1.9 | 2.0 | 1.9 | 6.0 | 7.6 | *Planctomycetes* | *vadinHA49* | *PeHg47* | *-* | *-* | *-* |
| OTU 251 | 1.8 | 2.0 | 1.8 | 7.5 | 1.9 | 1.9 | 2.0 | 1.9 | 1.9 | 1.6 | *Firmicutes* | *Clostridia* | *Clostridiales* | *-* | *-* | *-* |
| OTU 252 | 1.8 | 2.0 | 1.8 | 1.7 | 1.9 | 1.9 | 2.0 | 1.9 | 1.9 | 6.7 | *Firmicutes* | *Clostridia* | *Clostridiales* | *Veillonellaceae* | *-* | *-* |
| OTU 253 | 1.8 | 2.0 | 1.8 | 1.7 | 1.9 | 1.9 | 2.0 | 4.2 | 1.9 | 1.6 | Unassigned | *-* | *-* | *-* | *-* | *-* |
| OTU 254 | 3.3 | 2.0 | 5.8 | 1.7 | 1.9 | 1.9 | 2.0 | 1.9 | 1.9 | 1.6 | *Actinobacteria* | *Actinobacteria* | *Actinomycetales* | *Actinomycetaceae* | *Actinomyces* | *-* |
| OTU 255 | 1.8 | 2.0 | 1.8 | 1.7 | 1.9 | 1.9 | 2.0 | 1.9 | 1.9 | 5.1 | *Proteobacteria* | *Deltaproteobacteria* | *Desulfovibrionales* | *Desulfovibrionaceae* | *-* | *-* |
| OTU 256 | 1.8 | 2.0 | 1.8 | 1.7 | 5.3 | 1.9 | 3.5 | 1.9 | 1.9 | 1.6 | *Proteobacteria* | *Alphaproteobacteria* | *Rhodospirillales* | *Rhodospirillaceae* | *-* | *-* |
| OTU 257 | 1.8 | 2.0 | 1.8 | 1.7 | 1.9 | 1.9 | 2.0 | 4.7 | 1.9 | 1.6 | Unassigned | *-* | *-* | *-* | *-* | *-* |
| OTU 258 | 3.3 | 2.0 | 3.3 | 1.7 | 1.9 | 1.9 | 5.8 | 1.9 | 1.9 | 1.6 | *Firmicutes* | *Clostridia* | *Clostridiales* | *Lachnospiraceae* | *Roseburia* | *-* |
| OTU 259 | 1.8 | 5.6 | 1.8 | 1.7 | 1.9 | 1.9 | 2.0 | 3.4 | 1.9 | 1.6 | *Firmicutes* | *Clostridia* | *Clostridiales* | *Ruminococcaceae* | *-* | *-* |
| OTU 260 | 1.8 | 2.0 | 4.9 | 1.7 | 1.9 | 1.9 | 2.0 | 1.9 | 4.7 | 1.6 | *Actinobacteria* | *Actinobacteria* | *Actinomycetales* | *Micrococcaceae* | *Kocuria* | *palustris* |
| OTU 261 | 1.8 | 2.0 | 1.8 | 1.7 | 1.9 | 1.9 | 2.0 | 5.7 | 6.6 | 1.6 | Unassigned | *-* | *-* | *-* | *-* | *-* |

OTUs were assigned a taxonomy (with ≥90% identity) from the Greengenes curated database [1]. Koalas K1 – K5 were clinically normal (wet bottom absent), whilst koalas K31 – K70 had wet bottom.

^^^ Classification could not be made to this level

1. DeSantis TZ, Hugenholtz P, Larsen N, Rojas M, Brodie EL, Keller K, et al. Greengenes, a chimera-checked 16S rRNA gene database and workbench compatible with ARB. Appl Environ Microbiol. 2006;72(7):5069-72. doi: 10.1128/aem.03006-05.
