# Supplementary material for: Variation in the microbiome of the urogenital tract of Chlamydia-free female koalas (Phascolarctos cinereus) with and without ‘wet bottom’
Source: PLoS One. 2018 Mar 26;13(3):e0194881. doi: 10.1371/journal.pone.0194881 (PMC5868818; doi:10.1371/journal.pone.0194881)
Supplement: S3 Table — (DOCX) [file pone.0194881.s003.docx]

**S3 Table. Statistical values of differential abundance comparisons between DESeq2 normalised reads [1] in koalas with (K31 – K70) and without (K1 – K5) wet bottom (WB).**

| **OTU ID** | **Base Mean** | **LFC^*^** | **LFC standard error** | **Wald test Z statistic** | **BH Adjusted *P* value^+^** | **Higher abundance group** | **Present in samples (n/5)** | | **Taxonomic classification** | | | | | |
| --- | --- | --- | --- | --- | --- | --- | --- | --- | --- | --- | --- | --- | --- | --- |
|  |  |  |  |  |  |  | **WB absent** | **WB present** | **Phylum** | **Class** | **Order** | **Family** | **Genus** | **Species** |
| OTU 1 | 71520.47 | -1.98 | 0.80 | -2.46 | **0.045** | WB present | 5 | 5 | *Firmicutes* | *Bacilli* | *Lactobacillales* | *Aerococcaceae* | *Aerococcus* | *-^^^*- |
| OTU 2 | 61538.06 | 2.79 | 0.84 | 3.31 | **0.006** | WB absent | 5 | 5 | *Firmicutes* | *Bacilli* | *Lactobacillales* | *Aerococcaceae* | *Aerococcus* | *-* |
| OTU 3 | 15469.07 | -4.63 | 1.64 | -2.82 | - | WB present | 5 | 5 | *Proteobacteria* | *Gammaproteobacteria* | *Enterobacteriales* | *Enterobacteriaceae* | *-* | *-* |
| OTU 4 | 17147.87 | 3.88 | 1.10 | 3.52 | **0.004** | WB absent | 5 | 5 | *Firmicutes* | *Bacilli* | *Lactobacillales* | *Aerococcaceae* | *Aerococcus* | *-* |
| OTU 5 | 8503.18 | -2.60 | 1.57 | -1.66 | - | WB absent | 5 | 4 | *Bacteroidetes* | *Bacteroidia* | *Bacteroidales* | *Porphyromonadaceae* | *Parabacteroides* | *-* |
| OTU 6 | 10981.84 | 0.52 | 1.16 | 0.45 | 0.729 | WB absent | 5 | 5 | Unassigned | *-* | *-* | *-* | *-* | *-* |
| OTU 7 | 7479.20 | 1.70 | 1.23 | 1.39 | 0.236 | WB absent | 5 | 5 | *Firmicutes* | *Clostridia* | *Clostridiales* | *Clostridiaceae* | *Clostridium* | *-* |
| OTU 8 | 3575.92 | -4.32 | 1.53 | -2.81 | - | WB absent | 4 | 5 | *Bacteroidetes* | *Bacteroidia* | *Bacteroidales* | *Porphyromonadaceae* | *Parabacteroides* | *-* |
| OTU 9 | 4133.53 | 2.17 | 1.44 | 1.51 | 0.208 | WB present | 4 | 5 | *Actinobacteria* | *Coriobacteriia* | *Coriobacteriales* | *Coriobacteriaceae* | *-* | *-* |
| OTU 10 | 15826.26 | 0.29 | 1.02 | 0.28 | 0.844 | WB absent | 5 | 5 | *Firmicutes* | *Bacilli* | *Lactobacillales* | *Aerococcaceae* | *Facklamia* | *-* |
| OTU 11 | 3525.98 | -3.29 | 1.63 | -2.02 | - | WB absent | 4 | 4 | *Bacteroidetes* | *Bacteroidia* | *Bacteroidales* | *Porphyromonadaceae* | *Parabacteroides* | *-* |
| OTU 12 | 2503.43 | -2.33 | 1.56 | -1.49 | - | WB absent | 5 | 4 | *Firmicutes* | *Clostridia* | *Clostridiales* | *Lachnospiraceae* | *-* | *-* |
| OTU 13 | 1299.60 | -3.31 | 1.51 | -2.19 | - | WB absent | 5 | 3 | *Firmicutes* | *Clostridia* | *Clostridiales* | *Lachnospiraceae* | *-* | *-* |
| OTU 14 | 1603.98 | 0.18 | 1.44 | 0.13 | 0.911 | WB present | 3 | 5 | *Actinobacteria* | *Actinobacteria* | *Actinomycetales* | *Corynebacteriaceae* | *Corynebacterium* | *-* |
| OTU 15 | 1406.37 | -2.27 | 1.48 | -1.53 | 0.202 | WB present | 4 | 5 | *TM7* | *TM7-3* | *I025* | *Rs-045* | *-* | *-* |
| OTU 16 | 2807.38 | -9.41 | 1.37 | -6.87 | - | WB present | 3 | 5 | *Bacteroidetes* | *Bacteroidia* | *Bacteroidales* | *Porphyromonadaceae* | *Porphyromonas* | *-* |
| OTU 17 | 1008.79 | -8.43 | 1.53 | -5.51 | - | WB present | 0 | 3 | *Firmicutes* | *Clostridia* | *Clostridiales* | *Ruminococcaceae* | *-* | *-* |
| OTU 18 | 1646.50 | -4.05 | 1.59 | -2.56 | - | WB absent | 4 | 3 | *Firmicutes* | *Clostridia* | *Clostridiales* | *Veillonellaceae* | *Phascolarctobacterium* | *-* |
| OTU 19 | 25092.18 | 0.80 | 0.86 | 0.92 | 0.433 | WB absent | 5 | 5 | *Firmicutes* | *Bacilli* | *Lactobacillales* | *Aerococcaceae* | *Aerococcus* | *-* |
| OTU 20 | 1481.12 | -2.32 | 1.56 | -1.49 | - | WB absent | 5 | 3 | *Synergistetes* | *Synergistia* | *Synergistales* | *Synergistaceae* | *-* | *-* |
| OTU 21 | 503.90 | -7.94 | 1.37 | -5.80 | **< 0.001** | WB present | 1 | 5 | *Firmicutes* | *Clostridia* | *Clostridiales* | *[Tissierellaceae]* | *Peptoniphilus* | *-* |
| OTU 22 | 473.18 | -4.04 | 1.58 | -2.56 | - | WB absent | 3 | 2 | *Firmicutes* | *Clostridia* | *Clostridiales* | *Lachnospiraceae* | *-* | *-* |
| OTU 23 | 518.24 | -4.82 | 1.56 | -3.09 | - | WB present | 2 | 4 | *Bacteroidetes* | *Bacteroidia* | *Bacteroidales* | *Bacteroidaceae* | *Bacteroides* | *-* |
| OTU 24 | 645.43 | -4.97 | 1.56 | -3.18 | - | WB present | 3 | 3 | *Proteobacteria* | *Gammaproteobacteria* | *Enterobacteriales* | *Enterobacteriaceae* | *-* | *-* |
| OTU 25 | 392.00 | -7.31 | 1.55 | -4.71 | - | WB present | 0 | 2 | *Firmicutes* | *Clostridia* | *Clostridiales* | *Clostridiaceae* | *Clostridium* | *-* |
| OTU 26 | 444.21 | 1.68 | 1.31 | 1.28 | 0.273 | WB absent | 5 | 5 | *Actinobacteria* | *Actinobacteria* | *Actinomycetales* | *Propionibacteriaceae* | *-* | *-* |
| OTU 27 | 260.40 | -5.10 | 1.53 | -3.34 | - | WB present | 1 | 3 | *Firmicutes* | *Clostridia* | *Clostridiales* | *Lachnospiraceae* | *-* | *-* |
| OTU 28 | 367.85 | -4.19 | 1.51 | -2.77 | - | WB present | 3 | 3 | *Firmicutes* | *Clostridia* | *Clostridiales* | *Clostridiaceae* | *Clostridium* | *-* |
| OTU 29 | 370.15 | -7.55 | 1.38 | -5.49 | - | WB present | 1 | 4 | *Firmicutes* | *Clostridia* | *Clostridiales* | *[Tissierellaceae]* | *Gallicola* | *-* |
| OTU 30 | 280.22 | -6.84 | 1.52 | -4.49 | - | WB present | 1 | 2 | *Bacteroidetes* | *Bacteroidia* | *Bacteroidales* | *Porphyromonadaceae* | *Dysgonomonas* | *-* |
| OTU 31 | 209.85 | -5.11 | 1.52 | -3.37 | - | WB present | 1 | 2 | *Firmicutes* | *Clostridia* | *Clostridiales* | *Ruminococcaceae* | *-* | *-* |
| OTU 32 | 368.93 | -3.03 | 1.53 | -1.99 | - | WB absent | 5 | 2 | *Fusobacteria* | *Fusobacteriia* | *Fusobacteriales* | *Fusobacteriaceae* | *Fusobacterium* | *-* |
| OTU 33 | 252.58 | -1.20 | 1.41 | -0.85 | - | WB absent | 5 | 3 | *Proteobacteria* | *Deltaproteobacteria* | *Desulfovibrionales* | *Desulfovibrionaceae* | *Desulfovibrio* | *-* |
| OTU 34 | 225.96 | -4.14 | 1.46 | -2.83 | - | WB present | 4 | 3 | *Verrucomicrobia* | *Verrucomicrobiae* | *Verrucomicrobiales* | *Verrucomicrobiaceae* | *Akkermansia* | *muciniphila* |
| OTU 35 | 225.72 | -5.58 | 1.53 | -3.65 | - | WB present | 1 | 1 | *Firmicutes* | *Clostridia* | *Clostridiales* | *Clostridiaceae* | *Clostridium* | *-* |
| OTU 36 | 453.43 | -1.94 | 1.62 | -1.20 | - | WB absent | 3 | 3 | *Proteobacteria* | *Gammaproteobacteria* | *Pasteurellales* | *Pasteurellaceae* | *Lonepinella* | *koalarum* |
| OTU 37 | 184.58 | 2.00 | 1.21 | 1.65 | 0.165 | WB absent | 5 | 4 | *Proteobacteria* | *Alphaproteobacteria* | *Rhizobiales* | *Methylobacteriaceae* | *Methylobacterium* | *-* |
| OTU 38 | 214.73 | -7.38 | 1.23 | -6.01 | **< 0.001** | WB present | 0 | 5 | *Firmicutes* | *Clostridia* | *Clostridiales* | *[Tissierellaceae]* | *Peptoniphilus* | *-* |
| OTU 39 | 241.01 | -0.63 | 1.50 | -0.42 | - | WB absent | 5 | 2 | *Firmicutes* | *Clostridia* | *Clostridiales* | *Ruminococcaceae* | *Ruminococcus* | *-* |
| OTU 40 | 257.43 | -6.17 | 1.52 | -4.05 | - | WB present | 2 | 1 | *Bacteroidetes* | *Bacteroidia* | *Bacteroidales* | *Bacteroidaceae* | *Bacteroides* | *-* |
| OTU 41 | 285.26 | -1.67 | 1.54 | -1.08 | - | WB absent | 4 | 3 | *Firmicutes* | *Clostridia* | *Clostridiales* | *Ruminococcaceae* | *-* | *-* |
| OTU 42 | 348.71 | -7.24 | 1.54 | -4.71 | - | WB present | 0 | 3 | *Firmicutes* | *Clostridia* | *Clostridiales* | *Peptococcaceae* | *Peptococcus* | *-* |
| OTU 43 | 216.18 | -5.00 | 1.54 | -3.26 | - | WB present | 1 | 1 | *Planctomycetes* | *vadinHA49* | *PeHg47* | *-* | *-* | *-* |
| OTU 44 | 144.17 | -4.06 | 1.48 | -2.75 | - | WB present | 2 | 2 | *Bacteroidetes* | *Bacteroidia* | *Bacteroidales* | *Porphyromonadaceae* | *Parabacteroides* | *-* |
| OTU 45 | 313.21 | 1.93 | 1.26 | 1.53 | 0.202 | WB absent | 5 | 5 | *Proteobacteria* | *Alphaproteobacteria* | *Rhizobiales* | *Methylobacteriaceae* | *Methylobacterium* | *-* |
| OTU 46 | 104.25 | -3.26 | 1.42 | -2.30 | - | WB absent | 4 | 2 | *Proteobacteria* | *Deltaproteobacteria* | *Desulfovibrionales* | *Desulfovibrionaceae* | *-* | *-* |
| OTU 47 | 83.56 | -6.04 | 1.31 | -4.60 | **< 0.001** | WB present | 0 | 3 | *Firmicutes* | *Clostridia* | *Clostridiales* | *[Tissierellaceae]* | *ph2* | *-* |
| OTU 48 | 55.83 | -5.48 | 1.35 | -4.06 | - | WB present | 0 | 3 | *Synergistetes* | *Synergistia* | *Synergistales* | *Synergistaceae* | *vadinCA02* | *-* |
| OTU 49 | 78.07 | -5.77 | 1.43 | -4.04 | - | WB present | 0 | 2 | *Firmicutes* | *Clostridia* | *Clostridiales* | *-* | *-* | *-* |
| OTU 50 | 78.75 | -5.75 | 1.44 | -4.00 | - | WB present | 0 | 1 | *Actinobacteria* | *Actinobacteria* | *Actinomycetales* | *Actinomycetaceae* | *Mobiluncus* | *-* |
| OTU 51 | 48.47 | -5.39 | 1.28 | -4.20 | **< 0.001** | WB present | 0 | 3 | *Firmicutes* | *Clostridia* | *Clostridiales* | *[Tissierellaceae]* | *Peptoniphilus* | *-* |
| OTU 52 | 62.20 | -5.49 | 1.42 | -3.85 | - | WB present | 0 | 1 | *Firmicutes* | *Clostridia* | *Clostridiales* | *Veillonellaceae* | *Dialister* | *-* |
| OTU 53 | 55.98 | -5.37 | 1.42 | -3.79 | - | WB present | 0 | 1 | *Firmicutes* | *Clostridia* | *Clostridiales* | *[Tissierellaceae]* | *ph2* | *-* |
| OTU 54 | 38.95 | -4.99 | 1.38 | -3.62 | - | WB present | 0 | 2 | *Bacteroidetes* | *Bacteroidia* | *Bacteroidales* | *Bacteroidaceae* | *Bacteroides* | *-* |
| OTU 55 | 38.30 | -4.95 | 1.39 | -3.56 | - | WB present | 0 | 1 | *Bacteroidetes* | *Bacteroidia* | *Bacteroidales* | *Porphyromonadaceae* | *Porphyromonas* | *-* |
| OTU 56 | 36.05 | 1.33 | 1.38 | 0.96 | - | WB absent | 2 | 2 | *Firmicutes* | *Clostridia* | *Clostridiales* | *-* | *-* | *-* |
| OTU 57 | 24.55 | 2.17 | 1.13 | 1.92 | 0.099 | WB absent | 4 | 3 | *Proteobacteria* | *Alphaproteobacteria* | *Rhizobiales* | *Methylobacteriaceae* | *-* | *-* |
| OTU 58 | 32.22 | -4.75 | 1.38 | -3.46 | - | WB present | 0 | 1 | *Firmicutes* | *Clostridia* | *Clostridiales* | *Clostridiaceae* | *Clostridium* | *-* |
| OTU 59 | 36.44 | -1.73 | 1.36 | -1.27 | - | WB absent | 2 | 2 | *Firmicutes* | *Clostridia* | *Clostridiales* | *Lachnospiraceae* | *-* | *-* |
| OTU 60 | 43.08 | 5.07 | 1.40 | 3.62 | - | WB absent | 1 | 0 | *Firmicutes* | *Bacilli* | *Lactobacillales* | *Streptococcaceae* | *Streptococcus* | *-* |
| OTU 61 | 22.31 | 3.79 | 1.21 | 3.14 | - | WB absent | 4 | 1 | *Firmicutes* | *Clostridia* | *Clostridiales* | *Ruminococcaceae* | *-* | *-* |
| OTU 62 | 29.98 | -0.30 | 1.23 | -0.25 | 0.852 | WB absent | 4 | 2 | *Proteobacteria* | *Gammaproteobacteria* | *Pseudomonadales* | *Moraxellaceae* | *Acinetobacter* | *rhizosphaerae* |
| OTU 63 | 47.59 | 1.93 | 1.39 | 1.39 | 0.236 | WB absent | 3 | 1 | *Firmicutes* | *Clostridia* | *Clostridiales* | *Ruminococcaceae* | *-* | *-* |
| OTU 64 | 29.51 | -4.68 | 1.35 | -3.48 | - | WB present | 0 | 2 | *Firmicutes* | *Clostridia* | *Clostridiales* | *Ruminococcaceae* | *-* | *-* |
| OTU 65 | 67.82 | -5.47 | 1.36 | -4.02 | **0.001** | WB present | 1 | 2 | *Proteobacteria* | *Betaproteobacteria* | *Burkholderiales* | *Alcaligenaceae* | *Sutterella* | *-* |
| OTU 66 | 19.64 | -4.19 | 1.32 | -3.18 | - | WB present | 0 | 1 | *Firmicutes* | *Clostridia* | *Clostridiales* | *[Tissierellaceae]* | *ph2* | *-* |
| OTU 67 | 39.01 | -4.97 | 1.39 | -3.57 | - | WB present | 0 | 1 | *Bacteroidetes* | *Bacteroidia* | *Bacteroidales* | *Prevotellaceae* | *Prevotella* | *-* |
| OTU 69 | 18.56 | 4.18 | 1.24 | 3.37 | **0.005** | WB absent | 2 | 0 | *Firmicutes* | *Clostridia* | *Clostridiales* | *Lachnospiraceae* | *-* | *-* |
| OTU 70 | 15.48 | -3.91 | 1.28 | -3.05 | - | WB present | 0 | 1 | *Firmicutes* | *Clostridia* | *Clostridiales* | *Ruminococcaceae* | *Oscillospira* | *-* |
| OTU 71 | 21.40 | 4.34 | 1.27 | 3.42 | **0.005** | WB absent | 2 | 0 | *Firmicutes* | *Clostridia* | *Clostridiales* | *-* | *-* | *-* |
| OTU 72 | 20.34 | -4.23 | 1.32 | -3.21 | - | WB present | 0 | 1 | *Firmicutes* | *Clostridia* | *Clostridiales* | *Ruminococcaceae* | *-* | *-* |
| OTU 73 | 21.03 | -4.33 | 1.27 | -3.41 | **0.005** | WB present | 0 | 2 | *Bacteroidetes* | *Bacteroidia* | *Bacteroidales* | *Rikenellaceae* | *-* | *-* |
| OTU 74 | 12.93 | 3.67 | 1.26 | 2.91 | - | WB absent | 1 | 0 | *Firmicutes* | *Clostridia* | *Clostridiales* | *Ruminococcaceae* | *-* | *-* |
| OTU 75 | 27.32 | 4.62 | 1.30 | 3.56 | **0.004** | WB absent | 2 | 0 | *Firmicutes* | *Clostridia* | *Clostridiales* | *Lachnospiraceae* | *-* | *-* |
| OTU 76 | 19.92 | 1.34 | 1.19 | 1.12 | 0.335 | WB absent | 4 | 3 | *Proteobacteria* | *Gammaproteobacteria* | *Pseudomonadales* | *Pseudomonadaceae* | *Pseudomonas* | *-* |
| OTU 77 | 23.15 | 0.19 | 1.11 | 0.17 | 0.886 | WB absent | 5 | 3 | *Proteobacteria* | *Alphaproteobacteria* | *Sphingomonadales* | *Sphingomonadaceae* | *Sphingomonas* | *yabuuchiae* |
| OTU 78 | 26.20 | 3.76 | 1.18 | 3.17 | - | WB absent | 5 | 4 | *Firmicutes* | *Bacilli* | *Bacillales* | *Staphylococcaceae* | *Staphylococcus* | *-* |
| OTU 79 | 13.90 | -2.74 | 1.22 | -2.24 | - | WB present | 1 | 2 | *Proteobacteria* | *Deltaproteobacteria* | *Desulfovibrionales* | *Desulfovibrionaceae* | *-* | *-* |
| OTU 80 | 11.75 | 3.55 | 1.25 | 2.83 | - | WB absent | 1 | 0 | *Proteobacteria* | *Alphaproteobacteria* | *Rhizobiales* | *Hyphomicrobiaceae* | *Pedomicrobium* | *-* |
| OTU 81 | 14.51 | -3.84 | 1.27 | -3.01 | - | WB present | 0 | 1 | *Proteobacteria* | *Betaproteobacteria* | *Burkholderiales* | *Oxalobacteraceae* | *-* | *-* |
| OTU 82 | 12.93 | -3.69 | 1.26 | -2.93 | - | WB present | 0 | 1 | *Proteobacteria* | *Deltaproteobacteria* | *Desulfovibrionales* | *Desulfovibrionaceae* | *-* | *-* |
| OTU 83 | 13.59 | -3.52 | 1.24 | -2.84 | - | WB present | 1 | 2 | *Bacteroidetes* | *Bacteroidia* | *Bacteroidales* | *Bacteroidaceae* | *Bacteroides* | *fragilis* |
| OTU 84 | 12.10 | 3.59 | 1.26 | 2.85 | - | WB absent | 1 | 0 | *Firmicutes* | *Clostridia* | *Clostridiales* | *-* | *-* | *-* |
| OTU 86 | 20.70 | 4.37 | 1.18 | 3.71 | **0.003** | WB absent | 3 | 0 | *Bacteroidetes* | *Bacteroidia* | *Bacteroidales* | *Bacteroidaceae* | *Bacteroides* | *-* |
| OTU 87 | 10.18 | 3.36 | 1.24 | 2.72 | - | WB absent | 1 | 0 | *Firmicutes* | *Clostridia* | *Clostridiales* | *Lachnospiraceae* | *-* | *-* |
| OTU 88 | 11.83 | 3.55 | 1.26 | 2.82 | - | WB absent | 1 | 0 | *Firmicutes* | *Clostridia* | *Clostridiales* | *Ruminococcaceae* | *-* | *-* |
| OTU 89 | 7.42 | 2.95 | 1.19 | 2.48 | - | WB absent | 1 | 0 | *Firmicutes* | *Clostridia* | *Clostridiales* | *-* | *-* | *-* |
| OTU 90 | 24.08 | -3.05 | 1.27 | -2.40 | **0.048** | WB present | 1 | 2 | *Actinobacteria* | *Coriobacteriia* | *Coriobacteriales* | *Coriobacteriaceae* | *-* | *-* |
| OTU 91 | 19.98 | -4.21 | 1.32 | -3.20 | - | WB present | 0 | 1 | *Proteobacteria* | *Deltaproteobacteria* | *Desulfarculales* | *Desulfarculaceae* | *-* | *-* |
| OTU 92 | 12.44 | 1.48 | 1.13 | 1.31 | 0.265 | WB absent | 3 | 2 | *Proteobacteria* | *Betaproteobacteria* | *Burkholderiales* | *Comamonadaceae* | *Comamonas* | *-* |
| OTU 93 | 19.03 | -4.17 | 1.30 | -3.22 | - | WB present | 0 | 2 | *Bacteroidetes* | *Bacteroidia* | *Bacteroidales* | *Bacteroidaceae* | *Bacteroides* | *-* |
| OTU 94 | 20.71 | 4.06 | 1.26 | 3.22 | **0.007** | WB absent | 2 | 1 | *Proteobacteria* | *Alphaproteobacteria* | *Rhizobiales* | *Methylocystaceae* | *-* | *-* |
| OTU 95 | 8.91 | 3.23 | 1.16 | 2.79 | **0.022** | WB absent | 2 | 0 | *Proteobacteria* | *Alphaproteobacteria* | *Rhizobiales* | *Rhizobiaceae* | *Rhizobium* | *leguminosarum* |
| OTU 96 | 33.02 | 4.56 | 1.36 | 3.36 | - | WB absent | 2 | 1 | *Firmicutes* | *Clostridia* | *Clostridiales* | *-* | *-* | *-* |
| OTU 97 | 12.21 | 1.53 | 1.12 | 1.37 | - | WB absent | 5 | 2 | *Proteobacteria* | *Betaproteobacteria* | *Burkholderiales* | *Comamonadaceae* | *-* | *-* |
| OTU 98 | 18.54 | -4.14 | 1.29 | -3.20 | - | WB present | 0 | 2 | *Planctomycetes* | *vadinHA49* | *PeHg47* | *-* | *-* | *-* |
| OTU 99 | 11.43 | -3.53 | 1.25 | -2.82 | - | WB present | 0 | 1 | *Actinobacteria* | *Coriobacteriia* | *Coriobacteriales* | *Coriobacteriaceae* | *-* | *-* |
| OTU 100 | 10.44 | -3.41 | 1.24 | -2.75 | - | WB present | 0 | 1 | *Firmicutes* | *Clostridia* | *Clostridiales* | *[Mogibacteriaceae]* | *Mogibacterium* | *-* |
| OTU 101 | 8.05 | 0.05 | 1.02 | 0.05 | 0.962 | WB absent | 3 | 3 | *Bacteroidetes* | *[Saprospirae]* | *[Saprospirales]* | *Chitinophagaceae* | *Sediminibacterium* | *-* |
| OTU 102 | 9.07 | 2.56 | 1.15 | 2.24 | - | WB absent | 3 | 2 | *Firmicutes* | *Clostridia* | *Clostridiales* | *Lachnospiraceae* | *Blautia* | *producta* |
| OTU 103 | 9.30 | 3.29 | 1.17 | 2.81 | **0.022** | WB absent | 2 | 0 | Unassigned | *-* | *-* | *-* | *-* | *-* |
| OTU 105 | 159.80 | -2.77 | 1.13 | -2.46 | **0.045** | WB present | 4 | 5 | *Firmicutes* | *Bacilli* | *Lactobacillales* | *Aerococcaceae* | *Aerococcus* | *-* |
| OTU 106 | 9.01 | 3.25 | 1.14 | 2.85 | **0.022** | WB absent | 3 | 0 | *Proteobacteria* | *Betaproteobacteria* | *Burkholderiales* | *Burkholderiaceae* | *Burkholderia* | *-* |
| OTU 107 | 9.65 | 3.30 | 1.23 | 2.67 | - | WB absent | 1 | 0 | *Proteobacteria* | *Alphaproteobacteria* | *Sphingomonadales* | *Sphingomonadaceae* | *Sphingobium* | *-* |
| OTU 108 | 6.29 | 2.01 | 1.12 | 1.79 | - | WB absent | 1 | 1 | *Firmicutes* | *Clostridia* | *Clostridiales* | *-* | *-* | *-* |
| OTU 109 | 8.93 | -3.24 | 1.16 | -2.79 | **0.022** | WB present | 0 | 2 | *Firmicutes* | *Clostridia* | *Clostridiales* | *Peptostreptococcaceae* | *Peptostreptococcus* | *-* |
| OTU 110 | 4.67 | -2.35 | 1.08 | -2.17 | - | WB present | 0 | 1 | *Acidobacteria* | *Acidobacteriia* | *Acidobacteriales* | *Acidobacteriaceae* | *-* | *-* |
| OTU 111 | 6.63 | -2.11 | 1.10 | -1.93 | - | WB present | 1 | 3 | *Firmicutes* | *Clostridia* | *Clostridiales* | *Ruminococcaceae* | *-* | *-* |
| OTU 112 | 5.59 | 2.59 | 1.10 | 2.35 | - | WB absent | 2 | 0 | *Actinobacteria* | *Actinobacteria* | *Actinomycetales* | *Nocardiaceae* | *Rhodococcus* | *fascians* |
| OTU 113 | 5.19 | 2.51 | 1.00 | 2.50 | **0.044** | WB absent | 3 | 0 | *Proteobacteria* | *Alphaproteobacteria* | *Rhizobiales* | *Rhizobiaceae* | *Agrobacterium* | *-* |
| OTU 114 | 8.21 | 2.86 | 1.14 | 2.52 | **0.043** | WB absent | 2 | 1 | *Proteobacteria* | *Betaproteobacteria* | *Burkholderiales* | *Oxalobacteraceae* | *-* | *-* |
| OTU 115 | 7.96 | -1.42 | 1.14 | -1.24 | - | WB absent | 2 | 1 | *Cyanobacteria* | *4C0d-2* | *YS2* | *-* | *-* | *-* |
| OTU 116 | 6.13 | -2.73 | 1.15 | -2.37 | - | WB present | 0 | 1 | *Firmicutes* | *Clostridia* | *Clostridiales* | *Ruminococcaceae* | *-* | *-* |
| OTU 117 | 5.28 | -2.52 | 1.11 | -2.27 | - | WB present | 0 | 1 | *Proteobacteria* | *Deltaproteobacteria* | *Desulfobacterales* | *Desulfobacteraceae* | *-* | *-* |
| OTU 118 | 5.77 | -2.64 | 1.13 | -2.33 | - | WB present | 0 | 1 | *Bacteroidetes* | *Bacteroidia* | *Bacteroidales* | *Porphyromonadaceae* | *Parabacteroides* | *-* |
| OTU 119 | 5.40 | 1.20 | 1.04 | 1.15 | 0.331 | WB absent | 2 | 1 | *Proteobacteria* | *Betaproteobacteria* | *Burkholderiales* | *Oxalobacteraceae* | *Ralstonia* | *-* |
| OTU 120 | 7.55 | 2.97 | 1.19 | 2.49 | - | WB absent | 1 | 0 | *Bacteroidetes* | *Bacteroidia* | *Bacteroidales* | *[Paraprevotellaceae]* | *Paraprevotella* | *-* |
| OTU 121 | 6.24 | 2.72 | 1.15 | 2.36 | - | WB absent | 1 | 0 | *Firmicutes* | *Erysipelotrichi* | *Erysipelotrichales* | *Erysipelotrichaceae* | *Coprobacillus* | *-* |
| OTU 122 | 9.13 | 3.22 | 1.22 | 2.63 | - | WB absent | 1 | 0 | *Actinobacteria* | *Actinobacteria* | *Actinomycetales* | *Corynebacteriaceae* | *Corynebacterium* | *-* |
| OTU 123 | 18.77 | -2.02 | 1.14 | -1.76 | 0.134 | WB present | 2 | 5 | Unassigned | *-* | *-* | *-* | *-* | *-* |
| OTU 124 | 5.82 | 2.63 | 1.14 | 2.30 | - | WB absent | 1 | 0 | *Actinobacteria* | *Actinobacteria* | *Actinomycetales* | *Micrococcaceae* | *Rothia* | *mucilaginosa* |
| OTU 125 | 8.62 | 3.15 | 1.22 | 2.59 | - | WB absent | 1 | 0 | *Proteobacteria* | *Deltaproteobacteria* | *Desulfarculales* | *Desulfarculaceae* | *-* | *-* |
| OTU 126 | 7.58 | 2.73 | 1.16 | 2.35 | - | WB absent | 2 | 1 | *Firmicutes* | *Clostridia* | *Clostridiales* | *Lachnospiraceae* | *-* | *-* |
| OTU 127 | 4.72 | -2.38 | 1.06 | -2.25 | - | WB present | 0 | 2 | *Firmicutes* | *Clostridia* | *Clostridiales* | *Lachnospiraceae* | *-* | *-* |
| OTU 128 | 7.83 | -3.05 | 1.20 | -2.55 | - | WB present | 0 | 1 | *Planctomycetes* | *vadinHA49* | *PeHg47* | *-* | *-* | *-* |
| OTU 129 | 3.27 | 1.77 | 0.98 | 1.80 | - | WB absent | 1 | 0 | *Actinobacteria* | *Actinobacteria* | *Actinomycetales* | *Micrococcaceae* | *-* | *-* |
| OTU 130 | 5.09 | 2.16 | 1.07 | 2.02 | - | WB absent | 2 | 1 | *Bacteroidetes* | *Bacteroidia* | *Bacteroidales* | *Bacteroidaceae* | *Bacteroides* | *-* |
| OTU 131 | 6.28 | 2.02 | 1.04 | 1.94 | 0.097 | WB absent | 2 | 2 | *Firmicutes* | *Clostridia* | *Clostridiales* | *-* | *-* | *-* |
| OTU 132 | 8.90 | 0.65 | 1.09 | 0.59 | 0.630 | WB absent | 3 | 3 | *Proteobacteria* | *Betaproteobacteria* | *Burkholderiales* | *Comamonadaceae* | *-* | *-* |
| OTU 133 | 4.47 | 2.25 | 1.07 | 2.10 | - | WB absent | 1 | 0 | *Bacteroidetes* | *Cytophagia* | *Cytophagales* | *Cytophagaceae* | *Hymenobacter* | *-* |
| OTU 134 | 4.67 | 2.32 | 1.08 | 2.14 | - | WB absent | 1 | 0 | *Firmicutes* | *Clostridia* | *Clostridiales* | *Eubacteriaceae* | *Anaerofustis* | *-* |
| OTU 135 | 3.94 | -2.07 | 1.04 | -2.00 | 0.092 | WB present | 0 | 1 | *Bacteroidetes* | *Bacteroidia* | *Bacteroidales* | *Bacteroidaceae* | *Bacteroides* | *uniformis* |
| OTU 136 | 4.45 | 1.46 | 1.02 | 1.42 | 0.232 | WB absent | 1 | 1 | *Bacteroidetes* | *Bacteroidia* | *Bacteroidales* | *Bacteroidaceae* | *Bacteroides* | *-* |
| OTU 137 | 8.75 | 1.01 | 1.08 | 0.93 | 0.433 | WB absent | 3 | 1 | *Actinobacteria* | *Actinobacteria* | *Actinomycetales* | *Corynebacteriaceae* | *Corynebacterium* | *-* |
| OTU 138 | 3.93 | 2.03 | 1.04 | 1.96 | 0.096 | WB absent | 1 | 0 | *Firmicutes* | *Clostridia* | *Clostridiales* | *Ruminococcaceae* | *Oscillospira* | *-* |
| OTU 139 | 3.75 | 1.99 | 1.02 | 1.95 | - | WB absent | 1 | 0 | *Actinobacteria* | *Actinobacteria* | *Actinomycetales* | *Micrococcaceae* | *Rothia* | *dentocariosa* |
| OTU 140 | 4.85 | -0.30 | 1.00 | -0.30 | 0.842 | WB present | 1 | 2 | *Proteobacteria* | *Gammaproteobacteria* | *Pseudomonadales* | *Moraxellaceae* | *Acinetobacter* | *-* |
| OTU 141 | 4.87 | 1.85 | 1.02 | 1.81 | 0.124 | WB absent | 2 | 1 | *Actinobacteria* | *Thermoleophilia* | *Solirubrobacterales* | *-* | *-* | *-* |
| OTU 142 | 5.61 | -2.58 | 1.13 | -2.28 | - | WB present | 0 | 1 | *Firmicutes* | *Clostridia* | *Clostridiales* | *-* | *-* | *-* |
| OTU 143 | 3.10 | 1.69 | 0.97 | 1.74 | - | WB absent | 1 | 0 | *Bacteroidetes* | *Bacteroidia* | *Bacteroidales* | *Bacteroidaceae* | *Bacteroides* | *ovatus* |
| OTU 144 | 5.36 | 2.52 | 1.08 | 2.35 | 0.054 | WB absent | 2 | 0 | *Actinobacteria* | *Actinobacteria* | *Actinomycetales* | *Corynebacteriaceae* | *Corynebacterium* | *-* |
| OTU 145 | 3.46 | -1.90 | 1.00 | -1.91 | - | WB present | 0 | 1 | *Firmicutes* | *Clostridia* | *Clostridiales* | *Clostridiaceae* | *Clostridium* | *-* |
| OTU 146 | 5.01 | 2.44 | 1.05 | 2.33 | 0.054 | WB absent | 2 | 0 | *Bacteroidetes* | *Bacteroidia* | *Bacteroidales* | *Prevotellaceae* | *Prevotella* | *-* |
| OTU 147 | 4.19 | -2.19 | 1.05 | -2.08 | - | WB present | 0 | 1 | Unassigned | *-* | *-* | *-* | *-* | *-* |
| OTU 148 | 8.75 | -3.22 | 1.15 | -2.79 | **0.022** | WB present | 0 | 2 | Unassigned | *-* | *-* | *-* | *-* | *-* |
| OTU 149 | 3.43 | 0.04 | 0.92 | 0.05 | - | WB present | 1 | 1 | *Proteobacteria* | *Deltaproteobacteria* | *Desulfovibrionales* | *Desulfovibrionaceae* | *Desulfovibrio* | *-* |
| OTU 150 | 5.94 | 2.65 | 1.14 | 2.32 | - | WB absent | 1 | 0 | *Bacteroidetes* | *Cytophagia* | *Cytophagales* | *Cytophagaceae* | *Hymenobacter* | *-* |
| OTU 151 | 4.90 | 2.40 | 1.06 | 2.26 | 0.061 | WB absent | 2 | 0 | *Actinobacteria* | *Actinobacteria* | *Actinomycetales* | *Kineosporiaceae* | *-* | *-* |
| OTU 152 | 5.16 | -2.49 | 1.11 | -2.25 | - | WB present | 0 | 1 | *Proteobacteria* | *Deltaproteobacteria* | *Desulfovibrionales* | *Desulfovibrionaceae* | *-* | *-* |
| OTU 153 | 4.01 | -1.11 | 0.97 | -1.15 | 0.331 | WB present | 2 | 1 | *Proteobacteria* | *Deltaproteobacteria* | *Desulfovibrionales* | *Desulfovibrionaceae* | *Bilophila* | *-* |
| OTU 154 | 3.27 | 1.77 | 0.98 | 1.80 | - | WB absent | 1 | 0 | *Proteobacteria* | *Deltaproteobacteria* | *Desulfovibrionales* | *Desulfovibrionaceae* | *-* | *-* |
| OTU 155 | 104.48 | -1.49 | 1.32 | -1.13 | 0.335 | WB absent | 4 | 3 | *Firmicutes* | *Bacilli* | *Lactobacillales* | *Carnobacteriaceae* | *Trichococcus* | *-* |
| OTU 156 | 3.79 | 2.01 | 0.96 | 2.09 | - | WB absent | 3 | 0 | *Armatimonadetes* | *[Fimbriimonadia]* | *[Fimbriimonadales]* | *[Fimbriimonadaceae]* | *Fimbriimonas* | *-* |
| OTU 157 | 3.80 | -1.00 | 0.88 | -1.14 | - | WB present | 1 | 4 | *Firmicutes* | *Clostridia* | *Clostridiales* | *Clostridiaceae* | *Clostridium* | *-* |
| OTU 158 | 2.97 | 1.59 | 0.96 | 1.66 | - | WB absent | 1 | 0 | *Proteobacteria* | *Alphaproteobacteria* | *Rhizobiales* | *Methylocystaceae* | *-* | *-* |
| OTU 159 | 11.73 | -2.84 | 1.08 | -2.62 | **0.035** | WB present | 2 | 4 | Unassigned | *-* | *-* | *-* | *-* | *-* |
| OTU 160 | 3.12 | -1.41 | 0.94 | -1.50 | - | WB present | 1 | 1 | *Firmicutes* | *Clostridia* | *Clostridiales* | *-* | *-* | *-* |
| OTU 161 | 4.54 | 1.49 | 1.00 | 1.48 | 0.214 | WB absent | 2 | 2 | *Firmicutes* | *Clostridia* | *Clostridiales* | *Clostridiaceae* | *Clostridium* | *perfringens* |
| OTU 162 | 3.11 | 1.70 | 0.95 | 1.78 | - | WB absent | 2 | 0 | *Firmicutes* | *Clostridia* | *Clostridiales* | *Ruminococcaceae* | *-* | *-* |
| OTU 163 | 3.84 | 2.04 | 0.99 | 2.06 | 0.081 | WB absent | 2 | 0 | *Proteobacteria* | *Gammaproteobacteria* | *Oceanospirillales* | *Halomonadaceae* | *-* | *-* |
| OTU 164 | 3.36 | -1.86 | 0.97 | -1.92 | - | WB present | 0 | 2 | *Proteobacteria* | *Gammaproteobacteria* | *Pseudomonadales* | *Moraxellaceae* | *Acinetobacter* | *-* |
| OTU 165 | 22.27 | -0.89 | 1.07 | -0.83 | 0.486 | WB present | 4 | 4 | Unassigned | *-* | *-* | *-* | *-* | *-* |
| OTU 166 | 4.50 | -2.27 | 1.08 | -2.11 | - | WB present | 0 | 1 | *Firmicutes* | *Clostridia* | *Clostridiales* | *Lachnospiraceae* | *-* | *-* |
| OTU 167 | 4.12 | -2.18 | 1.02 | -2.14 | 0.071 | WB present | 0 | 2 | *Firmicutes* | *Clostridia* | *Clostridiales* | *Lachnospiraceae* | *Dorea* | *-* |
| OTU 168 | 3.10 | 1.69 | 0.97 | 1.74 | - | WB absent | 1 | 0 | *Firmicutes* | *Clostridia* | *Clostridiales* | *Ruminococcaceae* | *Oscillospira* | *-* |
| OTU 169 | 3.51 | -1.89 | 1.01 | -1.88 | - | WB present | 0 | 1 | *Actinobacteria* | *Actinobacteria* | *Actinomycetales* | *Intrasporangiaceae* | *Terracoccus* | *-* |
| OTU 170 | 4.40 | 2.23 | 1.02 | 2.18 | 0.068 | WB absent | 3 | 0 | *Firmicutes* | *Bacilli* | *Lactobacillales* | *Enterococcaceae* | *-* | *-* |
| OTU 171 | 2.71 | 1.46 | 0.91 | 1.62 | - | WB absent | 2 | 0 | *Firmicutes* | *Clostridia* | *Clostridiales* | *Lachnospiraceae* | *Blautia* | *-* |
| OTU 172 | 5.41 | 2.03 | 0.94 | 2.16 | 0.070 | WB absent | 4 | 1 | *Proteobacteria* | *Betaproteobacteria* | *Burkholderiales* | *Oxalobacteraceae* | *Ralstonia* | *-* |
| OTU 173 | 2.73 | -1.52 | 0.93 | -1.63 | - | WB present | 0 | 1 | *Deferribacteres* | *Deferribacteres* | *Deferribacterales* | *Deferribacteraceae* | *Mucispirillum* | *-* |
| OTU 174 | 6.05 | -0.70 | 1.07 | -0.66 | 0.592 | WB present | 1 | 2 | Unassigned | *-* | *-* | *-* | *-* | *-* |
| OTU 175 | 4.55 | 2.30 | 1.03 | 2.23 | 0.063 | WB absent | 2 | 0 | *Proteobacteria* | *Betaproteobacteria* | *Burkholderiales* | *Oxalobacteraceae* | *Oxalobacter* | *formigenes* |
| OTU 176 | 2.49 | -1.36 | 0.91 | -1.50 | - | WB present | 0 | 1 | *Firmicutes* | *Clostridia* | *Clostridiales* | *Lachnospiraceae* | *-* | *-* |
| OTU 177 | 4.67 | -2.35 | 1.08 | -2.17 | - | WB present | 0 | 1 | *Proteobacteria* | *Deltaproteobacteria* | *Desulfovibrionales* | *Desulfovibrionaceae* | *Bilophila* | *-* |
| OTU 178 | 3.36 | -1.54 | 0.96 | -1.60 | - | WB present | 1 | 1 | Unassigned | *-* | *-* | *-* | *-* | *-* |
| OTU 179 | 2.33 | 1.18 | 0.88 | 1.33 | - | WB absent | 2 | 0 | *Actinobacteria* | *Actinobacteria* | *Actinomycetales* | *Corynebacteriaceae* | *Corynebacterium* | *-* |
| OTU 180 | 2.33 | 1.17 | 0.90 | 1.31 | - | WB absent | 1 | 0 | *Proteobacteria* | *Alphaproteobacteria* | *Rhodospirillales* | *Rhodospirillaceae* | *-* | *-* |
| OTU 181 | 7.22 | -2.94 | 1.18 | -2.49 | - | WB present | 0 | 1 | *Bacteroidetes* | *Bacteroidia* | *Bacteroidales* | *Porphyromonadaceae* | *Parabacteroides* | *-* |
| OTU 182 | 2.33 | 1.17 | 0.90 | 1.31 | - | WB absent | 1 | 0 | *Cyanobacteria* | *4C0d-2* | *MLE1-12* | *-* | *-* | *-* |
| OTU 183 | 10.62 | -2.20 | 1.14 | -1.93 | - | WB present | 3 | 3 | *Proteobacteria* | *Gammaproteobacteria* | *Pseudomonadales* | *Pseudomonadaceae* | *Pseudomonas* | *-* |
| OTU 184 | 4.09 | 1.00 | 0.93 | 1.06 | 0.360 | WB absent | 2 | 1 | *Proteobacteria* | *Betaproteobacteria* | *Burkholderiales* | *Comamonadaceae* | *-* | *-* |
| OTU 185 | 2.20 | 1.06 | 0.88 | 1.20 | - | WB absent | 1 | 0 | *Proteobacteria* | *Alphaproteobacteria* | *Rhodospirillales* | *Acetobacteraceae* | *-* | *-* |
| OTU 186 | 2.24 | -1.10 | 0.89 | -1.24 | - | WB present | 0 | 1 | *Firmicutes* | *Clostridia* | *Clostridiales* | *[Tissierellaceae]* | *Peptoniphilus* | *-* |
| OTU 187 | 3.62 | 0.17 | 0.90 | 0.18 | - | WB absent | 2 | 1 | *Proteobacteria* | *Gammaproteobacteria* | *Pseudomonadales* | *Moraxellaceae* | *Enhydrobacter* | *-* |
| OTU 188 | 2.20 | 1.06 | 0.88 | 1.20 | - | WB absent | 1 | 0 | *Proteobacteria* | *Betaproteobacteria* | *Neisseriales* | *Neisseriaceae* | *Neisseria* | *subflava* |
| OTU 189 | 2.11 | 0.95 | 0.88 | 1.07 | - | WB absent | 1 | 0 | *Proteobacteria* | *Alphaproteobacteria* | *Rhodospirillales* | *Acetobacteraceae* | *-* | *-* |
| OTU 190 | 2.25 | 1.08 | 0.89 | 1.21 | - | WB absent | 1 | 0 | *Actinobacteria* | *Actinobacteria* | *Actinomycetales* | *Nocardioidaceae* | *-* | *-* |
| OTU 191 | 6.13 | -2.73 | 1.15 | -2.37 | - | WB present | 0 | 1 | *Planctomycetes* | *vadinHA49* | *PeHg47* | *-* | *-* | *-* |
| OTU 192 | 2.73 | -1.52 | 0.93 | -1.63 | - | WB present | 0 | 1 | *Bacteroidetes* | *Bacteroidia* | *Bacteroidales* | *Bacteroidaceae* | *Bacteroides* | *ovatus* |
| OTU 193 | 4.30 | 2.22 | 1.00 | 2.21 | 0.065 | WB absent | 2 | 0 | *Firmicutes* | *Clostridia* | *Clostridiales* | *Lachnospiraceae* | *-* | *-* |
| OTU 194 | 4.43 | -2.27 | 1.07 | -2.13 | - | WB present | 0 | 1 | *Bacteroidetes* | *Bacteroidia* | *Bacteroidales* | *Rikenellaceae* | *-* | *-* |
| OTU 195 | 3.81 | 2.01 | 1.03 | 1.95 | 0.096 | WB absent | 1 | 0 | *Firmicutes* | *Clostridia* | *Clostridiales* | *Ruminococcaceae* | *-* | *-* |
| OTU 196 | 4.23 | -0.22 | 0.83 | -0.26 | 0.852 | WB present | 4 | 5 | *Proteobacteria* | *Gammaproteobacteria* | *Enterobacteriales* | *Enterobacteriaceae* | *Plesiomonas* | *shigelloides* |
| OTU 197 | 1.97 | 0.80 | 0.88 | 0.91 | - | WB absent | 1 | 0 | *Acidobacteria* | *Acidobacteriia* | *Acidobacteriales* | *Acidobacteriaceae* | *Terriglobus* | *-* |
| OTU 199 | 2.60 | 1.38 | 0.92 | 1.49 | - | WB absent | 1 | 0 | *Actinobacteria* | *Actinobacteria* | *Actinomycetales* | *Corynebacteriaceae* | *Corynebacterium* | *-* |
| OTU 200 | 14.70 | -1.45 | 1.22 | -1.19 | - | WB present | 2 | 2 | Unassigned | *-* | *-* | *-* | *-* | *-* |
| OTU 201 | 12.56 | -3.65 | 1.26 | -2.89 | - | WB present | 0 | 1 | *Firmicutes* | *Bacilli* | *Lactobacillales* | *Aerococcaceae* | *Aerococcus* | *-* |
| OTU 202 | 2.85 | -1.59 | 0.94 | -1.69 | - | WB present | 0 | 1 | *Firmicutes* | *Clostridia* | *Clostridiales* | *Lachnospiraceae* | *Coprococcus* | *-* |
| OTU 204 | 3.33 | 0.45 | 0.83 | 0.54 | - | WB absent | 3 | 3 | *Fusobacteria* | *Fusobacteriia* | *Fusobacteriales* | *Leptotrichiaceae* | *-* | *-* |
| OTU 205 | 2.05 | -0.17 | 0.84 | -0.20 | - | WB present | 1 | 1 | *Firmicutes* | *Clostridia* | *Clostridiales* | *Lachnospiraceae* | *-* | *-* |
| OTU 206 | 3.34 | -1.85 | 0.99 | -1.87 | - | WB present | 0 | 1 | *Proteobacteria* | *Gammaproteobacteria* | *Pasteurellales* | *Pasteurellaceae* | *Aggregatibacter* | *-* |
| OTU 207 | 2.12 | -1.06 | 0.88 | -1.20 | - | WB present | 0 | 1 | Unassigned | *-* | *-* | *-* | *-* | *-* |
| OTU 209 | 3.24 | 0.34 | 0.81 | 0.42 | - | WB absent | 4 | 4 | *Firmicutes* | *Clostridia* | *Clostridiales* | *Peptostreptococcaceae* | *-* | *-* |
| OTU 210 | 2.36 | -1.27 | 0.90 | -1.41 | - | WB present | 0 | 1 | *Bacteroidetes* | *Bacteroidia* | *Bacteroidales* | *Rikenellaceae* | *-* | *-* |
| OTU 212 | 2.57 | 1.37 | 0.92 | 1.49 | - | WB absent | 1 | 0 | *Firmicutes* | *Clostridia* | *Clostridiales* | *Dehalobacteriaceae* | *-* | *-* |
| OTU 213 | 6.68 | 0.24 | 1.12 | 0.21 | 0.867 | WB absent | 2 | 1 | *Firmicutes* | *Clostridia* | *Clostridiales* | *-* | *-* | *-* |
| OTU 214 | 2.12 | -1.06 | 0.88 | -1.20 | - | WB present | 0 | 1 | *Proteobacteria* | *Gammaproteobacteria* | *Pseudomonadales* | *Moraxellaceae* | *Acinetobacter* | *-* |
| OTU 215 | 2.99 | 1.63 | 0.92 | 1.78 | - | WB absent | 2 | 0 | *Firmicutes* | *Clostridia* | *Clostridiales* | *Veillonellaceae* | *Veillonella* | *dispar* |
| OTU 216 | 4.22 | -2.18 | 1.06 | -2.06 | 0.081 | WB present | 0 | 1 | Unassigned | *-* | *-* | *-* | *-* | *-* |
| OTU 217 | 2.33 | 1.17 | 0.90 | 1.31 | - | WB absent | 1 | 0 | *Proteobacteria* | *Alphaproteobacteria* | *Rhizobiales* | *Bradyrhizobiaceae* | *-* | *-* |
| OTU 218 | 2.84 | 0.26 | 0.86 | 0.31 | - | WB present | 1 | 2 | *Firmicutes* | *Bacilli* | *Bacillales* | *-* | *-* | *-* |
| OTU 219 | 2.12 | -0.98 | 0.88 | -1.11 | - | WB present | 0 | 1 | *Proteobacteria* | *Alphaproteobacteria* | *Rhodobacterales* | *Rhodobacteraceae* | *Amaricoccus* | *-* |
| OTU 220 | 8.18 | -1.49 | 1.16 | -1.28 | - | WB present | 2 | 1 | *Firmicutes* | *Clostridia* | *Clostridiales* | *Lachnospiraceae* | *-* | *-* |
| OTU 221 | 2.85 | -1.59 | 0.94 | -1.69 | - | WB present | 0 | 1 | Unassigned | *-* | *-* | *-* | *-* | *-* |
| OTU 222 | 3.15 | 1.69 | 0.94 | 1.79 | - | WB absent | 3 | 0 | Unassigned | *-* | *-* | *-* | *-* | *-* |
| OTU 223 | 4.14 | 1.35 | 0.95 | 1.42 | 0.232 | WB absent | 2 | 1 | *Firmicutes* | *Bacilli* | *Lactobacillales* | *Streptococcaceae* | *Streptococcus* | *-* |
| OTU 224 | 2.56 | -0.98 | 0.89 | -1.10 | - | WB present | 1 | 1 | *Proteobacteria* | *Alphaproteobacteria* | *Sphingomonadales* | *Sphingomonadaceae* | *-* | *-* |
| OTU 225 | 1.97 | 0.80 | 0.88 | 0.91 | - | WB absent | 1 | 0 | *Proteobacteria* | *Betaproteobacteria* | *Gallionellales* | *Gallionellaceae* | *Gallionella* | *-* |
| OTU 226 | 2.81 | -1.52 | 0.94 | -1.61 | - | WB present | 0 | 1 | Unassigned | *-* | *-* | *-* | *-* | *-* |
| OTU 227 | 71.09 | -5.66 | 1.42 | -3.98 | - | WB present | 0 | 2 | Unassigned | *-* | *-* | *-* | *-* | *-* |
| OTU 228 | 2.06 | 0.93 | 0.88 | 1.06 | - | WB absent | 1 | 0 | *Firmicutes* | *Clostridia* | *Clostridiales* | *Ruminococcaceae* | *Ruminococcus* | *-* |
| OTU 229 | 3.39 | 0.44 | 0.89 | 0.50 | - | WB absent | 2 | 1 | *Firmicutes* | *Clostridia* | *Clostridiales* | *Clostridiaceae* | *-* | *-* |
| OTU 230 | 1.90 | -0.39 | 0.85 | -0.46 | - | WB present | 1 | 1 | Unassigned | *-* | *-* | *-* | *-* | *-* |
| OTU 231 | 3.31 | 1.80 | 0.94 | 1.91 | - | WB absent | 2 | 0 | *Proteobacteria* | *Alphaproteobacteria* | *Rhizobiales* | *Methylocystaceae* | *-* | *-* |
| OTU 232 | 1.67 | -0.48 | 0.88 | -0.54 | - | WB present | 0 | 1 | *Proteobacteria* | *Alphaproteobacteria* | *Rhodospirillales* | *Acetobacteraceae* | *-* | *-* |
| OTU 233 | 1.92 | 0.00 | 0.84 | 0.00 | - | WB present | 1 | 1 | Unassigned | *-* | *-* | *-* | *-* | *-* |
| OTU 234 | 1.97 | 0.80 | 0.88 | 0.91 | - | WB absent | 1 | 0 | *Bacteroidetes* | *Bacteroidia* | *Bacteroidales* | *Bacteroidaceae* | *Bacteroides* | *-* |
| OTU 235 | 2.25 | 1.08 | 0.89 | 1.21 | - | WB absent | 1 | 0 | *Bacteroidetes* | *Bacteroidia* | *Bacteroidales* | *Bacteroidaceae* | *Bacteroides* | *-* |
| OTU 236 | 1.64 | -0.47 | 0.88 | -0.53 | - | WB present | 0 | 1 | *Bacteroidetes* | *Bacteroidia* | *Bacteroidales* | *Rikenellaceae* | *-* | *-* |
| OTU 237 | 1.97 | -0.83 | 0.88 | -0.95 | - | WB present | 0 | 1 | *Bacteroidetes* | *Bacteroidia* | *Bacteroidales* | *Bacteroidaceae* | *Bacteroides* | *-* |
| OTU 238 | 18.50 | -4.12 | 1.31 | -3.15 | - | WB present | 0 | 1 | Unassigned | *-* | *-* | *-* | *-* | *-* |
| OTU 239 | 2.39 | 0.48 | 0.84 | 0.58 | - | WB absent | 2 | 1 | *Fusobacteria* | *Fusobacteriia* | *Fusobacteriales* | *Fusobacteriaceae* | *Fusobacterium* | *-* |
| OTU 240 | 93.07 | -5.98 | 1.43 | -4.19 | - | WB present | 0 | 2 | *Bacteroidetes* | *Bacteroidia* | *Bacteroidales* | *Porphyromonadaceae* | *Parabacteroides* | *-* |
| OTU 241 | 2.20 | 1.06 | 0.88 | 1.20 | - | WB absent | 1 | 0 | *Firmicutes* | *Clostridia* | *Clostridiales* | *[Tissierellaceae]* | *Anaerococcus* | *-* |
| OTU 242 | 2.99 | 1.64 | 0.90 | 1.82 | - | WB absent | 3 | 0 | *Proteobacteria* | *Betaproteobacteria* | *Burkholderiales* | *Comamonadaceae* | *-* | *-* |
| OTU 243 | 3.24 | 0.86 | 0.83 | 1.03 | - | WB absent | 4 | 3 | *Fusobacteria* | *Fusobacteriia* | *Fusobacteriales* | *Fusobacteriaceae* | *Cetobacterium* | *somerae* |
| OTU 244 | 3.46 | -1.90 | 1.00 | -1.91 | - | WB present | 0 | 1 | *Firmicutes* | *Clostridia* | *Clostridiales* | *Ruminococcaceae* | *-* | *-* |
| OTU 245 | 2.29 | -0.15 | 0.83 | -0.18 | - | WB absent | 2 | 1 | *Synergistetes* | *Synergistia* | *Synergistales* | *Synergistaceae* | *-* | *-* |
| OTU 246 | 2.46 | 1.28 | 0.91 | 1.41 | - | WB absent | 1 | 0 | *Actinobacteria* | *Coriobacteriia* | *Coriobacteriales* | *Coriobacteriaceae* | *-* | *-* |
| OTU 247 | 2.24 | -1.17 | 0.89 | -1.32 | - | WB present | 0 | 1 | *Bacteroidetes* | *Bacteroidia* | *Bacteroidales* | *Rikenellaceae* | *-* | *-* |
| OTU 248 | 1.79 | 0.63 | 0.87 | 0.72 | - | WB absent | 1 | 0 | *Firmicutes* | *Clostridia* | *Clostridiales* | *Lachnospiraceae* | *-* | *-* |
| OTU 249 | 4.28 | -0.74 | 0.91 | -0.81 | 0.491 | WB present | 1 | 4 | Unassigned | *-* | *-* | *-* | *-* | *-* |
| OTU 250 | 8.64 | -2.75 | 1.13 | -2.42 | **0.048** | WB present | 1 | 2 | *Planctomycetes* | *vadinHA49* | *PeHg47* | *-* | *-* | *-* |
| OTU 251 | 6.64 | 2.81 | 1.17 | 2.40 | - | WB absent | 1 | 0 | *Firmicutes* | *Clostridia* | *Clostridiales* | *-* | *-* | *-* |
| OTU 252 | 3.70 | -2.01 | 1.02 | -1.97 | - | WB present | 0 | 1 | *Firmicutes* | *Clostridia* | *Clostridiales* | *Veillonellaceae* | *-* | *-* |
| OTU 253 | 1.67 | -0.48 | 0.88 | -0.54 | - | WB present | 0 | 1 | Unassigned | *-* | *-* | *-* | *-* | *-* |
| OTU 254 | 2.60 | 1.38 | 0.91 | 1.52 | - | WB absent | 2 | 0 | *Actinobacteria* | *Actinobacteria* | *Actinomycetales* | *Actinomycetaceae* | *Actinomyces* | *-* |
| OTU 255 | 2.00 | -0.93 | 0.87 | -1.07 | - | WB present | 0 | 1 | *Proteobacteria* | *Deltaproteobacteria* | *Desulfovibrionales* | *Desulfovibrionaceae* | *-* | *-* |
| OTU 256 | 2.26 | 0.69 | 0.86 | 0.80 | - | WB absent | 1 | 1 | *Proteobacteria* | *Alphaproteobacteria* | *Rhodospirillales* | *Rhodospirillaceae* | *-* | *-* |
| OTU 257 | 1.82 | -0.66 | 0.87 | -0.76 | - | WB present | 0 | 1 | Unassigned | *-* | *-* | *-* | *-* | *-* |
| OTU 258 | 2.71 | -0.79 | 0.88 | -0.90 | - | WB present | 2 | 1 | *Firmicutes* | *Clostridia* | *Clostridiales* | *Lachnospiraceae* | *Roseburia* | *-* |
| OTU 259 | 2.43 | 0.84 | 0.88 | 0.96 | - | WB absent | 1 | 1 | *Firmicutes* | *Clostridia* | *Clostridiales* | *Ruminococcaceae* | *-* | *-* |
| OTU 260 | 2.35 | 0.14 | 0.84 | 0.16 | - | WB absent | 1 | 1 | *Actinobacteria* | *Actinobacteria* | *Actinomycetales* | *Micrococcaceae* | *Kocuria* | *palustris* |
| OTU 261 | 4.64 | -2.34 | 1.03 | -2.27 | 0.061 | WB present | 0 | 2 | Unassigned | *-* | *-* | *-* | *-* | *-* |

^+^ Benjamini and Hochberg [2] (BH) adjusted *P* values < 0.05 are in bold.

^^^ Classification could not be made to this level

^*^ log_2_ fold change

1. Love MI, Huber W, Anders S. Moderated estimation of fold change and dispersion for RNA-seq data with DESeq2. Genome Biol. 2014;15:550. doi: 10.1186/s13059-014-0550-8.

2. Benjamini Y, Hochberg Y. Controlling the false discovery rate: a practical and powerful approach to multiple testing. J Roy Stat Soc Ser B (Stat Method). 1995;57(1):289-300.
